# First-principles prediction of the thermoelectric properties of Sn(S_0.2_Se_0.8_) alloys

**DOI:** 10.1039/d6cp02931k

**Published:** 2026-10-19

**Authors:** Min Zhang, Joseph M. Flitcroft, Jonathan M. Skelton

**Affiliations:** a Department of Chemistry, University of Manchester Oxford Road Manchester M13 9PL UK jonathan.skelton@manchester.ac.uk

## Abstract

Thermoelectric power is an efficient and flexible technology for improving energy efficiency, but widespread applications require cost-effective and sustainable materials with a high figure of merit *zT*. Alloying is a common strategy for optimising *zT*, but predicting and understanding its impact on the material properties is made challenging by the structural disorder. In this work, we develop a practical and rigorous approach to predicting the *zT* of alloys, by combining first-principles electrical-transport and lattice thermal-conductivity calculations with approximate models for the electron and phonon scattering, and apply it to study the Sn(S_0.2_Se_0.8_) alloy. We compare predictions obtained by averaging over the full set of unique configurations in a 32-atom supercell with those obtained from special quasi-random structure (SQS) structures with supercell sizes up to 256 atoms. We find that a 128-atom SQS model provides good computational efficiency while avoiding supercell size artifacts, and yields realistic predictions of the physical properties at the extrinsic carrier concentrations (“doping levels”) that optimise the *zT*. The calculations indicate that Sn(S_0.2_Se_0.8_) shows comparable performance to the SnSe endpoint but with a modest enhancement to the *zT* due to a reduction in the lattice thermal conductivity, which has a particularly significant impact on the low-temperature performance.

## Abstract 2

This work presents an efficient and accurate first-principles computational modelling workflow for predicting the thermoelectric figure of merit, *zT*, of alloys, and demonstrates its successful application to Sn(S_0.2_Se_0.8_).

## Graphical abstract

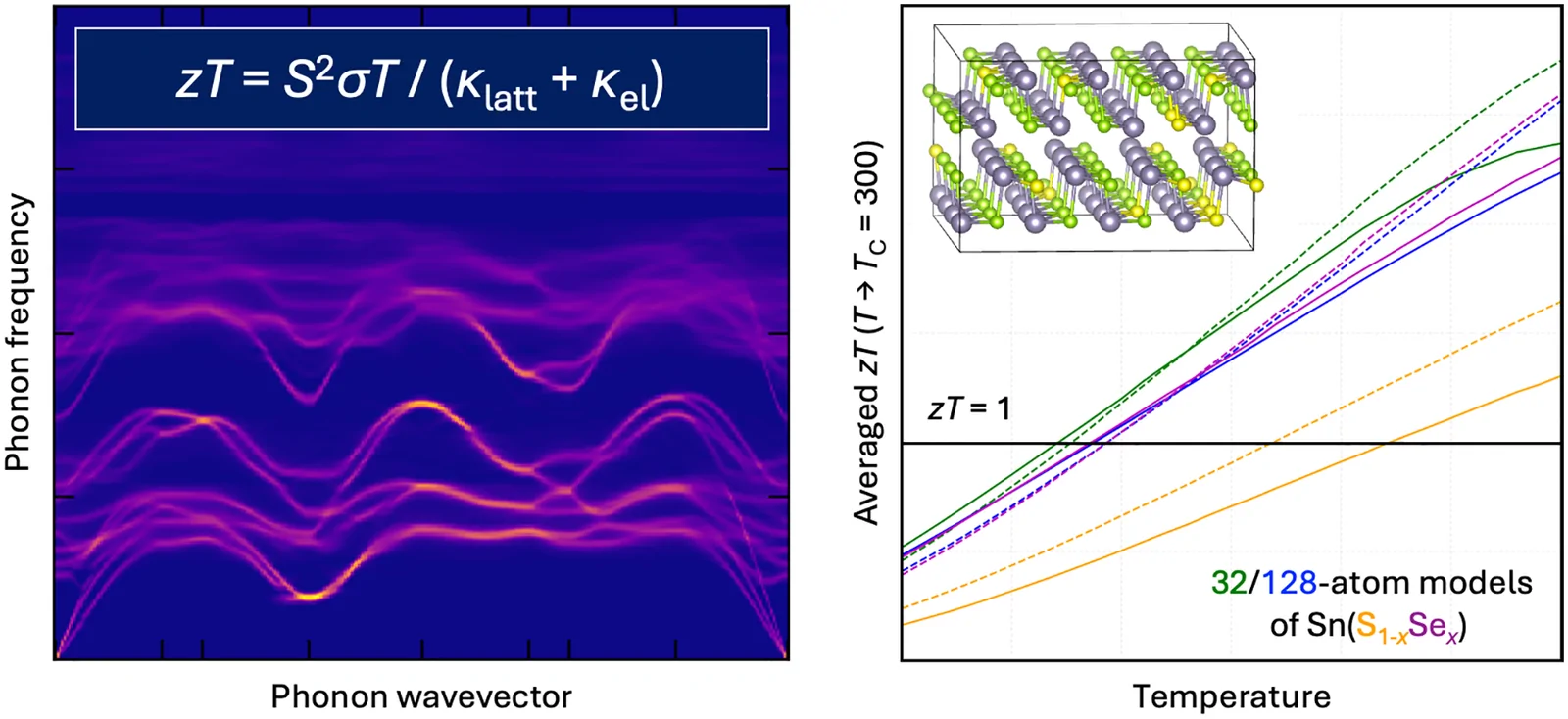

## 1 Introduction

60–70% of global energy is currently lost as heat, and technologies to improve energy efficiency and reduce global demand for fossil fuels are crucial enablers for the transition to net zero greenhouse gas emissions.^1–3^ Thermoelectric (TE) power, which enables the direct recovery of waste heat to electricity, is a promising technology to meet this need, and its demonstrated applications include energy-intensive processes that are particularly challenging to decarbonise such as heat recovery from automobile powertrains and industrial data centre facilities.^4,5^

Thermoelectric generators (TEGs) leverage the Seebeck effect in a matched pair of TE materials with dominant p-type (hole) and n-type (electron) transport, connected electrically in series and thermally in parallel, to extract energy from a temperature gradient. The performance of a TE material is typically described by the dimensionless figure of merit *zT* given by^6^:1
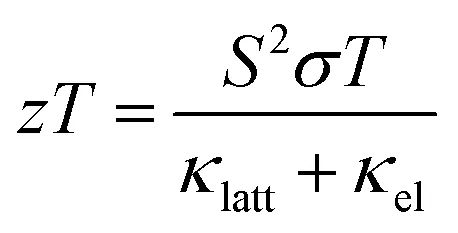
where *S* is the Seebeck coefficient, *σ* is the electrical conductivity, *S*^2^*σ* is the power factor (PF), and *κ*_latt_ and *κ*_el_ are the lattice (phonon) and electronic contributions to the total thermal conductivity *κ*. The electrical transport properties *S*, *σ* and *κ*_el_ depend on the electronic structure and can be balanced through the charge-carrier concentration *n*.^7^ The optimum balance between minimising *κ*_el_ and maximising the PF is usually found in heavily-doped semiconductors with *n* ≈ 10^19^–10^21^ cm^−3^.^7^ The lattice thermal conductivity is generally considered to be independent of the electronic structure, and low *κ*_latt_ is associated with materials composed of heavy atoms, with weak and/or inhomogeneous chemical bonding, and with strong anharmonic phonon scattering.^8,9^ The *κ*_latt_ can also be modulated by atomic-scale point defects and by nano- and microstructuring, with the required length scales dependent on the characteristic wavelengths and mean-free paths of the heat-carrying phonon modes.^10^

Tin monoselenide (SnSe) has received significant attention as a promising TE material due to its favourable electronic structure and intrinsically low *κ*_latt_,^8,11–13^ and the high elemental abundance and relatively low toxicity of Sn and Se. Despite achieving record *zT* ≈ 2.6 in bulk and *zT* > 3 in polycrystalline powders,^14,15^ the best performance is obtained at high temperature, and the low-temperature performance is not competitive with the industry-standard Bi_2_Te_3_ (*zT* up to ∼1.5 from 300–400 K^16,17^). Given that the majority of waste heat is released below 100 °C,^6,18,19^ improving the low-temperature performance is critical for widespread application.

Alloying to form solid solutions is an established technique for improving the performance of TE materials.^20–26^ Alloying can reduce the thermal conductivity,^20–23^ modulate the electronic band structure to improve the power factor,^24,25^ and improve chemical compatibility with dopants (“dopability”).^26^ SnS and SnSe crystallise into an isostructural orthorhombic phase (*Pnma* spacegroup) under ambient conditions and transform into a higher-symmetry *Cmcm* phase at high temperature.^27^ The second-order phase transition results in both systems displaying similar anharmonic lattice dynamics.^8,13,28^ The two materials also have similar bandgaps and dielectric properties.^29–32^ This similarity makes the formation of Sn(S_1−*x*_Se_*x*_) solid solutions facile, and various experimental studies have indicated that the alloys can show lower lattice thermal conductivity and improved *zT* compared to the SnS and SnSe endpoints.^33–35^

Advances in theoretical techniques, software and computing power over the past decade have made it possible to accurately and efficiently predict all four properties in eqn (1), and the *zT*, using first-principles modelling techniques such as density-functional theory (DFT).^36–41^ We have developed an approach that combines models for the structural dynamics and electrical transport, including descriptions of the phonon and electron scattering, and successfully benchmarked it against *Pnma* SnS and SnSe, the related Ge chalcogenides, and, more recently, the novel “π” phases of the Sn monochalcogenides.^42–44^ More generally, modelling has also played a significant role in understanding the structure–property relationships that underpin high thermoelectric performance, paving the way toward the theory-led design of new materials.^9,44–49^ However, while the theoretical methodology for studying bulk materials is well established, solid solutions and heavily-doped materials remain a significant challenge. We have previously predicted the bandgaps and dielectric properties of Sn(S_1−*x*_Se_*x*_) solid solutions by explicitly enumerating the symmetry-independent configurations of various chalcogen compositions in small supercell models,^50^ and we have more recently applied a similar approach to model the electronic structure of Ge(S_0.5_Se_0.5_).^51^ We have also developed an approach to predicting the *κ*_latt_ of solid solutions, which we successfully applied to Sn(S_0.1875_Se_0.8125_) and, subsequently, to explicit atomistic models of Ag-doped SnS.^52,53^ Another group have also developed a complementary approach using machine-learnt interatomic potentials and molecular-dynamics simulations, and applied it to model the thermal transport in Sn(S_0.75_Se_0.25_).^54^ However, the prediction of the *S*, *σ* and *κ*_el_, which are required to evaluate the *zT*, has yet to be addressed.

In this work, we develop a complete workflow for predicting the *zT* of solid solutions and apply it to the *Pnma* Sn(S_1−*x*_Se_*x*_) system, focusing on compositions close to the best-performing Sn(S_0.2_Se_0.8_) identified in experiments.^33–35^ We compare our previous approach based on explicitly modelling the symmetry-independent configurations in a small supercell of the parent structure^50,55^ to using single disordered supercells with 32–256 atoms generated using the special quasi-random structure (SQS) method.^56^ We combine our previous approach of modelling the lattice dynamics and *κ*_latt_^52^ with a methodology for calculating the electrical transport properties with approximate models for the electron scattering rates.^39^ We find that a single 128-atom SQS supercell provides an optimal trade-off between avoiding supercell size artifacts while providing realistic predictions of the transport properties and *zT* at a manageable computational cost. Our calculations demonstrate that Sn(S_0.2_Se_0.8_) can show lower *κ*_latt_ and comparable or larger maximum *zT*, *zT*_max_, than the SnS and SnSe endpoints. In particular, we predict an averaged high-temperature n-type *zT*_max_ = 2.4, which is 50% larger than n-type SnS and comparable to n-type SnSe, and a room-temperature *zT*_max_ = 0.62 around 22% higher than SnSe and 2.4× higher than SnS. Our methodology enables facile prediction of the thermoelectric performance of a wide range of homogeneous alloy thermoelectrics, and provides a route to using modelling to support experimental composition optimisation.

## 2 Computational methods

### 2.1 Alloy models

#### 2.1.1 Systematic averaging over configurations

An initial model for the Sn(S_0.2_Se_0.8_) alloy was constructed using the approach adopted in our previous studies.^50,52,57^

The complete set of 23 symmetry-independent configurations in a 32-atom 2 × 1 × 2 supercell of the *Pnma* SnS parent structure, with composition Sn(S_0.1875_Se_0.8125_), was generated using our in-house Transformer code.^58^ Each of the *n* = 23 structures was fully optimised, and the lattice thermal conductivity, electrical-transport properties and *zT* were calculated as described below.

The occurrence probabilities *P*_*i*_ of each structure were then computed at a reference formation temperature *T*_F_ = 900 K according to:2
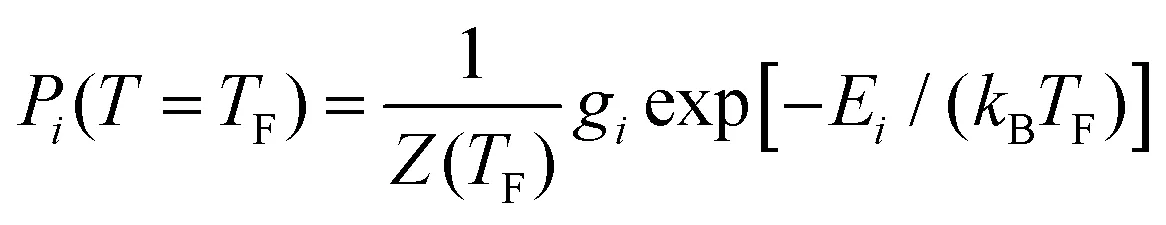
*E*_*i*_ and *g*_*i*_ are the total energies and degeneracies of the structures, and *k*_B_ is the Boltzmann constant. *Z*(*T*) is the thermodynamic partition function given by:3
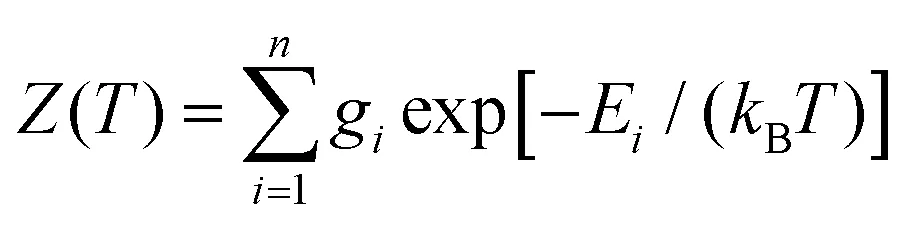


We note that we are equating the *E*_*i*_ to the lattice energies *U*^latt^_*i*_, and that we do not account for the phonon contributions to the free energy, as we previously found these to have little impact on the distribution of *P*_*i*_ in this system with appropriately-converged phonon frequencies.^52^ Finally, weighted averages *X̄* of the calculated properties *X* were taken using the *P*_*i*_ according to:4
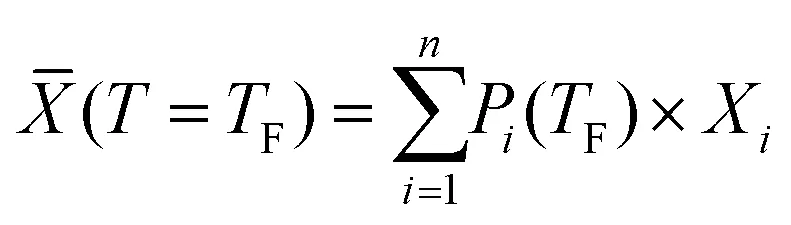


The main advantages of this approach are that it accounts for all the independent atomic configurations in the chosen supercell and provides access to thermodynamic information including the free energy of mixing and the configurational entropy. On the other hand, the key downside is that the number of configurations scales combinatorially with the supercell size, which severely restricts the size of the model that can be considered, and small supercells may impose an artificial periodicity and/or may not provide a realistic description of the medium- and long-range order in a real alloy.

#### 2.1.2 The special quasi-random structure (SQS) method

As an alternative to the systematic averaging, we also explored the special quasi-random structure (SQS) method.^56^

The idea behind the SQS approach is to represent a disordered material with a single, optimal supercell configuration. The occupations of the *N* alloying lattice sites *i* are denoted by a “spin” *σ*_*i*_ (after the Ising model, with *σ*_*i*_ = ±1 typically used for sites with two occupying atoms). The configuration is then discretised into geometric “figures” *f* = (*k*, *m*) composed of *k* vertices separated by an *m*th neighbour distance (*e.g.*, *k* = 2, *m* = 1 corresponds to first-neighbour pairs). A series of correlation functions Π̄_*k*,*m*_ is then constructed from the average products of the *σ*_*i*_ over the lattice sites in the full set of symmetry-equivalent figures located at position ***l***. For a perfectly disordered binary system with composition *x*, the expectation values of the correlation functions are given by:

5 〈Π̄_*k*,*m*_〉 = (2*x* − 1)^*k*^

The objective of the SQS is to arrange the alloying atoms in a chosen supercell such that the Π̄_*k*,*m*_ match the 〈Π̄_*k*,*m*_〉 for the perfectly random system as closely as possible.

The advantage of the SQS over systematic averaging is that it yields a single structure for a given composition and supercell size, but the downside is that a larger supercell is likely to be required in order to capture a sufficient diversity of local atomic environments. To this end, we generated SQS models with 32, 72, 128 and 256 atoms, corresponding to 2 × 1 × 2, 3 × 1 × 3, 4 × 1 × 4 and 4 × 2 × 4 expansions of the *Pnma* SnS parent structure. The smaller 32- and 72-atom cells have chalcogen compositions of 81.3 and 80.6% Se, respectively, and the two larger cells both have 79.7% Se. We note that the SQS assumes ideal mixing behaviour, *i.e.*, the method purposely avoids favouring specific local atomic environments. This is appropriate for systems that show (thermodynamically) near-ideal mixing, where the enthalpy of formation Δ*H*_F_ is much lower than the available thermal energy and the free energy is dominated by the entropy. Previous calculations have shown this to be the case for Sn(S_1−*x*_Se_*x*_) at realistic formation temperatures,^50,59^ so the SQS should be appropriate for this system.

The SQS structures were generated using the Monte-Carlo algorithm^60^ implemented in the Alloy Theoretic Automated Toolkit (ATAT),^61^ taking into account pair clusters with a cutoff of 8.7 Å and triplet, quadruplet and quintuplet clusters with a cutoff of 6 Å to describe the disorder. Using this procedure, the correlation function of the 256-atom SQS model differed from that of the ideal random alloy by <0.1, while those of the smaller 32- and 72/128-atom models differed by <0.7 and <0.2 respectively. Each of the four SQS structures was fully optimised and the thermoelectric properties were calculated in the same way as for the set of 32-atom supercell models generated with the systematic averaging approach, using the procedure outlined in the following subsection. We note that we optimised these cutoffs against the correlation function, and we did not test the convergence of the physical properties with respect to these parameters. While this is in principle possible, the properties in the eqn (1) are relatively demanding to compute, and we expect that most users adopting the present methodology would not attempt to do so.

### 2.2 Prediction of *zT*

Our approach to predicting the *zT* of thermoelectric materials from first principles has been described extensively in our previous work^42–44^ and is briefly summarised here for completeness.

#### 2.2.1 Phonon spectra and lattice thermal conductivity

The macroscopic ***κ***_latt_ is the sum of the particle-like contribution ***κ***_p_ and the wave-like contribution ***κ***_w_ from intraband tunnelling^62^:

6 ***κ***_latt_(*T*) = ***κ***_p_(*T*) + ***κ***_w_(*T*)

The ***κ***_p_ was calculated using the single mode-relaxation time approximation (SM-RTA), as a sum of contributions from phonon modes ***q****j*, according to^37^:7
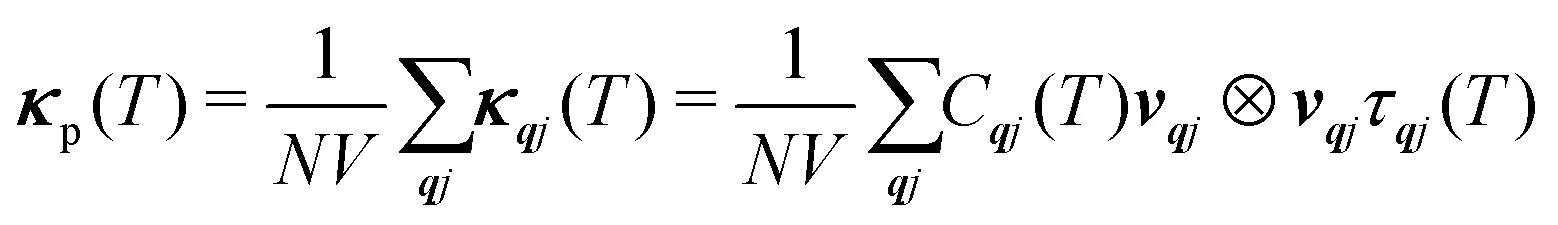
where ***q*** and *j* are the phonon wavevector and band index, *C*_***q****j*_, ***v***_***q****j*_ and *τ*_***q****j*_ are the modal heat capacities, group velocities and lifetimes, *V* is the unit-cell volume, and *N* is the number of ***q*** included in the summation (equivalent to the number of unit cells in the crystal). The *C*_***q****j*_ and ***v***_***q****j*_ are calculated within the harmonic approximation using the 2nd-order interatomic force constants (FCs) ***Φ***(2). The ***τ***_***q****j*_ are the inverse of the phonon linewidths Γ_***q****j*_:8
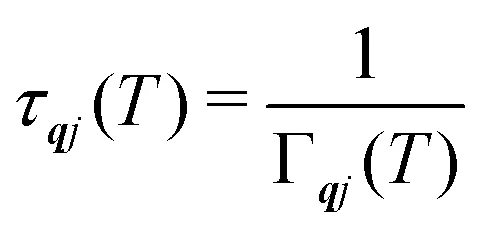


The Γ_***q****j*_ can be obtained perturbatively at third-order by combining the phonon frequencies *ω*_***q****j*_ and displacement vectors ***W***_***q****j*_ calculated within the harmonic approximation with the 3rd-order (anharmonic) force constants ***Φ***(3).^37^

Computing the ***Φ***(2) is relatively inexpensive, and it is therefore straightforward to calculate the *C*_***q****j*_ and ***v***_***q****j*_ of the various alloy models. However, the number of atomic displacements required to evaluate the ***Φ***(3) increases rapidly with the cell size, and it is not practical to obtain the *τ*_***q****j*_ using this approach. For the majority of the alloy models we therefore instead adopt the method developed in our previous work.^52^ The phonon linewidths can be written approximately as^37^:9
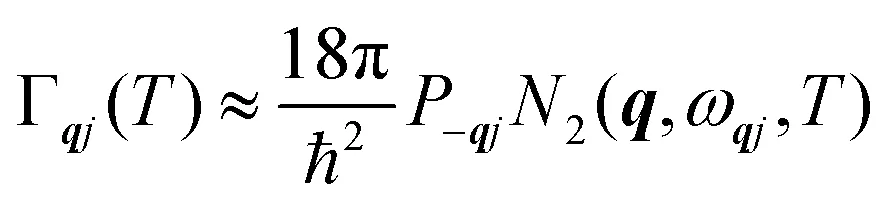
where *ħ* is the reduced Planck constant, *P*_***q****j*_ is the averaged three-phonon interaction strength, and the function *N*_2_ is a weighted two-phonon joint density of states (w-JDoS) that counts the number of energy- and momentum-conserving three-phonon scattering pathways available to a phonon with wavevector ***q*** and frequency *ω*_***q****j*_. The *P*_***q****j*_ are given by:10
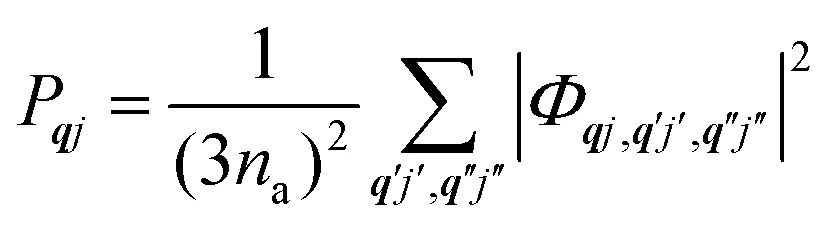
where *n*_a_ is the number of atoms in the primitive cell (*i.e.*, there are 3*n*_a_ bands at each ***q***) and *Φ*_***q****j*,***q***′*j*′,***q***″*j*″_ are the three-phonon interaction strengths.^37^ The *N*_2_ are calculated by summing the number of pathways for collision (Type 1) and decay (Type 2) processes^37^:

11 *N*_2_(***q***,*ω*,*T*) = *N*^(1)^_2_(***q***,*ω*,*T*) + *N*^(2)^_2_(***q***,*ω*,*T*)

12
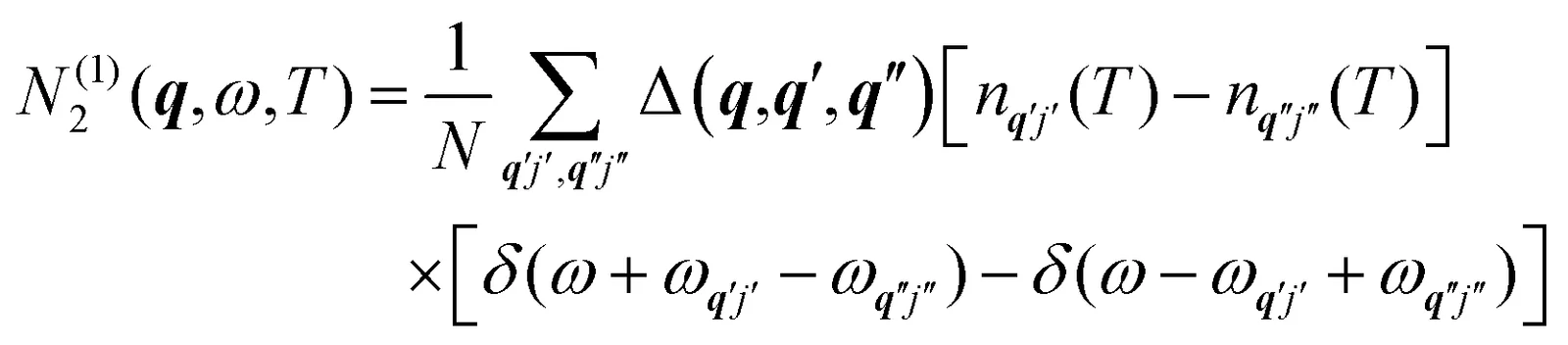
13
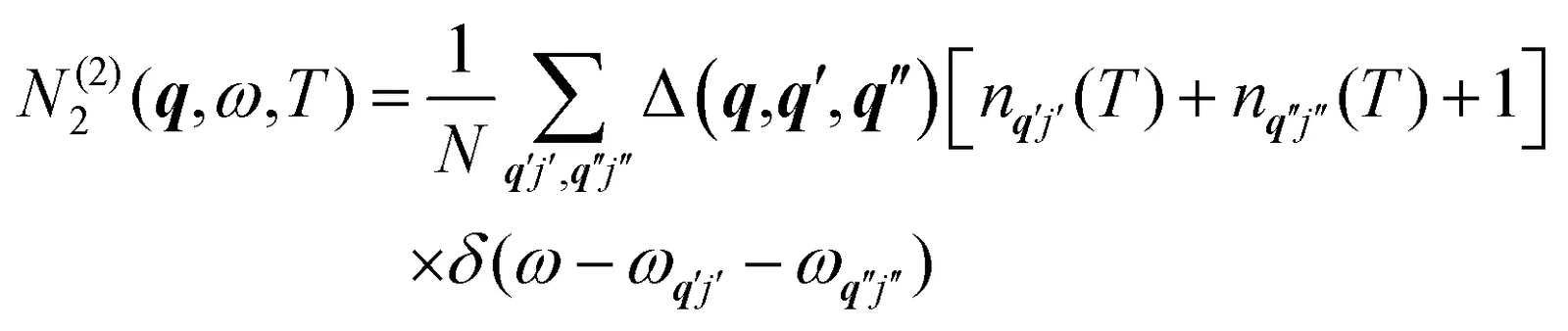


The functions *δ* and Δ enforce the conservation of energy and (crystal) momentum, respectively, for three-phonon scattering processes, and the *n*_***q****j*_ are the phonon occupation numbers from the Bose–Einstein distribution:14
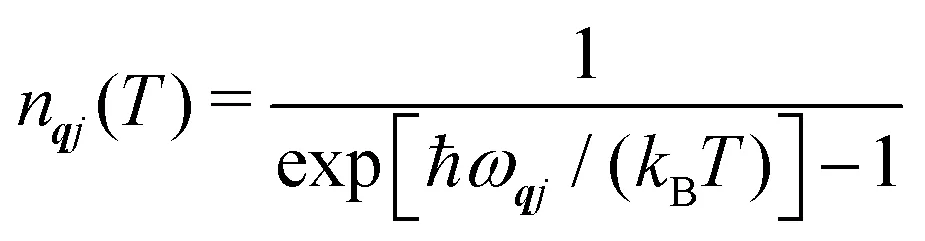


The *N*_2_ depend only on the *ω*_***q****j*_ and are therefore calculable within the harmonic approximation. The Γ_***q****j*_ can therefore be obtained without explicit calculation of the **Φ**(3) by replacing the *P*_***q****j*_ in eqn (9) with a constant value *P̃*. Suitable values for the alloys were obtained by linear interpolation and appropriate scaling of the *P̃* determined for the *Pnma* SnS and SnSe endpoints in previous work.^52^

While SM-RTA model in eqn (7) provides a closed-form solution to the phonon Boltzmann transport equation (BTE) for the ***κ***_p_, obtaining the ***κ***_w_ requires solving the Wigner transport equation (WTE). In this formalism, the ***κ***_w_ is given by^62^:15
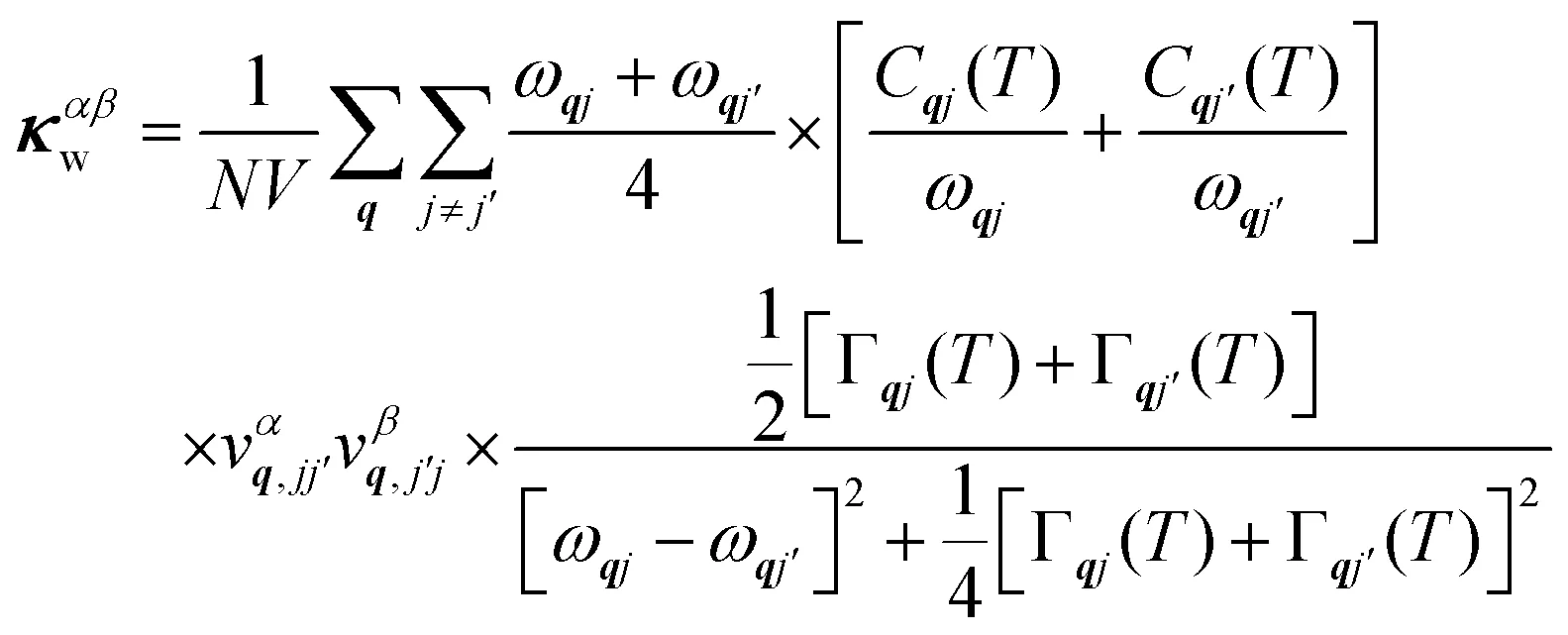
where ***v***_***q***,*jj*′_ are the matrix elements of the velocity operator in the phonon mode basis. This expression can be evaluated using the harmonic phonon modes and the approximate Γ_***q****j*_ from eqn (9), *i.e.*, in a consistent manner to computing the ***κ***_p_ using the RTA with the approximate *τ*_***q****j*_ obtained from eqn (8).

We have previously shown that the ***κ***_w_ term makes an important contribution to the ***κ***_latt_ of the structurally-complex “π” phases of SnS and SnSe and “dilute” supercell models of Ag-doped SnS.^44,53^ In particular, we found that accounting for this contribution is necessary to recover the convergence of the ***κ***_latt_ of Ag-doped SnS to the bulk value as the doping level is reduced by progressively increasing the supercell size.^53^ Therefore, while we did not test this in our previous work on the Sn(S_0.2_Se_0.8_) alloy,^52^ we would expect the wave-like conductivity to be important for the alloys. Accounting for the ***κ***_w_ is a relatively straightforward extension of our previous work,^52,53^ and we explore this further below.

#### 2.2.2 Electronic transport properties

The three electrical transport properties ***S***, ***σ*** and ***κ***_el_ can be obtained in a similar manner to the particle-like contribution to the ***κ***_latt_ in eqn (7), from the solution to the electron BTE within the momentum relaxation-time approximation (MRTA).^38,39^

We first calculate the spectral conductivity **Σ** and generalised moments of the transport distribution function ***L***^*n*^(*T*) given by:16
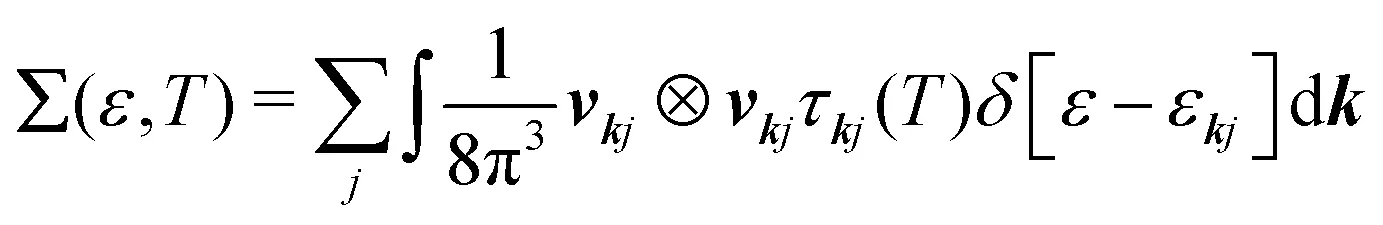
17
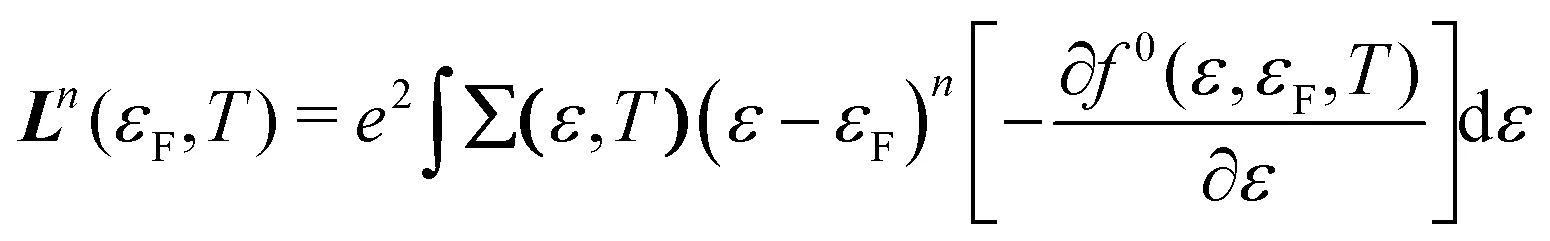
where *e* is the elementary charge, ***k*** and *j* are the electron wavevectors and band indices, respectively, and ***v***_***k****j*_, *τ*_***k****j*_ and *ε*_***k****j*_ are the electron group velocities, lifetimes and band energies. *f*^0^(*ε*, *T*) is the Fermi–Dirac distribution function given by:18
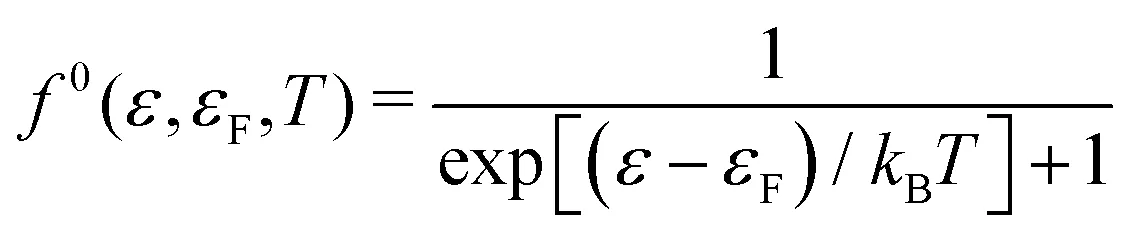
where *ε*_F_ is the Fermi energy. The *ε*_***k****j*_ and ***v***_***k****j*_ can be obtained from a high-quality electronic structure calculation. The *τ*_***k****j*_ can be obtained from electron–phonon coupling calculations in a similar manner to the *τ*_***q****j*_ for phonons, *i.e.*, by considering three-particle interactions between electrons and phonons. However, these are currently difficult to access and not widely used, particularly for structurally-complex systems such as alloys. Instead, we adopt the approach in ref. 39, where the *τ*_***k****j*_ are calculated from phenomenological expressions for the rates of common “universal” semiconductor scattering processes, *viz.* acoustic deformation potential (ADP), polar-optic phonon (POP), ionised impurity (IMP) and piezoelectric (PIE) scattering:

19 *τ*_***k****j*_^−1^ = (*τ*^ADP^_***k****j*_)^−1^ + (*τ*^POP^_***k****j*_)^−1^ + (*τ*^IMP^_***k****j*_)^−1^ + (*τ*^PIE^_***k****j*_)^−1^

The ***σ***, ***S*** and ***k***_el_ are then calculated from the ***L***^*n*^ as:

20 ***σ***(*ε*_F_,*T*) = ***L***^0^(*ε*_F_,*T*)

21
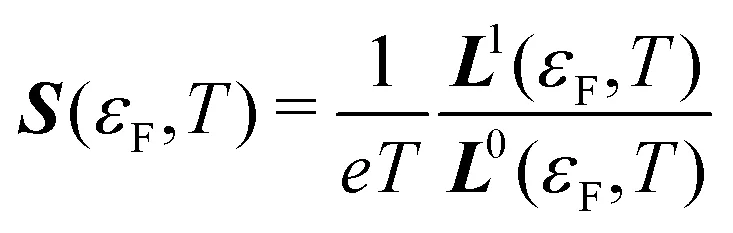
22
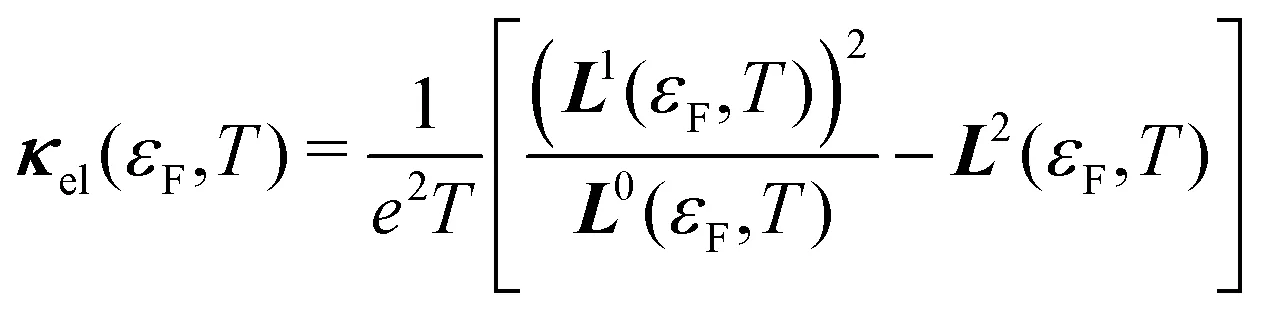


Under the assumption that chemical doping has a minimal effect on the electronic band structure, the *ε*_F_ can be adjusted to impose an extrinsic carrier concentration (“doping level”) *n* (termed the “rigid band” approximation, RBA).^63,64^ This approach therefore allows the electrical properties to be predicted as a function of *n* and *T*, and we have previously obtained accurate results for *Pnma* SnS and SnSe and for other related Group IV–VI monochalcogenides.^42–44^

In practice, this approach to predicting the electrical properties requires a high-quality electronic-structure calculation to obtain an accurate electronic bandgap *E*_g_ and band energies *ε*_***k****j*_, together with calculations of the various material properties required to estimate the *τ*_***k****j*_.^39^ One would typically use a hybrid functional (*e.g.*, HSE06^65,66^) to compute the *E*_g_ and *ε*_***k****j*_, but this can be challenging for large unit cells and high ***k***-point densities. To mitigate this, we adopt the non-self-consistent hybrid approach developed in our previous work,^67^ where we compute both quantities using the Kohn–Sham orbitals from a PBE^68^ calculation.

We also note that, to the best of our knowledge, there is no direct analogue of the Wigner formulation for phonon transport for electrons. This theoretical description of the electrical properties is therefore strictly based on particle-like transport, and we discuss the implications of this below.

#### 2.2.3 Thermoelectric figure of merit

Finally, the ***κ***_latt_(*T*) can be combined with the ***S***(*n*, *T*), ***σ***(*n*, *T*) and ***κ***_el_(*n*, *T*) to estimate the figure of merit *zT* as a function of doping level and temperature. The four properties entering eqn (1) are tensor quantities, and the figure of merit is therefore orientation dependent. The *zT* along a particular Cartesian direction *α* ∈ {*x*,*y*,*z*} can be calculated as:23
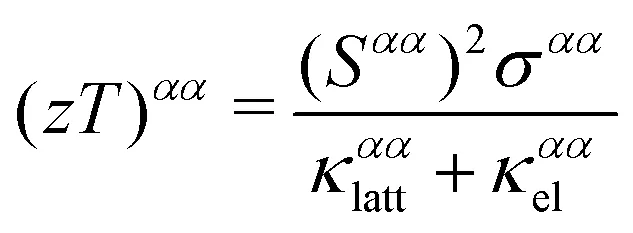


This mirrors the way in which the figure of merit is calculated from experimental measurements of the constituent properties. Alternatively, for pressed pellets with randomly-oriented crystallites, the four properties can be replaced by their scalar averages *S*, *σ*, *κ*_latt_ and *κ*_el_ given by, *e.g.*,:24
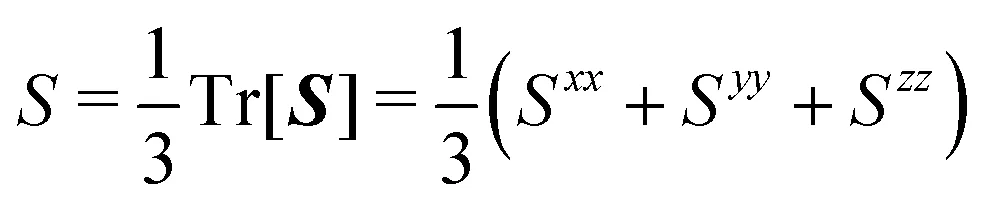


The power factors *S*^2^*σ* in the numerator of the *zT* equation are calculated in an analogous manner, *i.e.*, from the *S*^*αα*^ and *σ*^*αα*^ along a given direction or from the scalar-averaged *S* and *σ*.

### 2.3 First-principles calculations

DFT calculations were carried out using periodic pseudopotential plane-wave density-functional theory (DFT) as implemented in the Vienna *Ab initio* Simulation Package (VASP) code.^70^

Electron exchange and correlation were treated using the dispersion-corrected PBEsol + D3 functional^71,72^ used in several of our previous studies on these and similar materials.^43,44,50,73,74^ The core electrons were modelled using projector-augmented wave (PAW) pseudopotentials^75,76^ with the Sn 5s/5p, the S 3s/3p, and Se 4s/4p electrons in the valence region. We note that we have previously used a Sn pseudopotential including the semi-core Sn 4d electrons in the valence region, but we found that incorporating these into the pseudopotential core significantly reduced the computational cost of the calculations while having a minimal effect on the total energy, optimised lattice parameters, and electronic bandgap (Table S1). The valence electronic structure was modelled using a plane-wave basis with a cutoff of 600 eV and the Γ-centred Monkhorst–Pack ***k***-point meshes^69^ listed in Table 1.

**Table 1:** Technical parameters used in the calculations performed in this work. For each of the five Sn(S_0.2_Se_0.8_) models we examine, we list the “base” and “dense” ***k***-point sampling meshes used for selected electronic-structure calculations (see text), the supercell and reduced ***k***-point sampling used to evaluate the second-order force constants **Φ**(2), the supercell, reduced ***k***-point sampling, range cutoff and estimated average three-phonon interaction strengths *P̃* used to evaluate the third-order force constants **Φ**(3) and the three-phonon interaction strengths *Φ*, where applicable, and the ***q***-point sampling used to compute the phonon density of states *g*(*f*) and the lattice thermal conductivity ***k***_latt_. ***k***- and ***q***-point meshes are specified as the number of subdivisions on regular Γ-centred Monkhorst–Pack grids^69^

	***k***-Point sampling		**Φ**(2)		**Φ**(3)		***q***-Point sampling	
	Base	Dense	Supercell (# Atoms)	***k***-Points	Cutoff/Å	*P̃*/eV^2^	*g*(*ω*)	***κ***_latt_
32-atom (enum)	4 × 3 × 5	8 × 6 × 10	2 × 2 × 2 (256)	2 × 2 × 3	—	1.47 × 10^−12^	24 × 24 × 24	9 × 9 × 9
32-atom (SQS)	4 × 3 × 5	8 × 6 × 10	2 × 2 × 2 (256)	2 × 2 × 3	—	1.47 × 10^−12^	24 × 24 × 24	9 × 9 × 9
72-atom (SQS)	3 × 3 × 3	6 × 6 × 6	2 × 2 × 2 (576)	2 × 2 × 2	4.5	2.93 × 10^−13^	24 × 24 × 24	11 × 11 × 11
128-atom (SQS)	2 × 3 × 3	6 × 9 × 9	1 × 2 × 1 (256)	2 × 2 × 3	—	9.36 × 10^−14^	12 × 24 × 12	11 × 11 × 11
256-atom (SQS)	2 × 2 × 3	6 × 6 × 9	1 × 1 × 1 (256)	2 × 2 × 3	—	2.34 × 10^−14^	12 × 12 × 12	11 × 11 × 11

All structures were fully optimised to strict tolerances of 10^−8^ eV on the total energy and 10^−2^ eV Å^−1^ on the forces. Second-order (harmonic) force constants **Φ**(2) and, for selected systems, third-order (anharmonic) force constants **Φ**(3) were computed using the supercell finite-displacement method with the Phonopy^36^ and Phono3py^37^ codes, respectively. The **Φ**(2) and **Φ**(3) were calculated using the default displacement steps of 10^−2^ and 3 × 10^−2^ Å and with the supercells, reduced ***k***-point sampling, and, where appropriate, interatomic distance cutoffs listed in Table 1. Phonon density of states curves *g*(*f*) were obtained by interpolating the frequencies onto the uniform Γ-centred ***q***-point meshes listed in Table 1. Phonon dispersions were obtained by using the band-unfolding approach in ref. 77 to project phonon modes back to the Brillouin zone of the closest *Pnma* SnSe endpoint. Non-analytical corrections were applied to describe splitting between longitudinal and transverse optic modes (LO/TO splitting) using the method in ref. 78 and with high-frequency dielectric constants ***ε***_∞_ and Born charges Z* computed using density-functional perturbation theory (DFPT).^79^ For the smaller 32- and 72-atom SQS models, the phonon lifetimes were computed by combining the harmonic frequencies and eigenvectors with the **Φ**(3) calculated as outlined in Table 1. For these and the larger models the lifetimes were (also) estimated using the approach in our previous work^52^ with the scaled average three-phonon interaction strengths in Table 1. The ***k***_latt_ was evaluated using modal properties computed on the ***q***-point grids listed in Table 1.

The electronic transport properties were obtained using the AMSET code.^39^ Accurate band gaps *E*_g_ and high-frequency dielectric constants ***ε***_∞_ were obtained using non-self-consistent HSE06^66,67^ in conjunction with the linear-optics routines in VASP.^79^ These *E*_g_ were then used to correct uniform band structures evaluated on the “dense” ***k***-point meshes listed in Table 1. We note that while the typical 2× enhancement factor used in previous work was found to be sufficient for the smaller 32- and 72-atom models, a larger 3× enhancement was required for the 128- and 256-atom models to accurately capture the dispersion and carrier effective masses (Table S2 and Fig. S1–S4). AMSET calculates electronic relaxation times by considering four scattering mechanisms, *viz.* acoustic deformation potential (ADP), piezoelectric (PIE), polar optical phonon (POP) and ionised impurity (IMP) scattering, which require the calculation of several other properties. The band deformation potentials ***D***_***k****j*_ were computed from a series of single-point energy calculations on deformed structures, generated with AMSET, using the “base” ***k***-point meshes. The elastic constant matrices ***C*** were obtained using the finite differences approach in VASP.^80^ The static dielectric constants ***ε***_S_ = ***ε***_∞_ + ***ε***_ionic_ were obtained by combining the HSE06 ***ε***_∞_ with ***ε***_ionic_ derived from the calculated phonon frequencies and eigenvectors at ***q*** = Γ, the Z*, and an implementation of the procedure in ref. 81 in our Phonopy-Spectroscopy package.^82^ Finally, the piezoelectric stress tensor ***e*** was computed from the same DFPT approach used to obtain the Z* for the non-analytical correction to the phonon frequencies.^79^

## 3 Results and discussion

### 3.1 Structure, dynamics and lattice thermal conductivity

The optimised structure of the 128-atom SQS model of *Pnma* Sn(S_0.2_Se_0.8_) is shown in Fig. 1, and those of the 32-, 72- and 256-atom SQS models are shown in Fig. S5. The corresponding optimised lattice constants, referenced back to the parent *Pnma* structure, are shown in Table 2. For the four SQS models, these are simply the optimised lattice parameters divided by the supercell expansion along the corresponding direction. For the 32-atom supercell enumeration model, the optimised lattice parameters are first averaged according to eqn (2) for a typical alloy formation temperature *T*_F_ = 900 K and then divided by the supercell expansion. The lattice parameters obtained for all five models are very similar and fall between those of the SnS and SnSe endpoints, and closer to SnSe, as expected from Vegard's law.

**Fig. 1:**
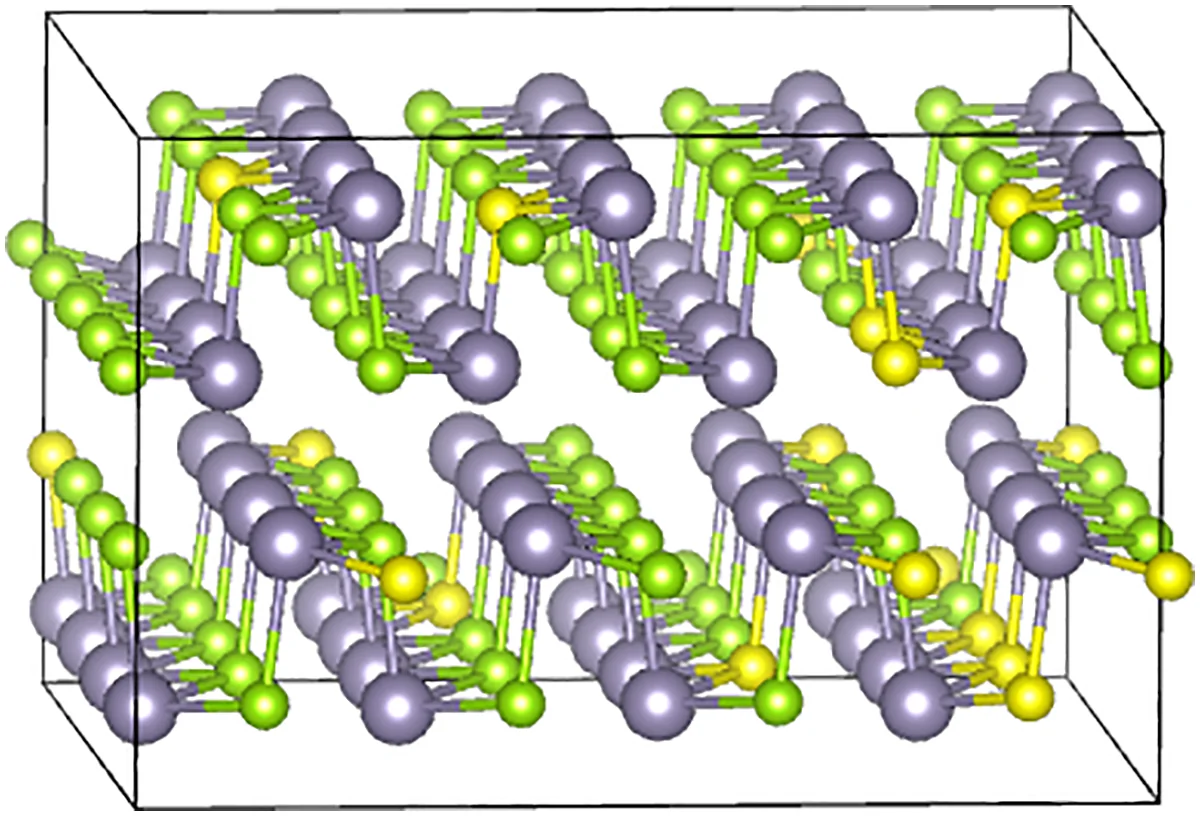
Optimised structure of the 128-atom special quasi-random structure (SQS) model of Sn(S_0.2_Se_0.8_). The Sn, S and Se atoms are shown in purple, yellow and green respectively. This image was prepared using the VESTA software.^83^

**Table 2:** Optimised lattice parameters and calculated formation enthalpies Δ*H*_F_ of the five Sn(S_0.2_Se_0.8_) models examined in this work. The lattice parameters are given in terms of the parent *Pnma* SnS/SnSe structures and compared to values for the endpoints from similar calculations.^42^ Unless otherwise stated the properties for the 32-atom supercell enumeration model are calculated for a formation temperature *T*_F_ = 900 K

	*a* [Å]	*b* [Å]	*c* [Å]	*V* [Å^3^]	Δ*H*_F_ [meV per F.U.]
SnS	4.22	10.99	3.96	183.66	—
Sn(S_0.187_Se_0.813_) (32-atom, enum)	4.30	11.30	4.09	198.96	5.87 (5.39 at *T*_F_ = 10 K)
Sn(S_0.187_Se_0.813_) (32-atom, SQS)	4.30	11.30	4.10	199.22	5.90
Sn(S_0.194_Se_0.806_) (72-atom, SQS)	4.30	11.29	4.09	198.56	5.86
Sn(S_0.203_Se_0.797_) (128-atom, SQS)	4.30	11.28	4.09	198.38	5.79
Sn(S_0.203_Se_0.797_) (256-atom, SQS)	4.30	11.28	4.09	198.38	4.43
SnSe	4.34	11.35	4.12	202.95	—

Table 2 also shows the calculated formation enthalpies calculated according to:

25 Δ*H*_F_ = *E*_Sn(S_1−*x*_Se_*x*_)_ − [(1 − *x*)*E*_SnS_ + *xE*_SnSe_]

where the *E* are the DFT total energies of the alloy and the SnS/SnSe endpoints, which are equivalent to the enthalpies *H* when relaxed to *p* ≈ 0. For the 32-atom supercell enumeration model, the *E* for the alloy is again obtained from a thermodynamic average, and we obtain values of Δ*H*_F_ = 5.39 and 5.87 meV per F.U. respectively at *T*_F_ = 10 and 900 K. The latter is very similar to the values of Δ*H*_F_ = 5.79–5.9 meV per F.U. obtained for the 32-, 72- and 128-atom SQS models, whereas the low-temperature Δ*H*_F_ lies between those of the three smaller SQS cells and the lower Δ*H*_F_ = 4.43 meV per F.U. obtained for the larger 256-atom model. Nevertheless, all five models yield the same qualitative prediction of a small positive formation enthalpy, in line with previous calculations.^50,59^

At the typical (higher) *T*_F_ = 900 K we predict a formation free energy Δ*G*_F_ = −24.8 meV per F.U. and a configurational entropy of *TS*_conf_ = 30.7 meV per F.U. for the 32-atom supercell enumeration model. Under the assumption of ideal mixing behaviour, a “first-order” value of *TS*_conf_ for the four SQS models can be estimated from:

26 *TS*_conf_ = −*RT*[*x *ln *x* + (1 − *x*)ln(1 − *x*)]

These ideal values range from 37.4–39.1 meV per F.U., which are 24–28% larger than that calculated for the supercell enumeration model. The Δ*H*_F_ are therefore sufficiently large in this system that an explicit average over configurations is necessary for a correct description of the thermodynamics. Nonetheless, we note that the predicted Δ*H*_F_ is well below *k*_B_*T* ≈ 25 meV atom^−1^ at room temperature, and so we would not expect spontaneous phase separation except at very low temperatures where the atomic diffusion would likely be too slow for this to be kinetically feasible.

The unfolded phonon dispersion and atom-projected density of states (PDoS) curves of the five alloy models are compared in Fig. 2. There are no imaginary modes in the unfolded phonon dispersions of any of the alloy models, which confirms that all 23 structures in the 32-atom supercell enumeration model, and all four of the SQS models, are dynamically stable.

**Fig. 2:**
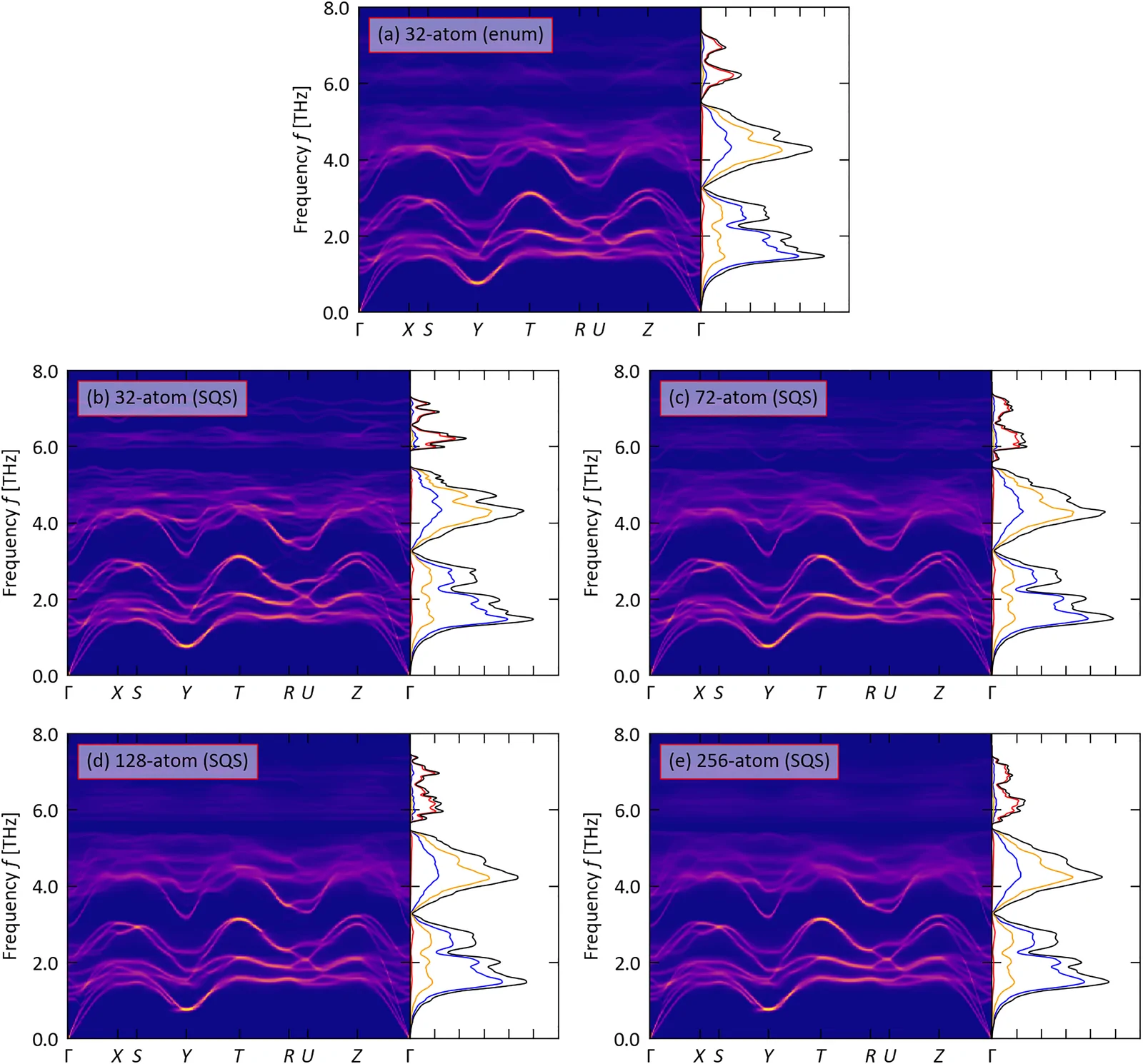
Unfolded dispersion and atom-projected density of states (PDoS) curves of the five Sn(S_0.2_Se_0.8_) models examined in this work. The dispersion and PDoS of the 32-atom supercell enumeration model are calculated for a formation temperature *T*_F_ = 900 K. The colour scale on the phonon dispersion plots shows the spectral weight and ranges from blue (small weight) to yellow (large weight). On each DoS plot the total DoS is shown in black and the eigenvector projections onto the S, Se and Sn atoms are shown in red, orange and blue respectively.

As found in previous work,^52^ a characteristic effect of alloying at the chalcogen site is a “smearing” of the high-frequency phonon bands associated with motion of the chalcogen sublattice. This is clearly seen in the (averaged) unfolded dispersion of the 32-atom supercell enumeration model, whereas the dispersion of the equivalent single 32-atom SQS model shows more pronounced fine structure in this part of the spectrum. The 72-atom SQS model partially captures the smearing but retains some discernible fine structure, while the larger 128- and 256-atom SQS show strong smearing of the high-frequency bands similar to the supercell-enumeration model. Similar contrasts can also be seen through the presence or absence of sharp features in the (P)DoS curves of the five models. Most notably, while the larger SQS cells appear to effectively converge the shape of the low- and mid-frequency features in the DoS, attributable to Sn and Se respectively, they retain some fine structure in the high-frequency feature from the S-based modes that is averaged out in the supercell enumeration model. We attribute this to the fact that there are comparatively few S atoms in our chosen composition with *x* ≈ 0.8, which means a single small supercell model cannot capture the range of S environments in the alloy.

The lattice thermal conductivity ***κ***_latt_ arises from two fundamental mechanisms, *viz.* “particle-like” transport ***κ***_p_ and a “wave-like” transport ***κ***_w_.^62^ The ***κ***_p_ is sensitive to the phonon group velocities ***ν***_***q****j*_ and is strongly suppressed by the symmetry breaking induced by alloying. On the other hand, the ***κ***_w_ depends inversely on the interband frequency spacing and is enhanced in the alloys due to the band smearing. We have previously determined that these two mechanisms can partially compensate for one another, and both must in principle be considered to obtain an accurate prediction of the ***κ***_latt_.^53^

The predicted scalar-average *κ*_latt_ for the five alloy models examined in this work, computed using the equivalent form of eqn (24), are compared to previous calculations on SnS and SnSe^52^ in Fig. 3. There is a substantial reduction in the *κ*_latt_ of all five alloys with respect to both endpoints, with a particularly pronounced effect at lower temperature. We obtain room-temperature (*T* = 300 K) *κ*_latt_ = 0.75–0.94 W m^−1^ K^−1^ for the alloy models, which are significantly smaller than the *κ*_latt_ = 2.28 and 1.66 W m^−1^ K^−1^ calculated for SnS and SnSe. These values are reduced to *κ*_latt_ = 0.47–0.56 W m^−1^ K^−1^ at *T* = 700 K, and also represent a favourable reduction compared to the endpoints (1.10/0.80 W m^−1^ K^−1^). We note that the room-temperature *κ*_latt_ for the 32-atom supercell enumeration model is larger than that obtained in our previous work,^52^ which is primarily because we did not include the *κ*_w_ in those calculations.

**Fig. 3:**
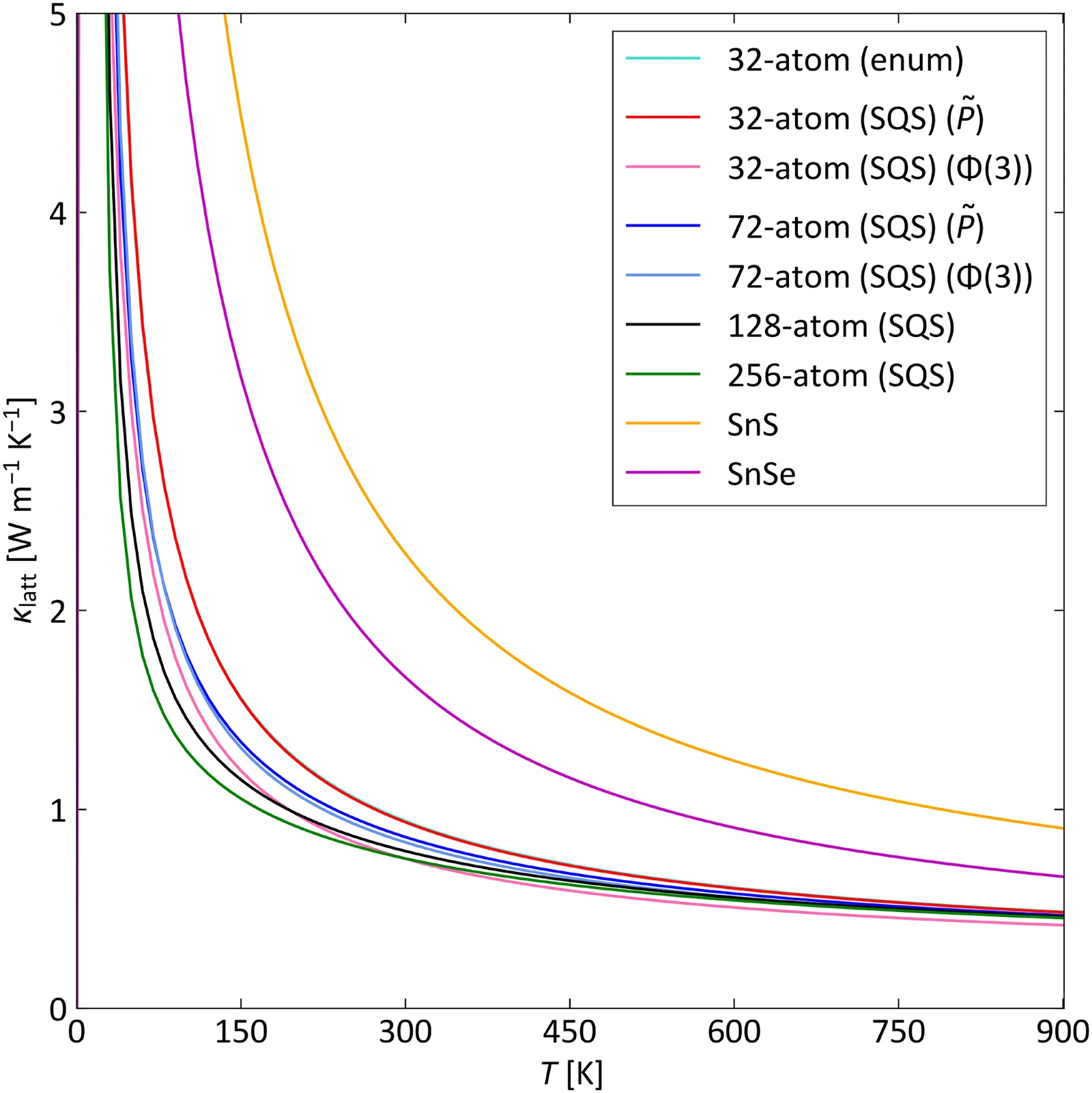
Scalar-average total lattice thermal conductivity *κ*_latt_ = *κ*_p_ + *κ*_w_, including both the “particle-like” and “wave-like” contributions *κ*_p_/*κ*_w_, of the five Sn(S_0.2_Se_0.8_) models examined in this work. Data for SnS and SnSe from our previous studies^42,44,52^ are also shown for comparison.

The “pseudo 2D” structure of orthorhombic SnS and SnSe leads to strong anisotropy in the ***κ***_latt_, with larger in-plane conductivity along the crystallographic *a* and *c* directions and much smaller conductivity along the layering *b* direction. In consolidated powders, the materials processing often results in a strong preferred crystallite orientation with the layering and in-plane directions respectively parallel and perpendicular to the pressing direction (‖/⊥). For this type of sample, the more realistic comparison with experimental measurements are:

27 *κ*^‖^_latt_ = *κ*^*yy*^_latt_

28
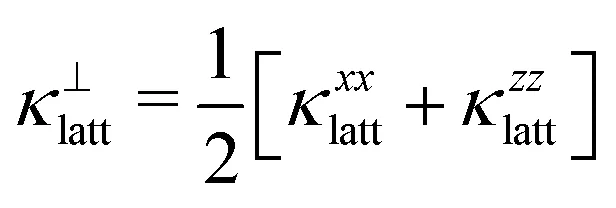


Ref. 34 reports a value of *κ*^‖^_latt_ = 0.52 W m^−1^ K^−1^ for Sn(S_0.2_Se_0.8_) at 323 K, which is within the range of our predictions of 0.46–0.56 W m^−1^ K^−1^ at *T* = 320 K. On the other hand, ref. 33 quotes a *κ*^‖^_latt_ of 0.11 W m^−1^ K^−1^ for (Ag_0.01_Sn_0.99_)(S_0.15_Se_0.85_) at *T* = 825 K, which is substantially lower than our calculated 0.30–0.34 W m^−1^ K^−1^ at a comparable *T* = 830 K. This discrepancy may be attributable to a number of factors, including a finite grain size and/or defects in the experimental samples, or to our model failing to capture the high-temperature anharmonicity in these materials.^13,28^ Nonetheless, the *κ*_latt_ calculated for the alloys are lower than the *κ*_latt_ = 0.47 we predict for bulk SnSe, and our calculations therefore successfully capture the qualitative effect of alloying. A comparison of the *κ*^‖^_latt_ and *κ*^⊥^_latt_ to the scalar-averaged *κ*_latt_ computed using eqn (24) is provided in Fig. S6.

The ***κ***_latt_ of the 32-atom supercell enumeration model and the larger 72-, 128- and 256-atom models were computed using the approach in ref. 52, where the three-phonon interaction strengths Φ_***q****j*,***q***′*j*′,***q***″*j*″_ are set to a constant value *P̃* estimated from the endpoint structures.^52^ The *P̃* are determined from a linear fit of the *κ*_latt_ at a representative temperature, and are typically temperature-independent or weakly *T*-dependent above ∼300 K.^52,53,84^ This procedure allows the predicted *κ*_latt_ to take into account differences in the harmonic modal properties and spectra, the latter of which affects the phonon lifetimes *τ*_***q****j*_ through conservation of energy selection rules, but avoids the intractable cost of computing the third-order force constants **Φ**(3) for large supercells.

For the smaller 32- and 72-atom SQS models it was also feasible to compute the **Φ**(3), albeit using a range cutoff of 4.5 Å for the larger model, and to calculate the Φ explicitly, allowing us to directly validate this approach. The *P̃* estimated for these models by linear interpolation between the endpoints are 1.47 × 10^−12^ eV^2^ and 2.93 × 10^−13^ eV^2^, respectively, which are reasonably close to the values of 2.11 × 10^−12^ eV^2^ and 3.10 × 10^−13^ eV^2^ determined by fitting the *κ*_latt_ obtained from the explicit calculations. The values of the *κ*_latt_ at 300 K (*i.e.*, the fitting temperature) obtained for the two models using the two sets of *P̃* are 0.75/0.94 and 0.83/0.86 W m^−1^ K^−1^ respectively, and the estimated values are both within 10% of the explicit calculations. Given the typical variability in experimental measurements, we consider this level of accuracy more than acceptable, and we therefore consider our method to be a viable approach to estimating the *κ*_latt_ of alloy models in cases where it is not feasible to compute the **Φ**(3).

Finally, to understand the impact of alloying on the *κ*_latt_ of the alloys, we compare the contributions from the *κ*_p_ and *κ*_w_ (Fig. 4(a)/(b)). The *κ*_p_ systematically decreases as the model size increases from 32 to 256 atoms. On the other hand, the *κ*_w_ shows the opposite behaviour, with the result that the overall *κ*_latt_ of all five alloy models are similar. The behaviour of the *κ*_p_ can be understood using the analysis developed in our previous work.^9^ We write the *κ*_p_ as the product of a harmonic function *κ*_p_/*τ*_ph_ and a weighted-average lifetime *τ*_ph_ according to:29
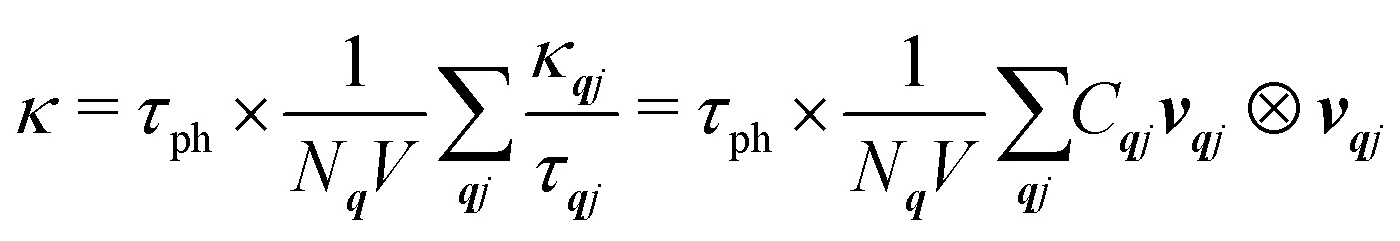


**Fig. 4:**
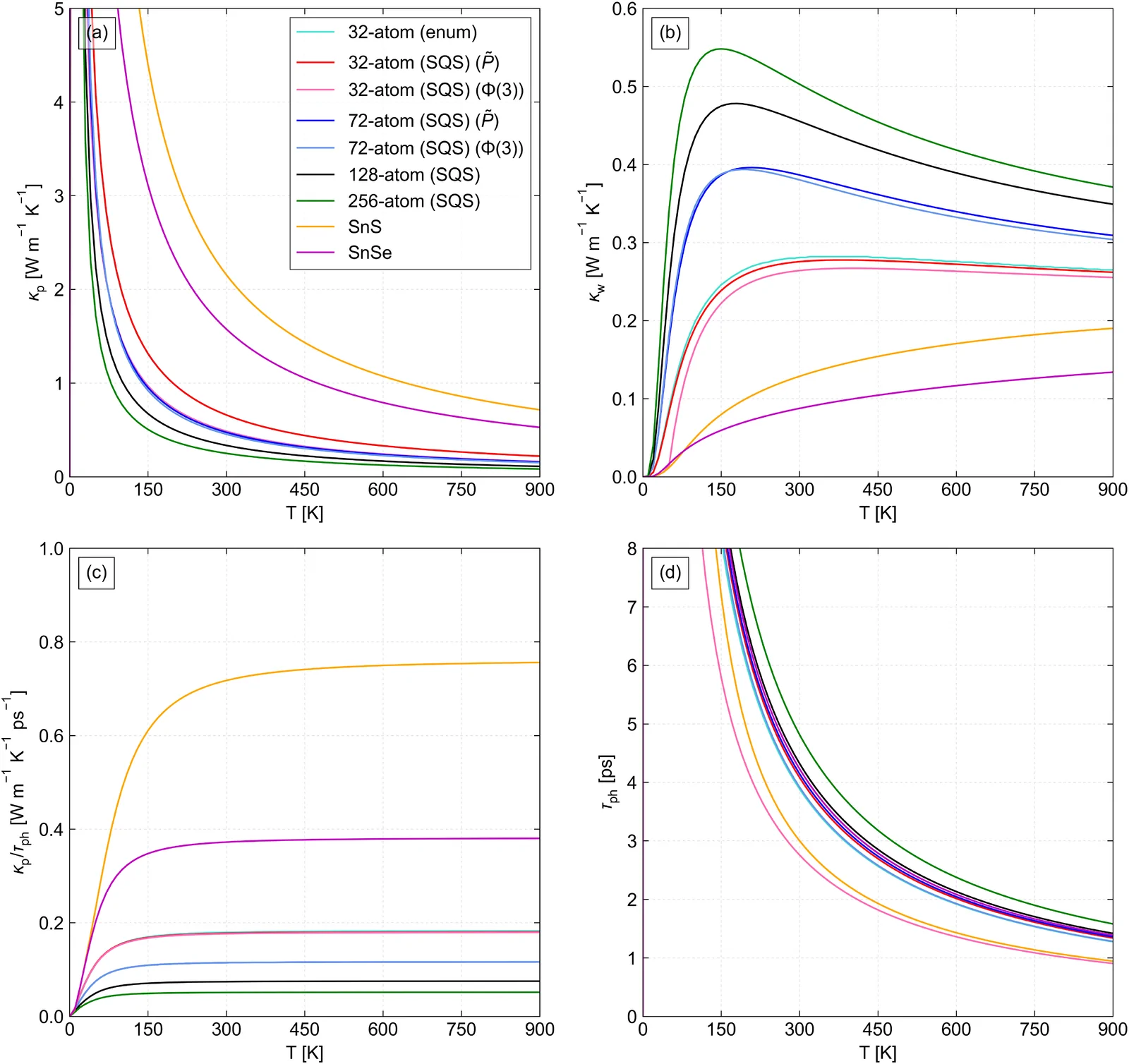
Analysis of the scalar-average thermal conductivity *κ*_latt_ of the five Sn(S_0.2_Se_0.8_) models examined in this work. Plots (a) and (b) show the contributions to the *κ*_latt_ from “particle-like” transport *κ*_p_ (a) and “wave-like” transport *κ*_w_ (b). Plots (c) and (d) show the decomposition of the *κ*_p_ into a harmonic term *κ*_p_/*τ*_ph_ (c) and a weighted-average phonon lifetime *τ*_ph_ (d) as described in eqn (8) in the text.^9^ For the 32- and 72-atom special quasi-random structure (SQS) models we show analyses of calculations performed with explicit third-order force constants **Φ**(3) and using estimated average three-phonon interaction strengths *P̃*. Data for SnS and SnSe from our previous studies^42,44,52^ are shown for comparison.

The *κ*_p_/*τ*_ph_ predominantly reflect differences in the phonon group velocities, and show a systematic decrease with model size that mirrors the behaviour of the *κ*_p_ (Fig. 4(c)). On the other hand, the majority of the *τ*_ph_ span a narrow range, with those of the 32-atom SQS model with explicit **Φ**(3) and the 256-atom SQS model with estimated *P̃* being notable outliers (Fig. 4(d)). This indicates that the reduction in *κ*_p_ is primarily due to lower phonon group velocities. For the pure chalcogenides this quantity shows a strong inverse correlation with unit-cell size,^9^ attributed to the less regular bonding environment in more structurally-complex materials, so this result is not surprising. On the other hand, the reduction in artificial/imposed periodicity results in a smaller Brillouin zone and substantial band folding, narrowing the interband spacing and increasing the *κ*_w_. Our calculations therefore suggest that increasing the level of disorder in alloys lowers the phonon velocities and the particle-like thermal conductivity, but that this is compensated by an increase in wave-like transport. This mirrors the finding in our previous work on Ag-doped SnS,^53^ where the same behaviour reproduced the expected convergence of the *κ*_latt_ towards the bulk limit when the dopant was diluted by using progressively larger simulation cells, and underscores the importance of accounting for this contribution in calculations on alloys.

### 3.2 Electronic transport properties

The calculated band gaps *E*_g_ of the five alloy models range from 0.63 eV for the smaller models to 0.87 eV for the larger models (Table S3), with near-quantitative agreement between the values for the 32-atom supercell enumeration and SQS models (*E*_g_ = 0.63/0.64 eV). The calculated bandgaps are all lower than the value of *E*_g_ = 1.06 ± 0.15 eV measured for SnS, but those of several of the models are also below the *E*_g_ = 0.86 eV reported for SnSe.^14,31^ While codes to perform electronic band unfolding are available,^85,86^ they are not easily integrated into the present workflow, and we were therefore unable to obtain an averaged dispersion for the 32-atom supercell enumeration model or unfolded dispersions of the four SQS models. By analysing the “folded” dispersions of the SQS models, it is not possible to determine whether an apparent direct bandgap would remain so in the unfolded band structure, but it is possible to unambiguously assign an indirect gap. In this context, the folded band structures all indicate indirect bandgaps, matching the SnS and SnSe endpoints (Fig. S7–S10).^87,88^

The fact that we predict the smaller alloy models to possess significantly lower *E*_g_ than SnSe is unusual and differs qualitatively from our previous calculations on Sn(S_1−*x*_Se_*x*_).^50^ The *E*_g_ of the *N* = 23 structures in the 32-atom supercell enumeration model range from 0.61–0.68 eV, and the *E*_g_ of the single 32-atom SQS structure is a representative average. We would expect HSE06 to be more accurate than the meta-GGA functional used in our previous study, and indeed we recently applied the supercell-enumeration approach in the present work to model the electronic structure of Ge(S_0.5_Se_0.5_) and obtained very good agreement with experimental measurements.^51^ With this in mind, our results clearly show that the unexpectedly low *E*_g_ is a property of the supercell size and not an anomaly associated with the (single) SQS structure. Moreover, since the supercell enumeration covers all the independent configurations in the 32-atom supercell, we can infer that the convergence to the more reasonable *E*_g_ of the larger supercells is likely due to the better representation of the medium-/long-range order in these models.

As outlined above, our procedure for computing the electrical properties involves a closed-form solution to the electron Boltzmann transport equations using approximate models for the electron scattering rates (carrier lifetimes).^38,39^ In principle, this can be straightforwardly applied to both the supercell-enumeration and SQS approaches for modelling alloys, since, unlike for the third-order force constants **Φ**(3), calculating the required uniform band structure and physical properties remains tractable even for large supercells. There are, however, two potential caveats. The first is that the transport modelling performs Brillouin zone interpolation and thus requires sufficiently dense ***k***-point sampling in the uniform band structure calculation. While this can be computationally intensive for large cells, our approach of computing the dense band structure with semi-local DFT and correcting the bandgap to a more accurate value computed using a hybrid functional on a smaller ***k***-point mesh largely mitigates this, particularly when used in conjunction with our non-self-consistent method.^67^ A second, potentially more important consideration, as alluded to above, is that the transport model is fundamentally a particle-like formulation (*cf.*eqn (7) and (16)). It is therefore likely that the electron group velocities ***v***_***k****j*_ and the derived properties could show a similar systematic supercell size dependence to the phonon velocities ***v***_***q****j*_. However, whereas this can be accounted for in the calculation of the *κ*_latt_ using the Wigner formulation, we are not aware of a widely-available equivalent for electronic transport. We explore this second point in detail in the following discussion.

Fig. 5 shows the predicted scalar-average p-type electrical conductivity *σ*, Seebeck coefficient *S*, power factor *S*^2^*σ* and electronic thermal conductivity *κ*_el_ of the five alloy models, again computed using eqn (24), as a function of *n* = 10^16^–10^20^ cm^−3^ at a fixed *T* = 700 K chosen to be below the *Pnma* → *Cmcm* phase transition of the endpoints (*T* = 878 and 807 K for SnS and SnSe respectively^27^). The electrical conductivity *σ* is conventionally expressed as:30
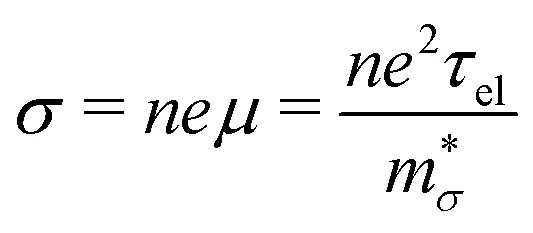
where 
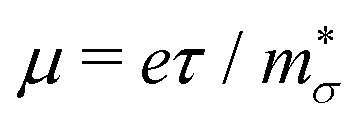
 is the carrier mobility, *τ*_el_ is a weighted-average electron relaxation time analogous to the *τ*_ph_ in eqn (29), and 
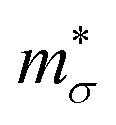
 is the conductivity effective mass. The linear dependence on *n* leads to the characteristic steep rise in *σ* with *n* when plotted on a log-linear scale as in Fig. 5(a). Interestingly, at larger *n* all five alloy models are predicted to have lower conductivity than the SnS and SnSe endpoints. Two other noteworthy observations are also apparent. Firstly, the averaged *σ* calculated for the 32-atom supercell enumeration model is very similar to that of the (single) 32-atom SQS model, which suggests that, as for the *E*_g_, the electrical properties are well modelled by a single configuration, at least for this small supercell size. Secondly, while the *σ* of both 32-atom models and the 128-atom SQS model are very similar, the 72- and 256-atom models are predicted to have substantially lower conductivity.

**Fig. 5:**
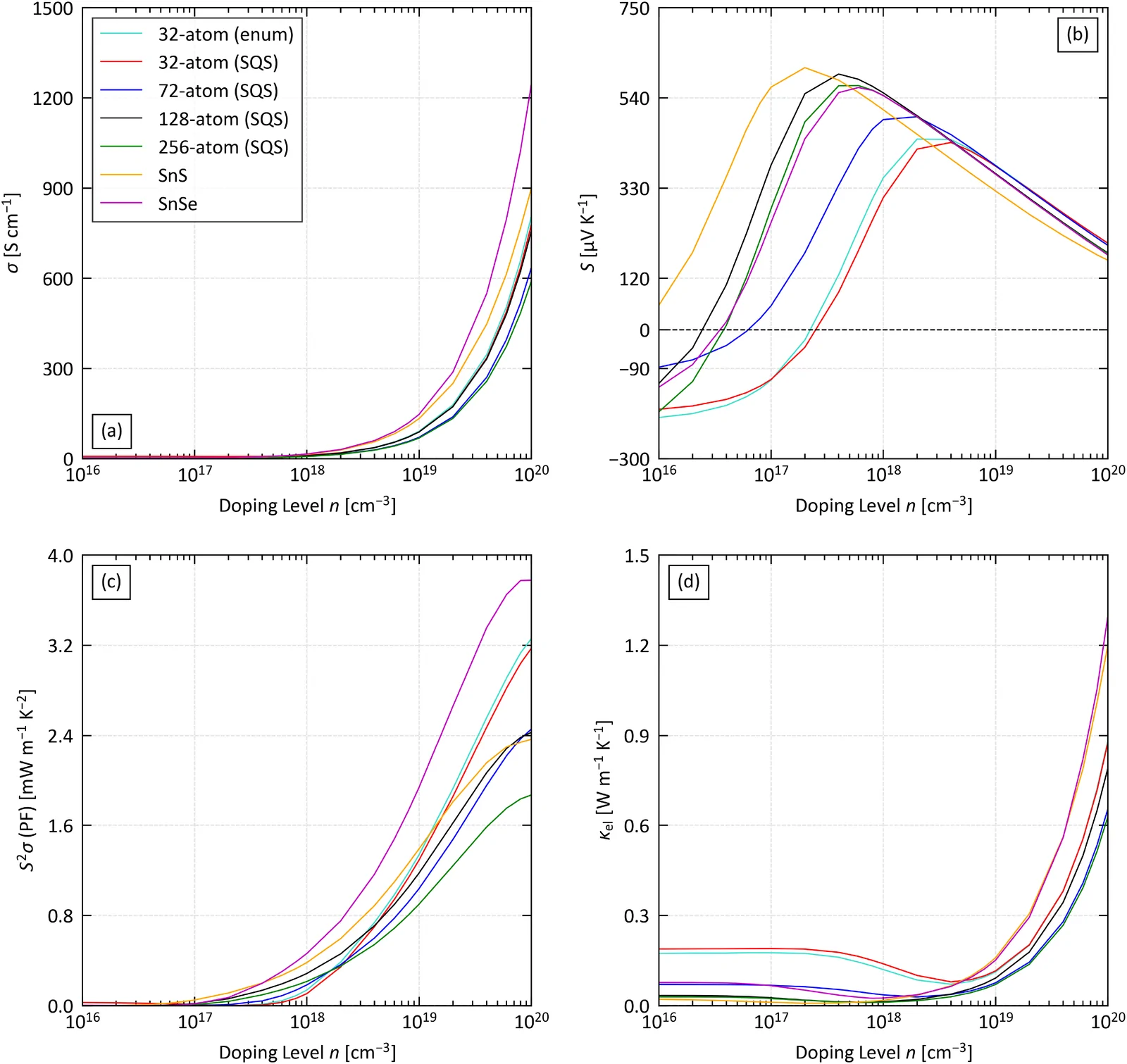
Predicted scalar-average electrical transport properties of the five Sn(S_0.2_Se_0.8_) models examined in this work as a function of extrinsic hole carrier concentration (“doping level”) *n* at a fixed *T* = 700 K: (a) electrical conductivity *σ*, (b) Seebeck coefficient *S*, (c) power factor *S*^2^*σ* (PF) and (d) electronic thermal conductivity *κ*_el_. Data for the SnS and SnSe endpoints taken from our previous study are shown for comparison.^42^

To attempt to understand the differences in conductivity between the five Sn(S_0.2_Se_0.8_) models and the SnS/SnSe endpoints, we used the approach reported in ref. 89 to estimate the 
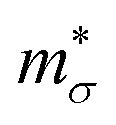
 and *τ*_el_ at a fixed *n* = 4 × 10^19^ cm^−3^ and *T* = 700 K (Table S4). The carrier masses of the alloy models show a general increase with supercell size, from 
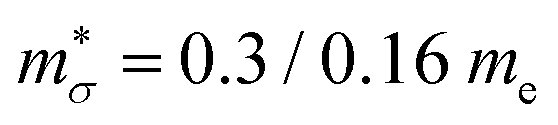
 for the two 32-atom models with p-/n-type doping to 
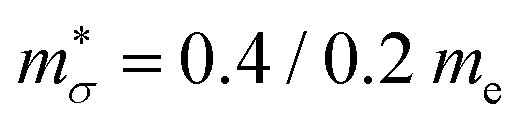
 for the 256-atom model. In all cases the p-type 
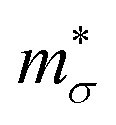
 are higher than those of SnS and SnSe, while the n-type 
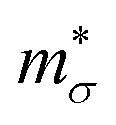
 of the smaller 32-atom models are between those of the two endpoints and closer to SnSe. Noting that the 
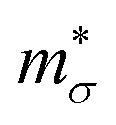
 are inversely proportional to the band curvature ∂^2^*ε*_***k****j*_/∂***k***^2^, the trend of reduced m* with model size is analogous to the behaviour of the phonon group velocities (*cf.*Fig. 4). However, the 72-atom model is something of an outlier, with p-type 
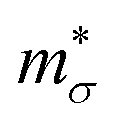
 close to the larger 128-atom model, rather than between the 32- and 128-atom models, and larger n-type 
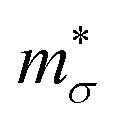
 than the 256-atom model. Inspection of the electronic band structures and DoS curves in Fig. S7–S10 shows no obvious differences in the spectra of the 72-atom model compared to those of the smaller 32-atom and larger 128/256-atom models beyond the expected band folding. One possible explanation for the anomalous behaviour of the 72-atom model is that the “odd” 3 × 1 × 3 supercell expansion is not commensurate with the boundary points in the *Pnma* Brillouin zone, whereas the other “even” expansions are, which could result in fundamentally different band folding. However, this explanation is purely speculative, and given that the valence-band maxima and conduction-band minima in these systems do not lie at the boundary points,^87,88^ a commensurate supercell expansion does not guarantee that these points align with any given Γ-centred ***k***-point mesh.

The *τ*_el_ range from 8.47–10.57 ps for p-type doping and 9.56–11.6 ps for n-type doping, compared to 10.16/13.36 and 8.75/12.44 ps for p- and n-doped SnS/SnSe. Unlike the 
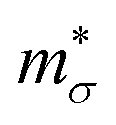
, however, there is no clear trend in the *τ*_el_. The longest carrier lifetimes are consistently predicted for the 128-atom SQS model, which combines with the trend in the effective masses to produce the differences in *σ* shown in Fig. 5(a). For the 32-atom models, the lower 
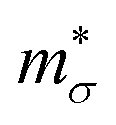
 and shorter *τ*_el_ counteract one another to produce similar predicted conductivity, whereas for the 72- and 256-atom models the higher 
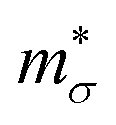
 and shorter *τ*_el_ combine to produce lower *σ*. To further investigate the variation in the *τ*_el_ we compared the energy- and momentum-dependent electron scattering rates Γ_***k****j*_ (*i.e.*, the inverse of the lifetimes *τ*_***k****j*_) as a function of energy for the p- and n-type SQS models at *T* = 700 K and a typical *n* = 4 × 10^19^ cm^−3^ (Fig. S11–S14). As in our previous work on GeS/GeSe and SnS/SnSe,^42–44^ we find that the *τ*_***k****j*_ of the electronic states close to the Fermi energy are dominated by polar-optic phonon (POP) scattering, with ionised impurity (IMP) scattering becoming increasingly significant away from the valence- and conduction-band edges. The alloys therefore show similar carrier scattering physics to the endpoints, and the differences in *τ*_el_ are mainly due to small changes in the POP scattering rates.

The Seebeck coefficient can be related to the *n* through the Mott equation:31
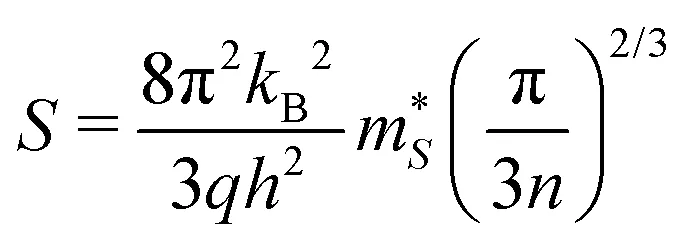
where *q* ± *e* for hole and electron carriers, respectively and 
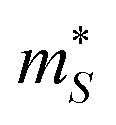
 is the Seebeck effective mass. The 
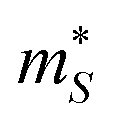
 is related to the 
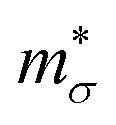
 through the valley degeneracy *N*_V_:32
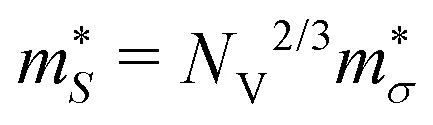


At low *n* the *S* of SnS, SnSe and of all five alloy models are predicted to be negative, which is indicative of dominant intrinsic n-type transport that requires a minimum hole doping level of up to ∼2 × 10^17^ cm^−1^ to suppress (Fig. 5(b)). The effect of this is that the *S* initially rise with *n* and peak between *n* ≈ 2 × 10^17^ to 5 × 10^18^ cm^−3^, then show the typical monotonic decrease at larger *n*. The minimum *n* required to obtain p-type conductivity, and the *n* where the peak *S* are obtained, show a strong correlation with the predicted bandgaps (Table S5). The shifts in the crossing points can be attributed to smaller bandgaps producing larger populations of thermally-generated charge carriers and raising the minimum hole doping level required to suppress the n-type transport. At larger *n* the *S* show a weaker dependence on the supercell size, with the five models predicted to show a narrow range of *S* = 248–272 µV K^−1^ at a typical “high” doping level of *n*= 4 × 10^19^ cm^−3^.^15,90–93^ Interestingly, the *S* of the alloy models are very similar to the *S* = 247 µV K^−1^ predicted for SnSe, and higher than the *S* = 219 µV K^−1^ of SnS, from which we conclude that the alloys retain the favourable larger Seebeck coefficient of the selenide under optimal doping.

The power factors *S*^2^*σ* reflect the differences in both the *S* and the *σ* (Fig. 5(c)). This results in an interesting scenario where the alloys are predicted to show lower PFs than both endpoints at small *n*, but the PFs of the two 32-atom models cross that of SnS at *n* ≈ 2 × 10^19^ cm^−1^, and those of the 72- and 128-atom SQS models cross at higher *n* ≈ 5 × 10^19^ cm^−3^. Given that the optimum p-type doping level for the endpoints typically falls within this range of *n*,^15,90–94^ our calculations are inconclusive on whether alloying should yield a PF between those of SnS and SnSe, or a PF comparable to SnS.

Finally, at large *n* the alloys and both the endpoints show degenerate semiconducting behaviour where the *κ*_el_ are proportional to the *σ* through the Wiedemann–Franz law:^7,95^

33 *κ*_el_ = *LσT*

where *L* is the Lorentz number and may in general depend on *n* and *T*.^7^ The *κ*_el_ therefore closely mirrors the trends in the *σ*. At *n* = 4 × 10^19^ cm^−3^ we predict *κ*_el_ = 0.56 W m^−1^ K^−1^ for SnSe, 0.34–0.38 W m^−1^ K^−1^ for the 32- and 128-atom alloy models, 0.27 W m^−1^ K^−1^ for the 256-atom model, and 0.56 W m^−1^ K^−1^ for SnS. This means the *κ*_el_ of the alloy models are predicted to be between 57–68% of the predicted *κ*_latt_ at *T* = 700 K (*cf.*Fig. 3). It is also noteworthy that the *κ*_el_ show an unusual fall around the *n* where the *S* change sign, and that the two 32-atom models are predicted to show a significant *κ*_el_ ≈ 0.18 W m^−1^ K^−1^ at low *n* due to the narrow bandgaps and resulting high thermally-generated electron concentrations.

As for the *κ*_latt_, there are a handful of experimental measurements of the electronic properties of Sn(S_0.2_Se_0.8_),^33–35^ and it is more appropriate to compute and compare properties along the out-of-plane (parallel) and in-plane (perpendicular) directions (*cf.*Eqn (27) and (28)). Ref. 33 reports room-temperature values of *S*^‖^ = 312 µV K^−1^ and *σ*^‖^ ≈ 10 S cm^−1^ for p-type (Ag_0.01_Sn_0.99_)(S_0.20_Se_0.80_) with *n* = 6.22 × 10^18^ cm^−3^. At a comparable *n* = 6 × 10^18^ cm^−3^ we predict values of *S*^‖^ = 281–316 µV K^−1^ and *σ*^‖^ = 41–66 S cm^−1^ across our five alloy models. Ref. 34 reports a p-type *σ*^‖^ ≈ 0.5 S cm^−1^ at 323 K for Sn(S_0.2_Se_0.8_) with a lower *n* ≈ 2.5 × 10^17^ cm^−3^, and we again predict comparable *σ*^‖^ = 1.4–2.3 S cm^−1^ at 320 K and *n* = 2 × 10^17^ cm^−3^. The Sn(S_0.2_Se_0.8_) samples prepared in ref. 35 have a similar *n* = 2.1 × 10^17^ cm^−3^ and measured p-type *S*^⊥^ = 265/*S*^‖^ = 279 µV K^−1^ and *σ*^⊥^ = 2.8 × 10^−3^/*σ*^‖^ = 1.53 × 10^−3^ at 323 K.^35^ These Seebeck coefficients are a more interesting point of comparison to the calculations because the low *n* falls in the region of carrier concentrations where the predicted *S* of the alloy models vary significantly (Fig. 5(b)). The closest *S*^⊥^ = 567 and *S*^‖^ = 575 µV K^−1^ are obtained for the 128-atom SQS model, but even these are large overestimates. Given that the peaks in the predicted Seebeck coefficients occur between the *n* reported in ref. 33 and 35, we attribute the poor reproduction of the latter results to the sensitivity of the *S* to the bandgap at lower *n*. On the other hand, the calculated *σ*^⊥^ = 6.06–8.69 and *σ*^‖^ = 1.42–2.27 S cm^−1^ predicted by the alloy models are orders of magnitude larger than the measurements, and the measurements are also inconsistent with those of ref. 34 despite the similar *n*. This strongly suggests the experimental samples in ref. 35 contain a high degree of defects, and since defects can modulate both the *S* and *σ*^96,97^ we are reluctant to read too much into the comparison with these measurements. Nonetheless, given the clear sensitivity of the Seebeck coefficient and power factor at lower *n* to the *E*_g_, it is reasonable to suggest that the predictions are likely to be more reliable above the peak *S*, *i.e.*, *n* ≥ 10^18^ cm^−3^ for the 128- and 256-atom models, and *n* ≥ 10^19^ cm^−3^ for the 32- and 72-atom models. Given that the optimal doping level for the Sn chalcogenides is on the order of 4 × 10^19^ cm^−3^,^42,43^ this does not impact the utility of our approach for predicting (optimum) thermoelectric performance.

Overall, our results suggest that alloying is likely to result in higher carrier effective masses and shorter carrier lifetimes compared to SnSe, which together lower the predicted conductivity and, as a consequence, the power factor *S*^2^*σ*. From a methodological standpoint, the reduction in carrier effective masses with supercell size is also an important observation. Given the clear crossover from dominant particle-like to wave-like transport seen in the analysis of the *κ*_latt_ (*cf.*Fig. 4), and the fact that typical approaches to predicting the electrical properties of bulk thermoelectric materials almost always rely on a particle-like description of the transport, it is likely that calculations on both small and large supercells will show significant size artifacts: small supercells may be unable to capture the range of local atomic environments in the real alloy and/or may impose an artificial medium- or long-range order, whereas large cells may predict anomalous electrical properties due to an equivalent of the Wigner formulation of phonon transport not being included in typical transport models.

While we have focused our analysis and discussion on the more usual p-type doping,^15,90,91^ both SnS and SnSe can also be n-type doped up to similar *n* ≈ 5 × 10^19^ cm^−3^,^92,93^ and we therefore also performed calculations for the n-type alloys (Fig. S15). Notably, the n-type *σ* of the alloys shows a clearer systematic trend in supercell size. The two 32-atom models have very similar and slightly larger *σ* than the 128-atom SQS models, with the predicted conductivities of all three models falling between the SnS and SnSe endpoints, whereas the larger 256-atom SQS model is predicted to have a much lower *σ* comparable to SnS. As for the p-type *S*, the n-type Seebeck coefficients are predicted to reach peak (minimum) values between *n* ≈ 10^17^–10^18^ cm^−3^, and the positions of the peaks again correlate to the bandgaps. However, given the intrinsic n-type behaviour evident from Fig. 5(b), the behaviour of the *S* under n-type doping must instead arise from the electronic states around the conduction-band edge (*i.e.*, higher *n* progressively involve additional states, with less favourable band curvature, in the transport). At large *n*, the *S* of the 32-, 128- and 256-atom alloy models are very similar, spanning a narrow range of |*S*| = 235–255 µV K^−1^ at *n* = 4 × 10^19^ cm^−3^, which is very similar to the n-type |*S*| = 230 and 227 predicted for SnS and SnSe. The 72-atom alloy model appears to be something of an outlier, with a lower *σ* and larger (more negative) *S*. Leaving this model aside, the clear contrast between the strong and weak dependence of the *σ* and *S* on the supercell size in the n-type alloys is noteworthy because the latter are calculated from the ratio of two moments of the transport distribution functions, and any systematic size effects in the ***ν***_***k****j*_ should largely cancel (*cf.*Eqn (16), (17), (20) and (21)). In keeping with this, the n-type PFs strongly reflect the behaviour of the *σ*, with the notable exception that the PF of the SnS endpoint appears to plateau at large *n* → 10^20^ cm^−3^. Finally, the *κ*_el_ closely follow the *σ*, as for the p-type systems, with the result that the predicted *κ*_el_ are 1.2–1.6× the *κ*_latt_ at *n* = 4 × 10^19^ cm^−3^ and *T* = 700 K. When taken with the weak size dependence of the *S*, this is noteworthy because it means that any systematic supercell size dependencies in the *σ* and *κ*_el_ might partially cancel in the predicted *zT*. We return to this point in the following subsection.

For completeness, we also compare the predicted *σ*, *S*, *S*^2^*σ* and *κ*_el_ of the five alloy models and the SnS and SnSe endpoints as a function of temperature at a fixed *n* = 4 × 10^19^ cm^−3^ in Fig. S16 and S17. At this high doping level the *σ* show a metallic-like decrease with temperature, confirming the degenerate semiconducting behaviour inferred from the *κ*_el_. On the other hand, the absolute *S* increase with temperature (*i.e.*, the p- and n-type *S* become more positive/negative), and the PFs show a shallow temperature dependence with a broad peak from 300–600 K. For the electronic thermal conductivity the sharp drop in the *σ* is partially counteracted by the temperature term in eqn (33), and the *κ*_el_ show a small, close to linear, decrease of 22–57% between 300–900 K.

Finally, and again for completeness, we also compare the predicted in-plane and out-of-plane electrical transport properties of the p- and n-type 32-atom supercell enumeration and 128-atom SQS models, both as a function of *n* at fixed *T* and *vice versa*, to the corresponding scalar averages in Fig. S18–S21. As found in our previous studies on SnS and SnSe,^42,43^ the p- and n-type alloys are both predicted to have relatively isotropic *S*, but higher *σ*, *S*^2^*σ* and *κ*_el_ along the in-plane and out-of-plane directions respectively. This means that for n-type doping the “easy” axis for electronic transport aligns with the “hard” axis for thermal transport, which is beneficial for a higher *zT*.^43^ We also note that our qualitative prediction of a higher *σ* and PF along the in-plane (⊥) direction in the p-type alloys is consistent with the experimental measurements in ref. 35, despite the fact that our predicted values are not a quantitative match.

### 3.3 Thermoelectric figure of merit

By combining the scalar-average electrical properties and lattice thermal conductivity, we obtain a predicted scalar-average figure of merit *zT* for each of the five alloy models as a function of doping level and temperature (Fig. 6 and S22). From these, we then determine the maximum *zT* (*zT*_max_) of the alloy models at temperatures up to the reported phase transition temperatures of the endpoints,^27^ which are collected together with the constituent properties in Table 3. We also include data for SnS and SnSe from our previous work,^42,43^ including the wave-like contribution to the lattice thermal conductivity for consistency with the calculations on the alloys.^44^

**Fig. 6:**
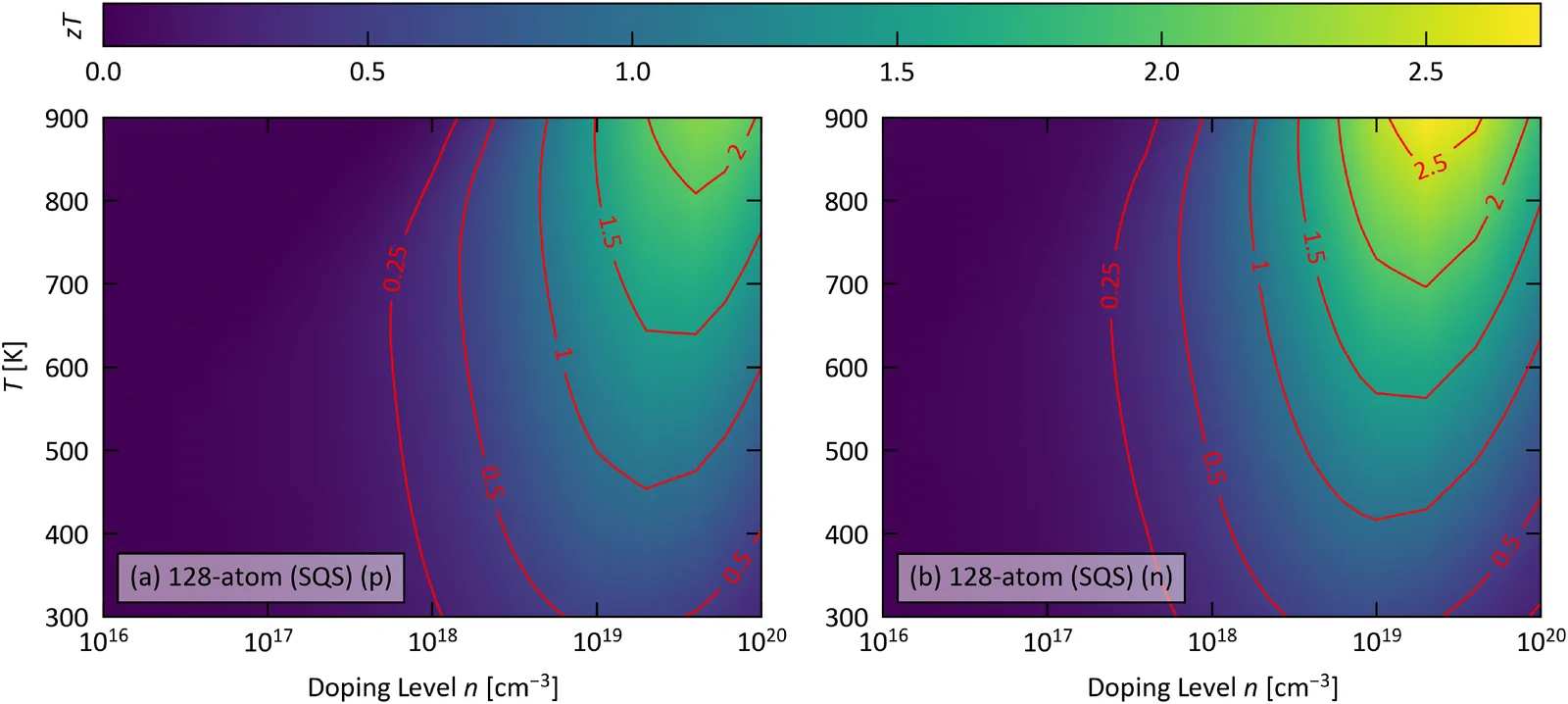
Predicted scalar-average thermoelectric figure of merit *zT* of p-type (a) and n-type Sn(S_0.2_Se_0.8_) (b) modelled using the 128-atom special quasi-random structure (SQS) model, as a function of extrinsic electron carrier concentration (“doping level”) *n* and temperature *T*. Values of *zT* = 0.25, 0.5, 1, 1.5, 2, and 2.5 are shown by contour lines.

**Table 3:** Predicted maximum scalar-average thermoelectric figure of merit *zT*_max_ of the five Sn(S_0.2_Se_0.8_) models examined in this work, with both p- and n-type doping. For each model we list the extrinsic carrier concentration (“doping level”) *n* and temperature at which the *zT*_max_ is attained, together with the associated electrical conductivity *σ*, Seebeck coefficient *S*, power factor *S*^2^*σ* (PF), and electronic, lattice and total thermal conductivity *κ*_el_, *κ*_latt_ and *κ*. The *κ*_latt_ of all five alloy models are computed using estimated average three-phonon interaction strengths *P̃*. Data for *Pnma* SnS and SnSe taken from our previous work^42,43^ are shown for comparison, and the *κ*_latt_ include the contribution from intraband tunnelling^44^

		*n* [cm^−3^]	*T* [K]	*zT*	*σ* [S cm^−1^]	*S* [μV K^−1^]	PF *S*^2^*σ* [mW m^−1^ K^−2^]	*κ* [W m^−1^ K^−1^]		
								*κ*_el_	*κ*_latt_	*κ*
p-Type	32-atom (enum)	4 × 10^19^	800	2.21	285	287	2.36	0.34	0.52	0.86
	32-atom (SQS)	4 × 10^19^	800	2.15	277	288	2.29	0.34	0.51	0.85
	72-atom (SQS)	4 × 10^19^	800	1.95	223	284	1.79	0.24	0.50	0.74
	128-atom (SQS)	4 × 10^19^	800	1.99	274	266	1.95	0.30	0.49	0.79
	256-atom (SQS)	4 × 10^19^	800	1.67	210	265	1.47	0.23	0.48	0.71
	SnS^42,43,52^	4 × 10^19^	880	1.26	305	255	1.98	0.46	0.92	1.38
	SnSe^42,43,52^	4 × 10^19^	800	2.03	448	262	3.07	0.49	0.72	1.21

n-Type	32-atom (enum)	2 × 10^19^	800	2.56	304	−314	3.00	0.42	0.52	0.94
	32-atom (SQS)	2 × 10^19^	800	2.53	299	−315	2.96	0.42	0.51	0.93
	72-atom (SQS)	4 × 10^19^	800	2.22	389	−272	2.88	0.54	0.50	1.04
	128-atom (SQS)	2 × 10^19^	800	2.40	282	−310	2.70	0.41	0.49	0.90
	256-atom (SQS)	2 × 10^19^	800	2.26	237	−308	2.25	0.32	0.48	0.80
	SnS^42,43,52^	4 × 10^19^	880	1.60	386	−262	2.65	0.54	0.92	1.46
	SnSe^42,43,52^	2 × 10^19^	800	2.38	400	−299	3.58	0.48	0.72	1.20

All five alloy models are predicted to achieve the highest *zT* at doping levels of 2–4 × 10^19^ cm^−3^ and the highest *T* = 800 K we tested, which matches predictions for SnS and SnSe.^42,43^ The higher *n* are required to optimise the *σ* and power factor *S*^2^*σ*, while higher *T* minimises the thermal conductivity and in particular the *κ*_latt_ (*cf.*Fig. 3 and 5). These doping levels are readily obtained in experiments on SnS and SnSe,^15,90,93,94,98,99^ with both p- and n-type carriers, and should therefore also be attainable in Sn(S_1−*x*_Se_*x*_) alloys.

The predicted *zT* of the alloy models shows a clear size dependence, with the two 32-atom models yielding comparable and larger *zT* and the 256-atom SQS model yielding a smaller *zT*. Across the five alloy models the p- and n-type *zT* vary by a factor of 1.3 and 1.1×, respectively, which is predominantly due to differences in the electrical properties. The p- and n-type PFs vary by 1.6/1.3×, but these are partially offset by similar 1.5/1.3× variation in the *κ*_el_, which accounts for 32–52% of the predicted (total) thermal conductivity. In contrast, the *κ*_latt_ shows a systematic but much smaller variation with system size, falling from 0.51/0.52 for the two 32-atom models to 0.48 for the 256-atom model. Given that we are confident our approach correctly accounts for the crossover from dominant particle-like to wave-like transport with increasing disorder, this small reduction may be a real consequence of the better representation of the medium- and long-range structure of the alloy in the larger cells. As discussed in the previous section, we attribute the systematically lower *σ* of the 256-atom SQS model to a size artifact. Bearing in mind the 2× larger *n* at which the n-type *zT*_max_ is obtained, the smaller 72-atom model is also predicted to show an anomalously low electrical conductivity, which, again as discussed above, appears to be a peculiarity of this supercell expansion. Based on these observations, and the representation of the disorder as inferred from the unfolded phonon band structures in Fig. 2, we consider the 32-atom supercell enumeration and 128-atom SQS models to strike the best balance of an adequate description of the disorder and minimising supercell-size artefacts in the predicted electrical properties, and so we focus our discussion on these models. We note, however, that the single 32-atom SQS model does seem to be a good proxy for the averaged electronic and thermal transport of the supercell enumeration model (Tables S6 and S7).

Both models predict that alloys with composition close to Sn(S_0.2_Se_0.8_) should show *zT* significantly higher than SnS and comparable to or higher than SnSe. With p-type doping, alloying reduces the *σ* but also the *κ*_el_, and, in combination with the reduced *κ*_latt_, leads to a predicted reduction in the total high-temperature *κ* of around 40% compared to SnS and 30% compared to SnSe. The 128-atom model is predicted to have a similar *S*^2^*σ* to SnS, whereas the 32-atom model is predicted to have a 20% higher PF that lies between those of SnS and SnSe and leads to a higher predicted *zT*. n-type SnS and SnSe are predicted to show superior performance to their p-type counterparts due to a higher *σ* (for a given *n*), a larger absolute |*S*|, and a higher PF.^42,43^ Compared to endpoints, the two alloy models are predicted to show lower *σ*, comparable or larger |*S*|, and lower total *κ*, which again result in comparable or slightly higher *zT* than SnSe. We note that, while experimental results suggest the n-type *n* required to optimise the *zT* are achievable,^92,93^ to the best of our knowledge the higher *zT* predicted in this and previous studies^42,43^ have yet to be realised. This suggests there could be additional materials engineering barriers to achieving high n-type *zT* that are not captured in our calculations.

Due to the strong anisotropy in the electronic and thermal transport, the *zT* of both endpoints and the alloy models also show significant anisotropy. We therefore calculated and compared the in-plane (⊥) and out-of-plane (‖) *zT* and *zT*_max_ with the scalar averages (Fig. S23/S24 and Table S8). In the p-type systems, the in-plane and out-of-plane directions represent the easy and hard axes for electrical and thermal transport, respectively, and the highest *zT* are obtained along the in-plane direction. In the n-type systems, the out-of-plane direction is also the easy axis for electrical transport, and the highest *zT* are obtained along the out-of-plane direction. Under the conditions listed in Table 3, the 32-atom supercell enumeration and 128-atom SQS models are predicted to have in-plane p-type *zT*^⊥^ = 2.28/2.18, which are slightly larger than the p-type *zT*^⊥^ = 2.09 for SnSe.^42^ Similarly, the two models are predicted to have out-of-plane n-type *zT*^‖^ = 4.61/4.35, compared to *zT*^‖^ = 4.25 for SnSe.^43^

While most experimental work on the Sn chalcogenides has focused on the “headline” high-temperature *zT*, the low-temperature *zT* are more relevant to many heat-recovery scenarios.^6,100^Fig. 7 compares the predicted *zT* as a function of temperature of SnS, SnSe and the 32-atom supercell enumeration and 128-atom SQS models of Sn(S_0.2_Se_0.8_) at a common *n* = 4 × 10^19^ cm^−3^. This analysis shows that the alloys have *zT* competitive with or higher than SnSe at high temperature, but we also predict modest increases of 10 and 17% in the p- and n-type *zT* at room temperature, and we predict that the p-type 32-atom supercell enumeration model would attain a benchmark *zT* = 1 at 450 K. We attribute the improved low-temperature performance to the significant reduction in the low-*T κ*_latt_, which is partially due to the shallower *T* dependence of the *κ*_w_ that dominates in more disordered systems.^44^ Our calculations therefore highlight alloying as a potential route to improving the low-temperature *zT*.

**Fig. 7:**
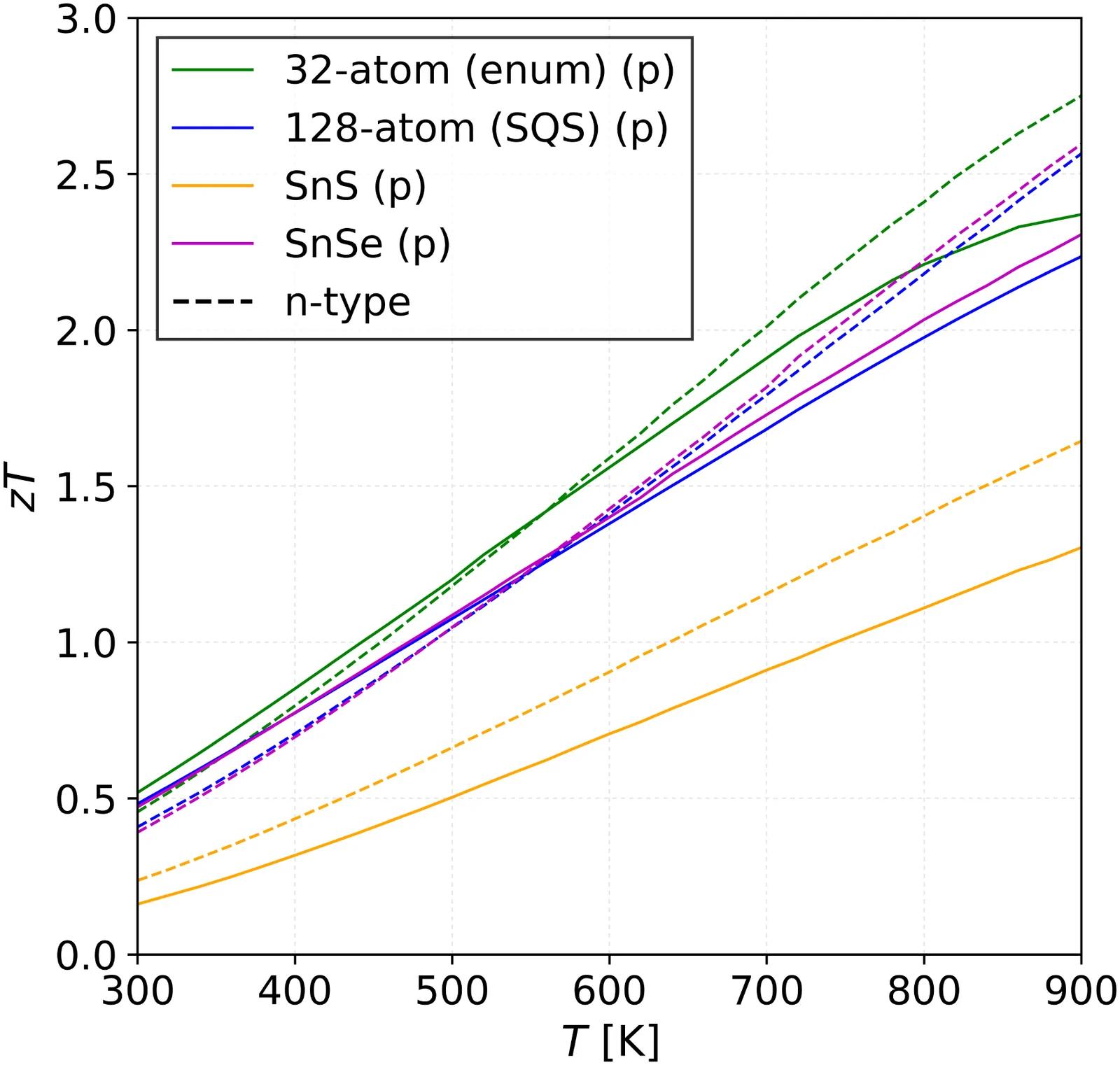
Predicted scalar-average thermoelectric figure of merit *zT* of p- and n-type SnS, SnSe and Sn(S_0.2_Se_0.8_) alloys as a function of temperature at a fixed extrinsic electron carrier concentration (“doping level”) *n* = 4 × 10^19^ cm^−3^. The data for *Pnma* SnS and SnSe are taken from our previous work^42,43^ and the *κ*_latt_ include the contribution from intraband tunelling.^44^

The measurements on (Ag_0.01_Sn_0.99_)(Se_0.80_S_0.20_) in ref. 33 report a p-type *zT*^‖^ = 1.67 at *n* = 6.22 × 10^18^ cm^−3^ and *T* = 823 K. This is significantly larger than our calculated *zT*^‖^ = 0.17/0.53 for the 32-atom supercell enumeration and 128-atom SQS models at a comparable *n* = 6 × 10^18^ cm^−3^ and *T* = 820 K. This appears to be largely due to our higher predicted *κ*^‖^ = 0.33/0.36 W m^−1^ K^−1^, which is more than a factor of two larger than the *κ*^‖^ = 0.15 W m^−1^ K^−1^ measured in the experiments.^33^ We note, however, that we predict comparable *zT* = 1.14/1.39 along the perpendicular direction. Ref. 34 measured values of *zT*^‖^ = 0.82 and *zT*^⊥^ = 0.62 for Sn(S_0.2_Se_0.8_) at *n* ≈ 2.5 × 10^17^ cm^−3^ and *T* = 820 K, which are approximately an order of magnitude larger than our predicted *zT*^‖^ = 0.07/0.09 and *zT*^⊥^ = 0.01/0.08 at comparable *n* = 2 × 10^17^ cm^−3^ and *T* = 820 K. In this case, the discrepancy arises from a combination of an 18–50% larger predicted *κ* and a 75–76% lower predicted *σ*. Our calculations using the 128-atom SQS model also underestimate the similar p-type *zT*^‖^ = 0.64 measured for the Sn(S_0.2_Se_0.8_) samples in ref. 35, but in this case because the predicted *S*^2^*σ* = 0.05 mW m^−1^ K^−2^ is approximately 6× lower than the measured PF. As noted above, however, the *n* ≈ 2 × 10^17^ cm^−3^ of the samples in ref. 34 and 35 are in the region where the Seebeck coefficients are highly sensitive to the electronic bandgap – indeed, our 32-atom supercell enumeration model is predicted to show intrinsic n-type behaviour at this *n* (*cf.*Fig. 5) – and thus where our predictions are likely to be least accurate. We would expect our predictions to be most accurate at the larger *n* that optimise the *zT*, although the comparison to ref. 33 suggests it might be prudent to explore the effect of crystallite size on the *κ*_latt_.^101^ More broadly, there are comparatively few experimental studies on the Sn(S_1−*x*_Se_*x*_) system, and the present calculations suggest the results could be improved if the carrier concentrations can be optimised to the same level as in flagship studies on SnSe.^14,15^

## 4 Discussion and conclusions

In this study, we have implemented and benchmarked a first-principles modelling workflow for predicting the electronic and thermal transport properties and thermoelectric figure of merit, *zT*, of alloys.

Using the characteristic “smearing” of the high-frequency chalcogen modes in the unfolded phonon dispersion as a metric, we find that a (single) 128-atom special quasi-random structure (SQS) model of Sn(S_0.2_Se_0.8_) gives comparable results to a thermodynamic average over a complete set of 23 symmetry-independent configurations in a 32-atom supercell. On this basis we recommend a “ballpark” size of ∼100 atoms as a good balance between computational cost and capturing the range of local atomic environments in the alloy.

Our approach of using averaged three-phonon interaction strengths interpolated from the endpoint structures^52^ appears to provide a quantitative estimate of the lattice thermal conductivity obtained by explicit computation of the third-order force constants. Crucially, this also allows both the particle- and wave-like contributions to the *κ*_latt_ to be evaluated, which captures the progressive change in character to amorphous-like thermal transport with the reduction of (imposed, artificial) periodicity in larger supercells.^53^ The approach of using semi-classical Boltzmann transport theory with approximate models for the electron relaxation times^38,39^ can be straightforwardly applied to alloys, but is based on the assumption of dominant particle-like transport, and this results in a different type of size artifact whereby large supercells predict anomalously low electrical conductivity. At the typical high doping levels that optimise the *zT*, this is in most cases likely to be partially mitigated by the similar impact on the electronic thermal conductivity, although we would recommend explicit tests for size dependencies by, for example, comparing against calculations on small supercells.

We highlight here another important limitation of our study, which also applies to similar calculations on bulk materials, *viz.* the lack of an explicit description of chemical doping. The large *n* ≈ 4 × 10^19^ cm^−3^ corresponds to doping levels on the order of a few at%, and the presence of dopants at such high concentrations could impact the electronic and thermal transport properties in ways that are not accounted for in our modelling approach. Atomic substitutions – and indeed other point defects such as vacancies – can act as phonon-scattering centres, and could further suppress the ***κ***_latt_ relative to the impact of the chalcogen disorder predicted in the our calculations. Heavy doping can also modulate the electronic band structure, leading to *e.g.* bandgap renormalisation, which can affect the transport properties, and these effects are not captured within the rigid-band approximation used in our calculations.^63,64^ While the former is more likely than not to have a beneficial impact on the thermoelectric performance, and would make our predicted ***κ***_latt_ an upper bound to experiments, the effects of the latter could be much more unpredictable. However, we have found that the RBA gives very reasonable predictions for bulk chalcogenide thermoelectrics with *n* up to 10^20^ cm^−3^,^42,43,101,102^ and we have no reason to suspect this would not be the case for alloys.

Despite these limitations, the present results suggest that the modelling approach presented here, with appropriate supercell sizes, is capable of realistic qualitative predictions of the effects of alloying on the thermoelectric performance. All our models predict the expected reduction in the *κ*_latt_ due to the alloying, as well as the reduced *σ* we would intuitively expect if alloying has a similar effect on both the electronic and phonon band dispersions. However, predictions of the Seebeck coefficient are more challenging due to the narrow bandgap, which means that small changes in the predicted *E*_g_ across different alloy models translate into large variations in the *S* at low doping levels. Despite this issue, our models predict that the highest *zT* are obtained under comparable conditions to the endpoints, and suggest a modest enhancement in the *zT* that qualitatively matches experimental findings. Another important qualitative finding is that the shift from predominantly particle-like *κ*_latt_ in the endpoints to wave-like transport in the alloys leads to a different temperature dependence that favours a reduced low-*T* thermal conductivity and higher low-*T zT*.^44^

On the other hand, the small number of experimental studies makes it somewhat difficult to assess the quantitative accuracy of our predictions. In particular, many of the Sn(S_1−*x*_Se_*x*_) thermoelectric materials reported to date appear to have been prepared with relatively low (unoptimised) *n*, and the considerable variation in the measured properties suggests preparation conditions may have an outsized impact. With this in mind, the fact that the base workflow has been shown to provide quantitative predictions of the *zT* of SnS/SnSe and related materials that have been more extensively characterised,^42,43,101,102^ and that the predictions we obtain for Sn(S_0.2_Se_0.8_) are consistent with the typical behaviour expected for alloys, we are confident that our approach should be able to make quantitative predictions of the *zT* of bulk alloys under optimal conditions.

Finally, taking the 128-atom SQS structure as a preferred model for the Sn(S_0.2_Se_0.8_) alloy, the complete set of calculations required a total of ∼68 000 CPU hours, which breaks down as approx. 2700 for the geometry optimisation, 35 300 for the harmonic phonon calculation to estimate the lattice thermal conductivity, and 30 000 to perform the electronic-structure calculations and to compute the additional materials properties required to estimate the Seebeck coefficient, electrical conductivity and electronic thermal conductivity. While the feasibility of this calculation clearly depends on available resources, given access to a modern high-performance computing system it should be computationally efficient enough to explore a range of compositions, *e.g.*, additional compositions along the Sn(S_1−*x*_Se_*x*_) binary line, the (Ge_1−*x*_Sn_*x*_)S and (Ge_1−*x*_Sn_*x*_)Se mixed-cation systems, or even quaternary (Ge_1−*x*_Sn_*x*_)(S_1−*y*_Se_*y*_) compositions. We plan to explore these possibilities in a future publication.

Future development of the methodology should focus on improving the description of the electrical transport to address the dependence on supercell size by accounting for both particle- and wave-like electrical transport in an analogous manner to the Wigner formulation of the *κ*_latt_.^62^ One possible approach is the non-equilibrium Green's function method, where a bias is applied to the model through virtual contacts (*e.g.*, ref. 103). Another option may be to calculate the *σ* from the DC (*E* = 0) component of the optical conductivity, and the Seebeck coefficient and *κ*_el_ from phenomenological relationships such as the Mott relation and the Wiedemann–Franz law.^95,104^ The latter has the advantage of simplicity, but with present implementations it would likely be difficult to include per-state electronic linewidths, and the approach would only be applicable under certain conditions (*e.g.*, the degenerate limit for the Wiedemann–Franz law).

## Author contributions

Conceptualisation – JMS; methodology – all authors; data curation – all authors; formal analysis and investigation – all authors; writing (original draft) – MZ and JMF; writing (review and editing) – all authors; funding acquisition – JMS. All authors have read and agreed to the published version of the paper.

## Conflicts of interest

There are no conflicts to declare.

## Supplementary Material

CP-OLF-D6CP02931K-s001

## Data Availability

Raw data from the calculations performed in this study will be made available to download free of charge following acceptance from an online repository at DOI: https://doi.org/10.17632/tsdyh5kk46. Supplementary information (SI): includes additional data to support the computational methodology and the discussion in the text. See DOI: https://doi.org/10.1039/d6cp02931k.
