# Supplementary material for: First-principles prediction of the thermoelectric properties of Sn(S0.2Se0.8) alloys
Source: Phys Chem Chem Phys. 2026 Oct 19. Online ahead of print. doi: 10.1039/d6cp02931k (PMC13718281; doi:10.1039/d6cp02931k)
Supplement: Supplementary file 1 [file CP-OLF-D6CP02931K-s001.pdf]

Supplementary Information (SI) for Physical Chemistry Chemical Physics.
This journal is © the Owner Societies

Electronic supporting information: First-principles study of the thermoelectric properties of $Sn(S_{0.2}Se_{0.8})$ alloys

Min Zhang, Joseph M. Flitcroft and Jonathan M. Skelton

1 Computational methods

Table S1 Comparison of the lattice parameters a, b and c, cell volume V, total energy E_0 and direct/indirect electronic band gaps $E_{g,dir}/E_{g,indir}$ of SnS and SnSe obtained using projector augmented-wave pseudopotentials with and without the semi-core Sn 4d electrons included in the valence region. The E_g are calculated using PBEsol+D3.

System	Sn Pseudopotential	a / Å	b / Å	c / Å	V Å^3	E_0 / eV	$E_{g,dir}$ / eV	$E_{g,indir}$ / eV
SnS	Sn (d)	4.22	10.99	3.96	183.4	-39.7	0.662	0.8
	Sn	4.20	10.99	3.96	182.64	-39.72	0.624	0.773
SnSe	Sn (d)	4.34	11.35	4.12	202.99	-37.43	0.439	0.564
	Sn	4.33	11.35	4.12	202.37	-37.44	0.406	0.534

Table S2 Electronic band gaps E_g and electron and hole effective masses m_e^* /m_h^* of the five Sn($S_{0.2}Se_{0.8}$) models examined in this work obtained using $\boldsymbol{k}$-point meshes with 2 and 3× more subdivisions along each reciprocal lattice vector than the "base" meshes used for geometry optimisation. All values are calculated using PBEsol+D3.

	$\boldsymbol{k}$-point density	E_g / eV	m_h^* / m_e	m_e^* / m_e
32-atom (SQS)	2×	0.366	-0.182, -0.063	0.037, 0.041
	3×	0.361	-0.132, -0.074	0.044, 0.054
72-atom (SQS)	2×	0.377	-0.383, -0.134	0.208, 0.301
	3×	0.372	-0.314, -0.097	0.174, 0.225
128-atom (SQS)	2×	0.394	-0.129, -0.083	0.561, 0.714
	3×	0.363	-0.146, -0.113	0.046, 0.192
256-atom (SQS)	2×	0.396	-0.168, -0.092	0.051, 0.146
	3×	0.367	-0.232, -0.106	0.042, 0.153

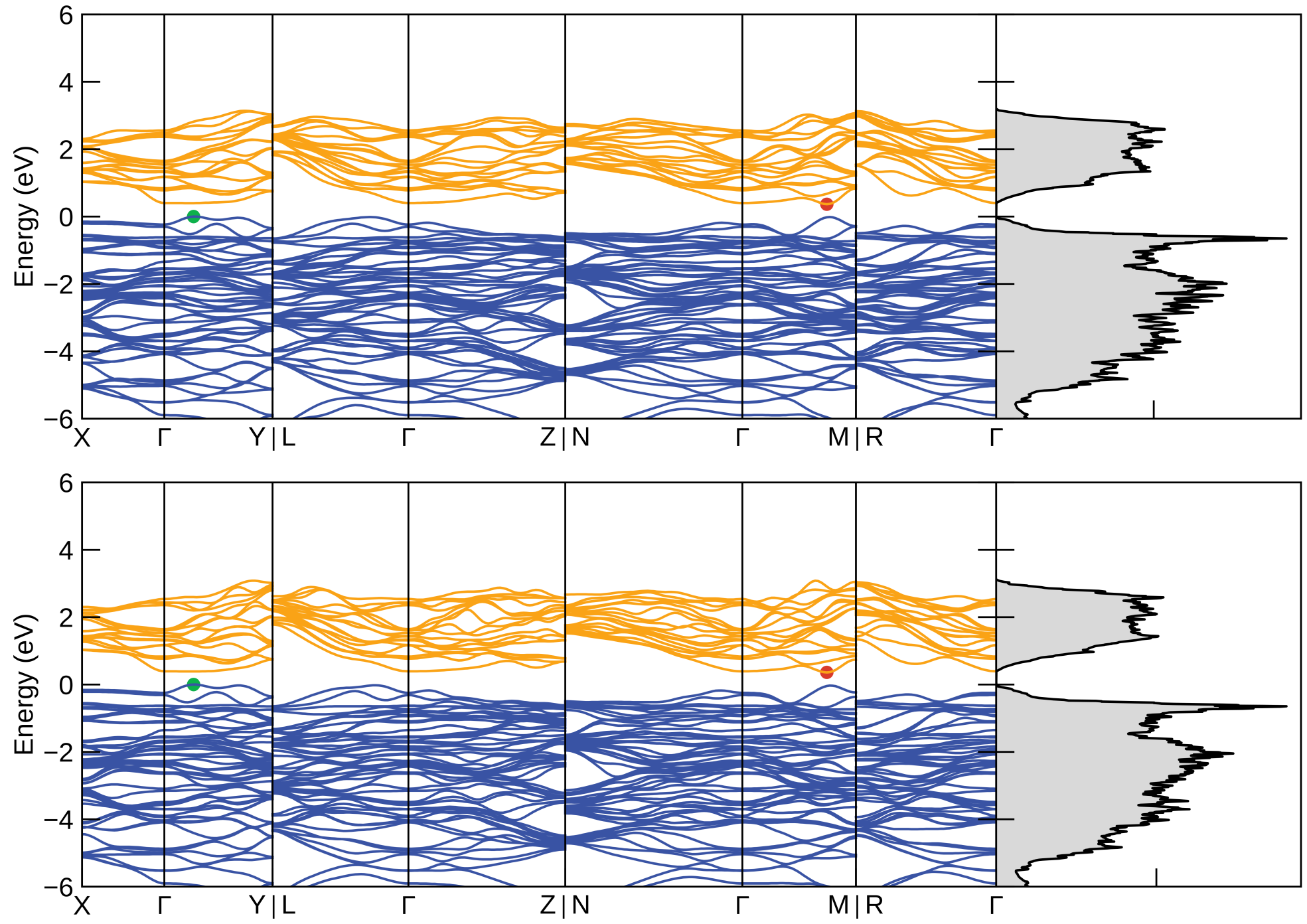

Fig. S1 Electronic band structure and density of states (DoS) of the 32-atom special quasi-random (SQS) model of $Sn(S_{0.2}Se_{0.8})$ obtained using PBEsol+D3 and $\boldsymbol{k}$-point meshes with 2 and $3\times$ more subdivisions along each reciprical lattice vector (top/bottom) than the "base" meshes used for geometry optimisation. In each band structure, the valence and conduction bands are shown in blue and orange, respectively, the valence-band minimum and conduction-band maximum (VBM/CBM) are marked with green and red solid circles, and the VBM is set to 0 eV.

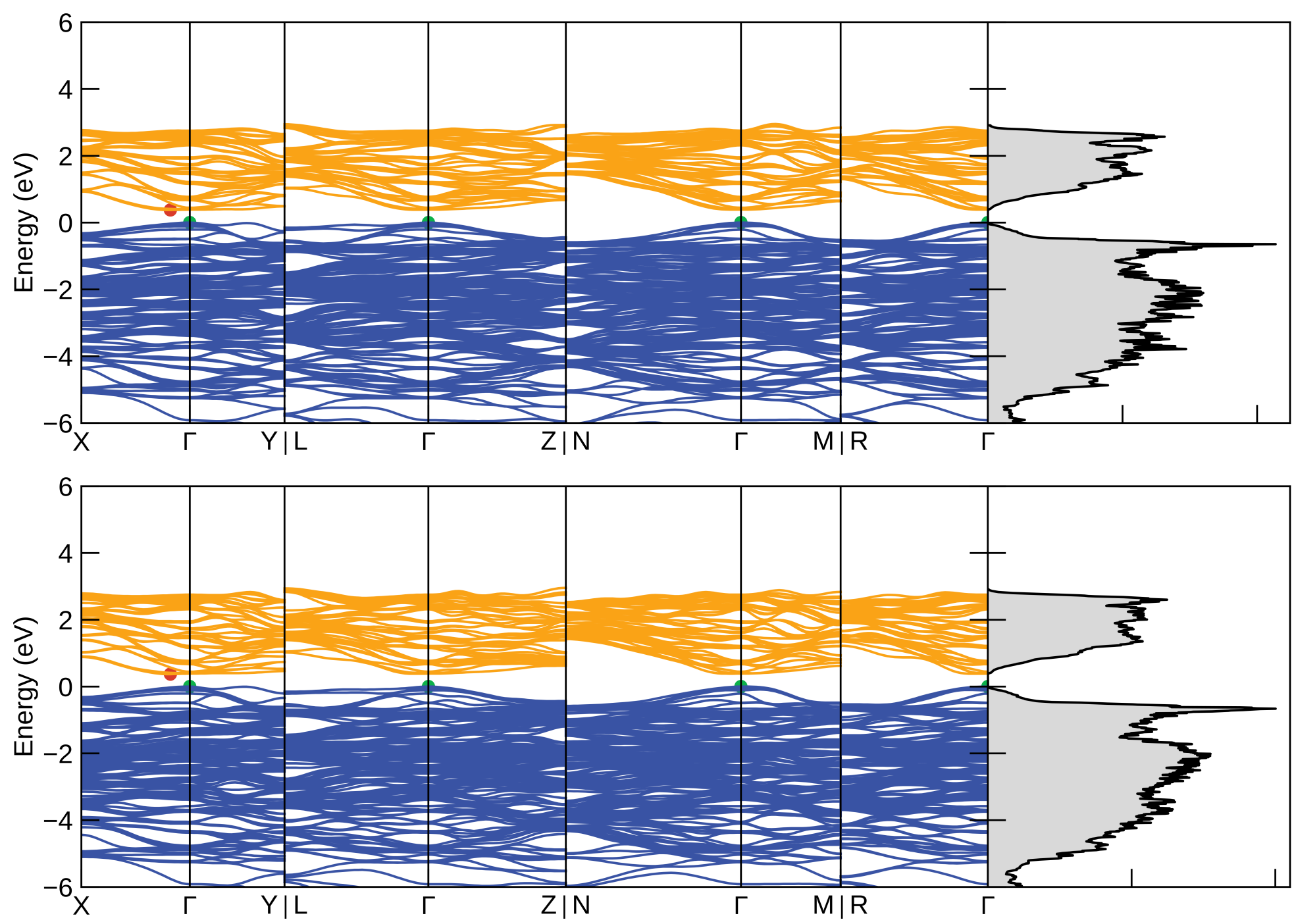

Fig. S2 Electronic band structure and density of states (DoS) of the 72-atom special quasi-random (SQS) model of $Sn(S_{0.2}Se_{0.8})$ obtained using PBEsol+D3 and $\boldsymbol{k}$-point meshes with 2 and $3\times$ more subdivisions along each reciprical lattice vector (top/bottom) than the "base" meshes used for geometry optimisation. In each band structure, the valence and conduction bands are shown in blue and orange, respectively, the valence-band minimum and conduction-band maximum (VBM/CBM) are marked with green and red solid circles, and the VBM is set to 0 eV.

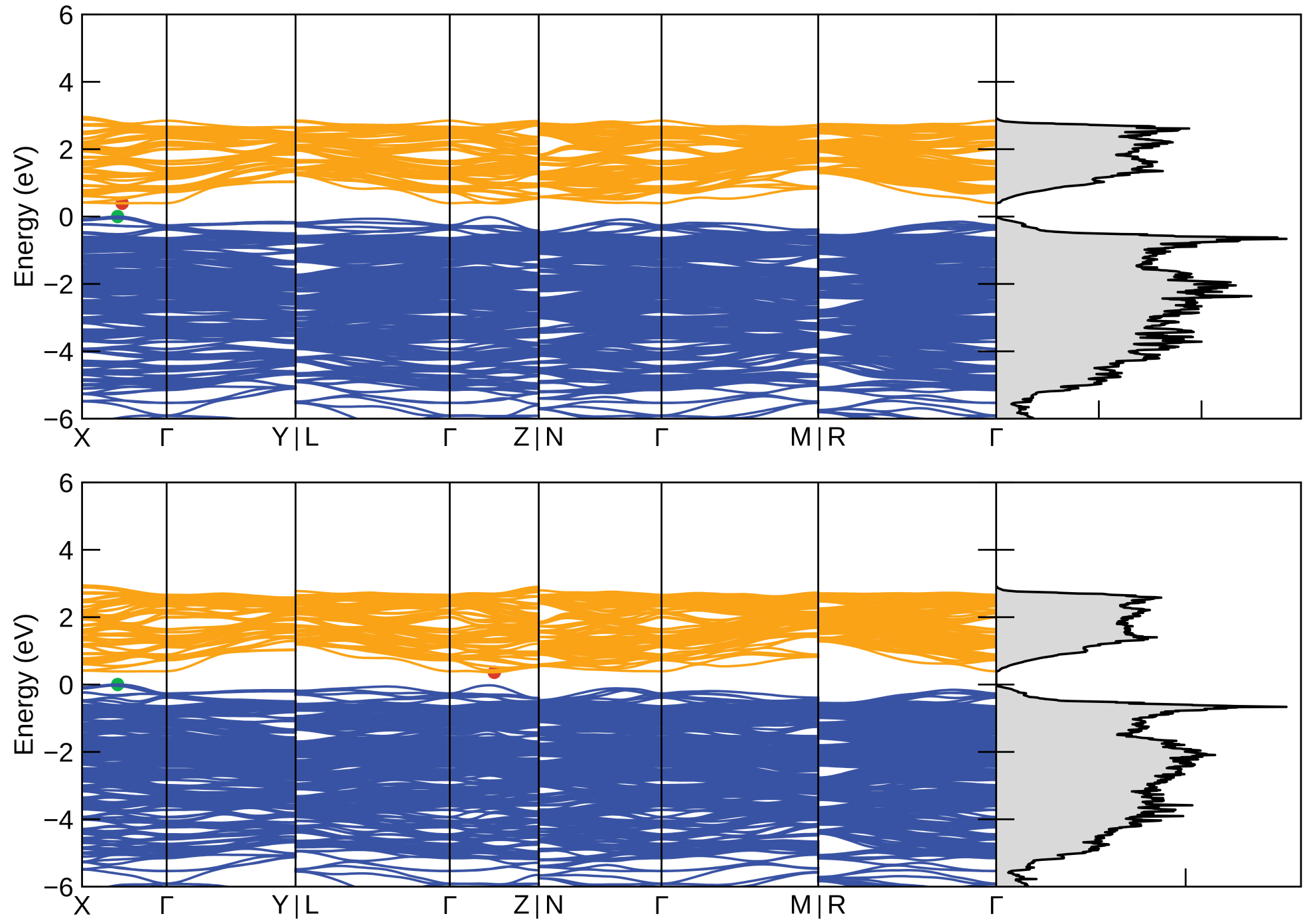

Fig. S3 Electronic band structure and density of states (DoS) of the 128-atom special quasi-random (SQS) model of $Sn(S_{0.2}Se_{0.8})$ obtained using PBEsol+D3 and ***k***-point meshes with 2 and 3× more subdivisions along each reciprical lattice vector (top/bottom) than the "base" meshes used for geometry optimisation. In each band structure, the valence and conduction bands are shown in blue and orange, respectively, the valence-band minimum and conduction-band maximum (VBM/CBM) are marked with green and red solid circles, and the VBM is set to 0 eV.

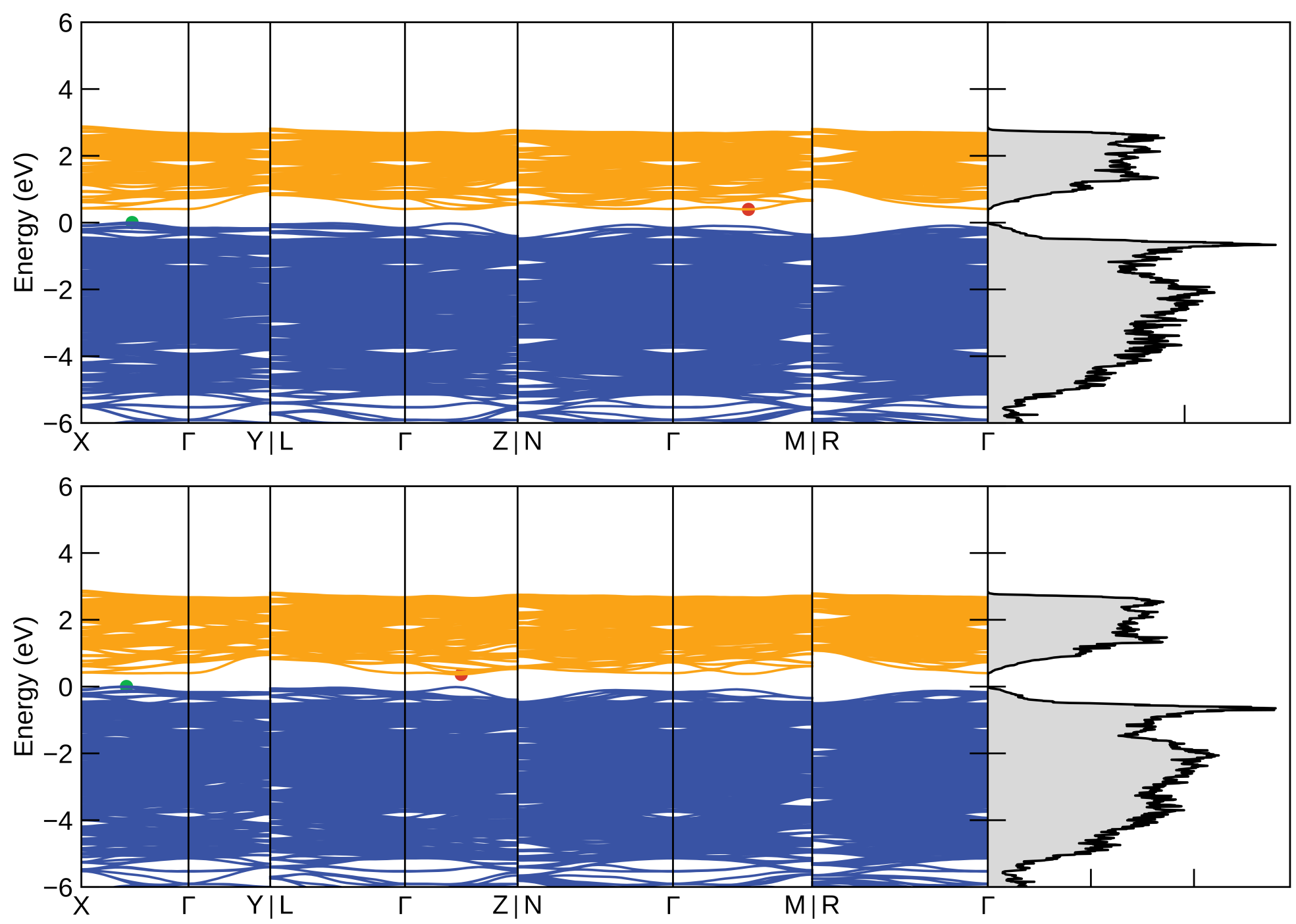

Fig. S4 Electronic band structure and density of states (DoS) of the 256-atom special quasi-random (SQS) model of $Sn(S_{0.2}Se_{0.8})$ obtained using PBEsol+D3 and ***k***-point meshes with 2 and 3× more subdivisions along each reciprical lattice vector (top/bottom) than the "base" meshes used for geometry optimisation. In each band structure, the valence and conduction bands are shown in blue and orange, respectively, the valence-band minimum and conduction-band maximum (VBM/CBM) are marked with green and red solid circles, and the VBM is set to 0 eV.

2 Structure, dynamics and lattice thermal conductivity

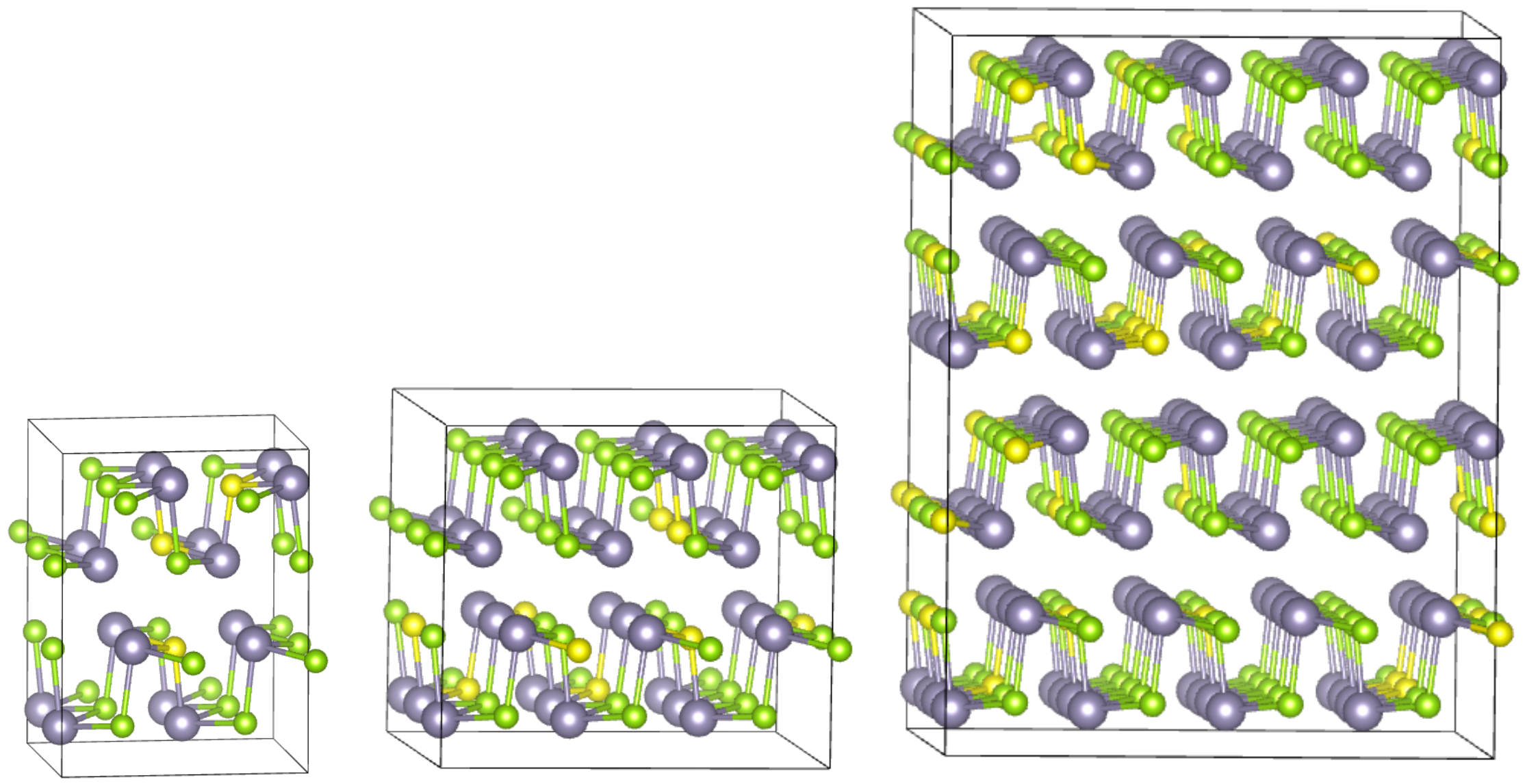

Fig. S5 Optimised structures of the 32-, 72- and 256-atom special quasi-random structure (SQS) models of $Sn(S_{0.2}Se_{0.8})$. The Sn, S and Se atoms are shown in purple, yellow and green respectively. These images were generated using the VESTA software.[1]

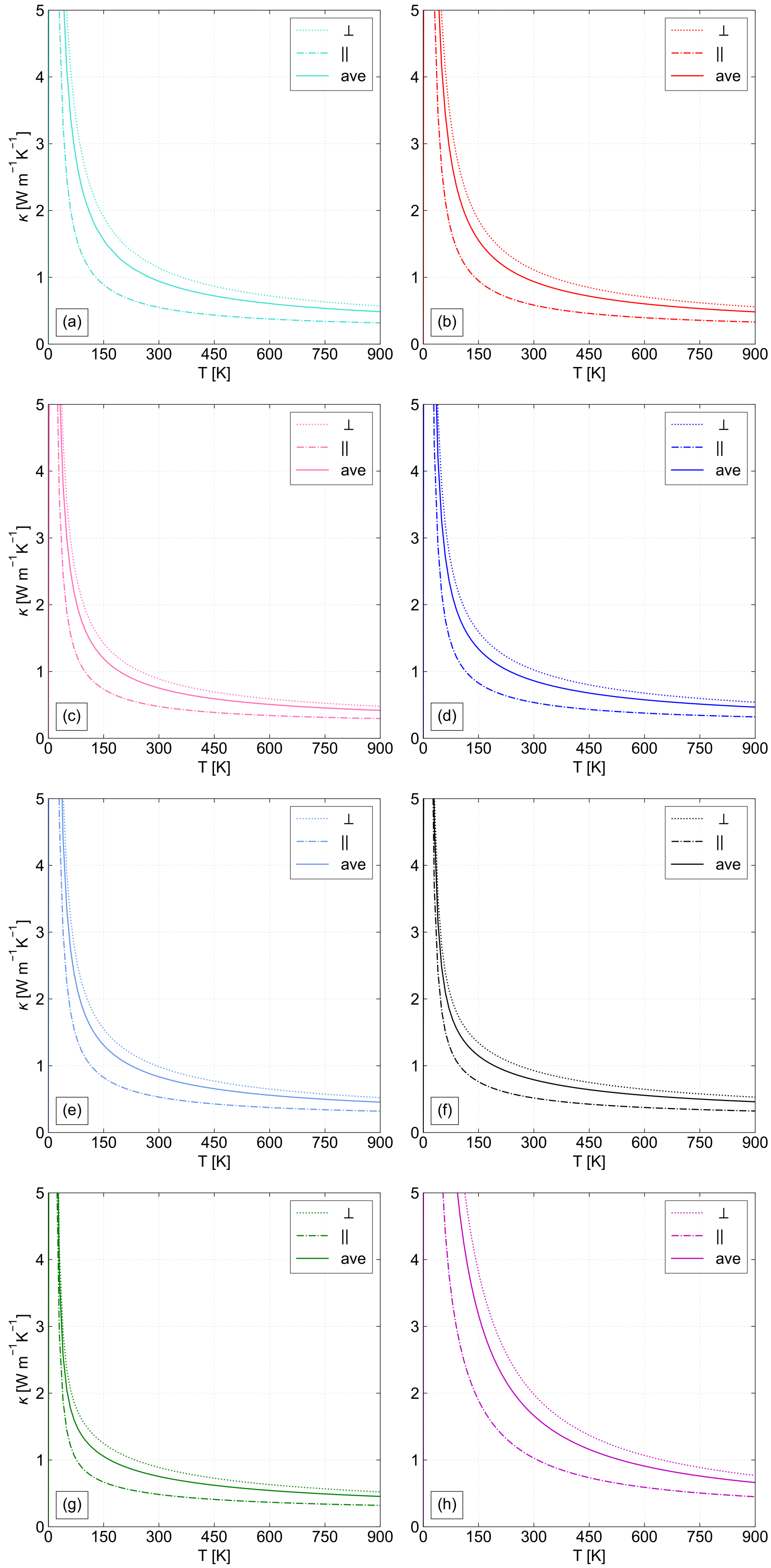

Fig. S6 Comparison of the scalar-average (solid lines), in-plane (⊥, dotted) and out-of-plane (∥, dash-dot) lattice thermal conductivity κ_{latt} of the five $Sn(S_{0.2}Se_{0.8})$ models examined in this work and SnSe: (a) 32-atom supercell enumeration model using an estimated average three-phonon interaction strength $\tilde{P}$; (b)/(c) 32-atom special quasi-random structure (SQS) model using an estimated $\tilde{P}$ (b) and explicit third-order force constants $\mathbf{\Phi}(3)$ (c); (d)/(e) 72-atom SQS model using an estimated $\tilde{P}$ (d) and explicit $\mathbf{\Phi}(3)$ obtained with a 4.5 Å interaction range cutoff (e); (f)/(g) 128- (f) and 256-atom (g) SQS models using estimated $\tilde{P}$; and (h) SnSe using explicit $\mathbf{\Phi}(3)$.[2,3]

3 Electronic transport properties

Table S3 Calculated electronic band gaps E_g of the five $Sn(S_{0.2}Se_{0.8})$ models in this work obtained using non-self-consistent HSE06.[4]

	E_g / eV
32-atom (enum)	0.64 (0.61-0.68)
32-atom (SQS)	0.63
72-atom (SQS)	0.73
128-atom (SQS)	0.88
256-atom (SQS)	0.87

Table S4 Analysis of the electrical properties of SnS, SnSe and the five $Sn(S_{0.2}Se_{0.8})$ alloy models examined in this work at a fixed extrinsic carrier concentration ("doping level") $n = 4 \times 10^{19}$ cm^{-3} and temperature $T = 700$ K, with both p- and n-type doping. For each model we show the calculated scalar-average electrical conductivity σ and Seebeck coefficient S computed with energy- and momentum-dependent electron lifetimes $\tau_{\mathbf{k}j}$ and with a fixed relaxation time $\tau_{el} = 10^{-14}$ s^{-1}. We also show the four metrics derived using the approach in Ref. 5, *viz.* the effective potential η, the conductivity and Seebeck effective masses m^*_σ / m^*_S, the Fermi surface complexity factor $N^*_V K^*$, and the weighted-average τ_{el} determined from the ratio of the two calculated σ. The calculations for SnS and SnSe were performed using data from our previous studies.[2,6]

		σ / S cm^{-1}		S / μV K^{-1}						
		$\tau_{\mathbf{k}j}$	$\tau_{el} = 10^{-14}$ s	$\tau_{\mathbf{k}j}$	$\tau_{el} = 10^{-14}$ s	η_{eff} / eV	m^*_σ / m_e	m^*_S / m_e	$N^*_V K^*$	τ_{el} / fs
32-atom (enum)	p-type	346	381	272	270	-0.39	0.30	0.87	5.00	9.08
	n-type	706	690	-241	-241	0.04	0.16	0.68	8.56	10.23
32-atom (SQS)	p-type	335	387	272	270	-0.39	0.29	0.86	5.09	8.66
	n-type	694	695	-242	-242	0.03	0.16	0.69	8.75	9.99
72-atom (SQS)	p-type	271	320	269	264	-0.30	0.35	0.82	3.55	8.47
	n-type	455	476	-255	-250	-0.08	0.24	0.73	5.43	9.56
128-atom (SQS)	p-type	332	314	250	250	-0.08	0.36	0.73	2.91	10.57
	n-type	638	550	-235	-230	0.23	0.20	0.62	5.27	11.60
256-atom (SQS)	p-type	257	285	248	244	0.01	0.40	0.70	2.33	9.02
	n-type	545	551	-236	-229	0.24	0.20	0.62	5.24	9.89
SnS	p-type	448	441	219	245	-0.01	0.26	0.7	4.56	10.16
	n-type	547	625	-230	-250	-0.09	0.18	0.73	8.20	8.75
SnSe	p-type	549	411	247	263	-0.28	0.27	0.81	5.11	13.36
	n-type	933	750	-227	-252	-0.12	0.15	0.75	11.05	12.44

Table S5 Estimated extrinsic carrier concentrations n ("doping levels") at which the p-type Seebeck coefficient S changes sign at $T = 700$ K, indicating a crossover from intrinsic to extrinsic behaviour, and n at which the S reach the peak values, for the five $Sn(S_{0.2}Se_{0.8})$ models examined in this work. Data for the SnS and SnSe endpoints taken from our previous study are shown for comparison.[2]

	$n(S=0)$ / cm^{-3}	$n(S=S_{max})$ / cm^{-3}
32-atom (enum)	2.31×10^{17}	2×10^{18}
32-atom (SQS)	2.64×10^{17}	4×10^{18}
72-atom (SQS)	6.31×10^{16}	2×10^{18}
128-atom (SQS)	2.57×10^{16}	4×10^{17}
256-atom (SQS)	3.84×10^{16}	6×10^{17}
SnS	-	2×10^{17}
SnSe	3.62×10^{16}	6×10^{17}

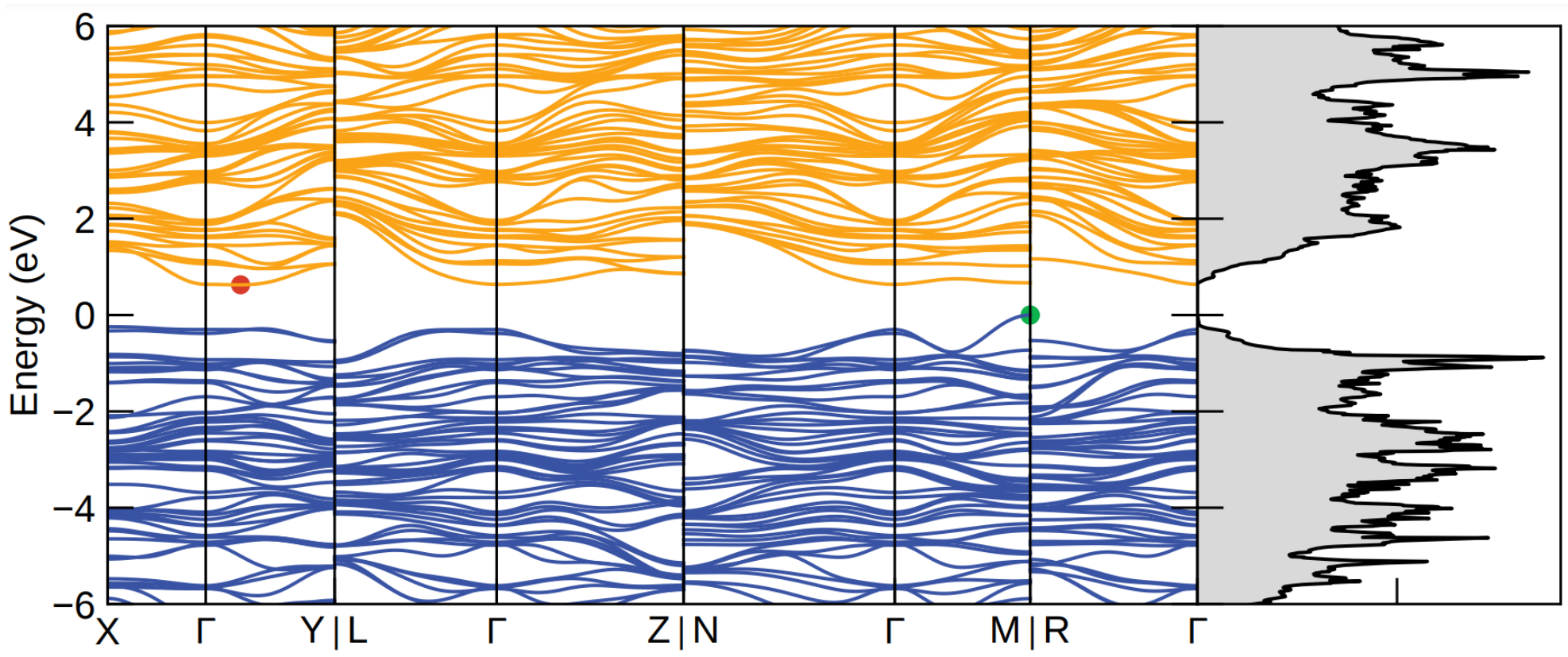

Fig. S7 Electronic band structure and density of states (DoS) of the 32-atom special quasi-random structure (SQS) alloy model of $Sn(S_{0.2}Se_{0.8})$ obtained using non-self-consistent HSE06. The valence and conduction bands are shown in blue and orange, respectively, the valence-band minimum and conduction-band maximum (VBM/CBM) are marked with green and red solid circles, and the VBM is set to 0 eV.

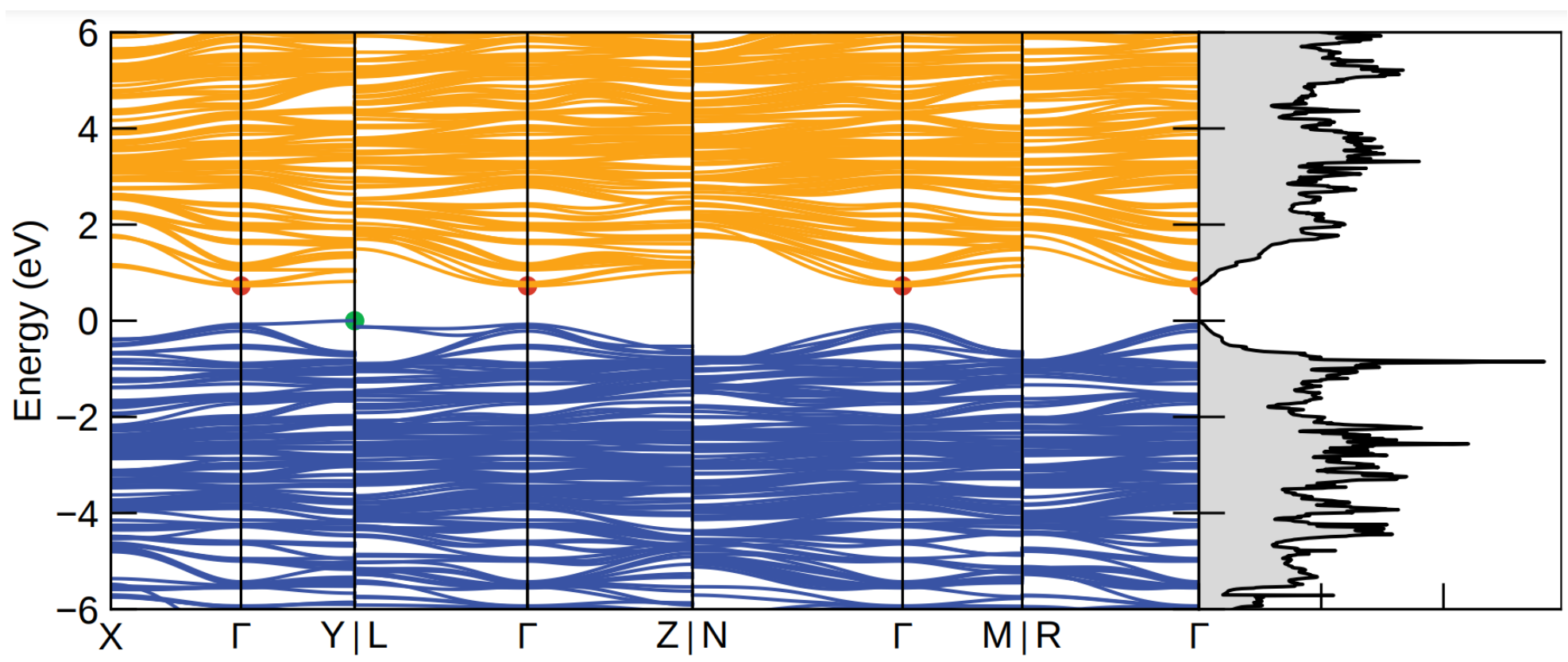

Fig. S8 Electronic band structure and density of states (DoS) of the 72-atom special quasi-random structure (SQS) alloy model of $Sn(S_{0.2}Se_{0.8})$ obtained using non-self-consistent HSE06. The valence and conduction bands are shown in blue and orange, respectively, the valence-band minimum and conduction-band maximum (VBM/CBM) are marked with green and red solid circles, and the VBM is set to 0 eV.

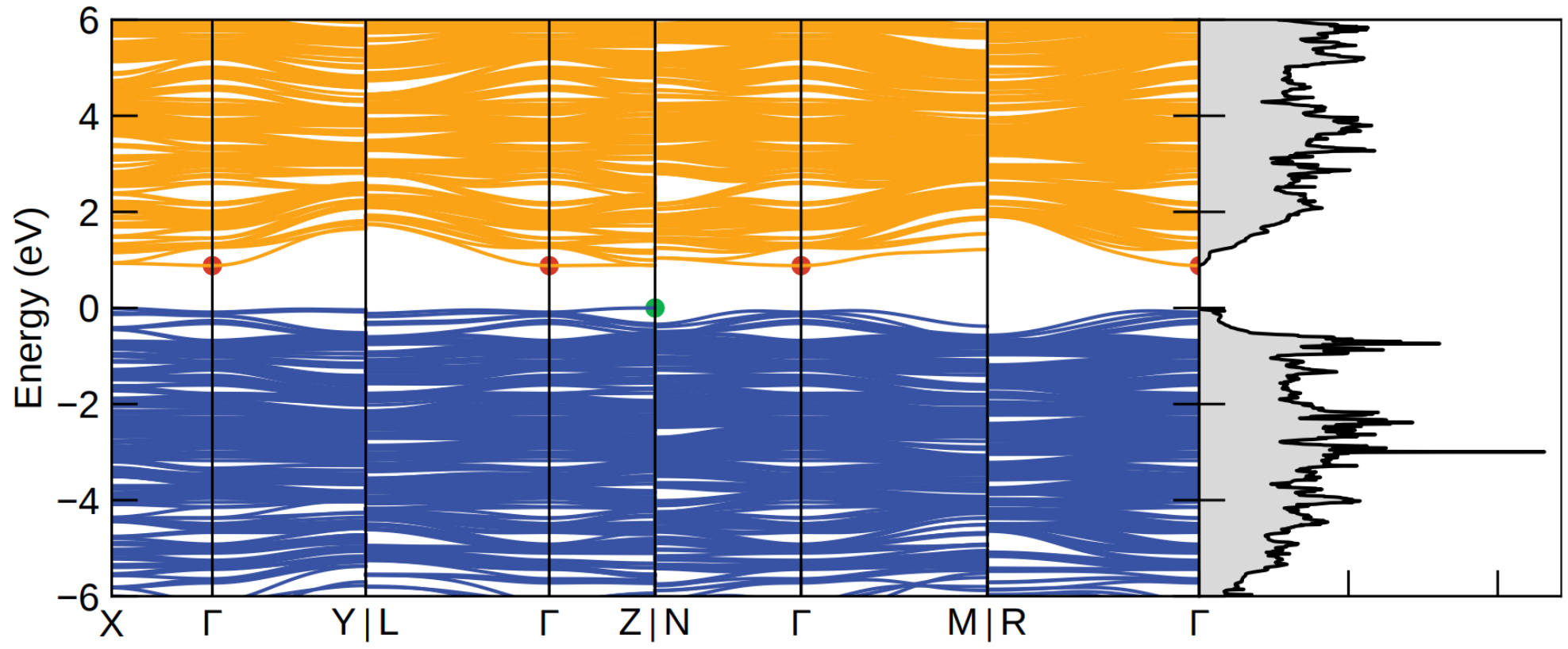

Fig. S9 Electronic band structure and density of states (DoS) of the 128-atom special quasi-random structure (SQS) model of $Sn(S_{0.2}Se_{0.8})$ obtained using non-self-consistent HSE06. The valence and conduction bands are shown in blue and orange, respectively, the valence-band minimum and conduction-band maximum (VBM/CBM) are marked with green and red solid circles, and the VBM is set to 0 eV.

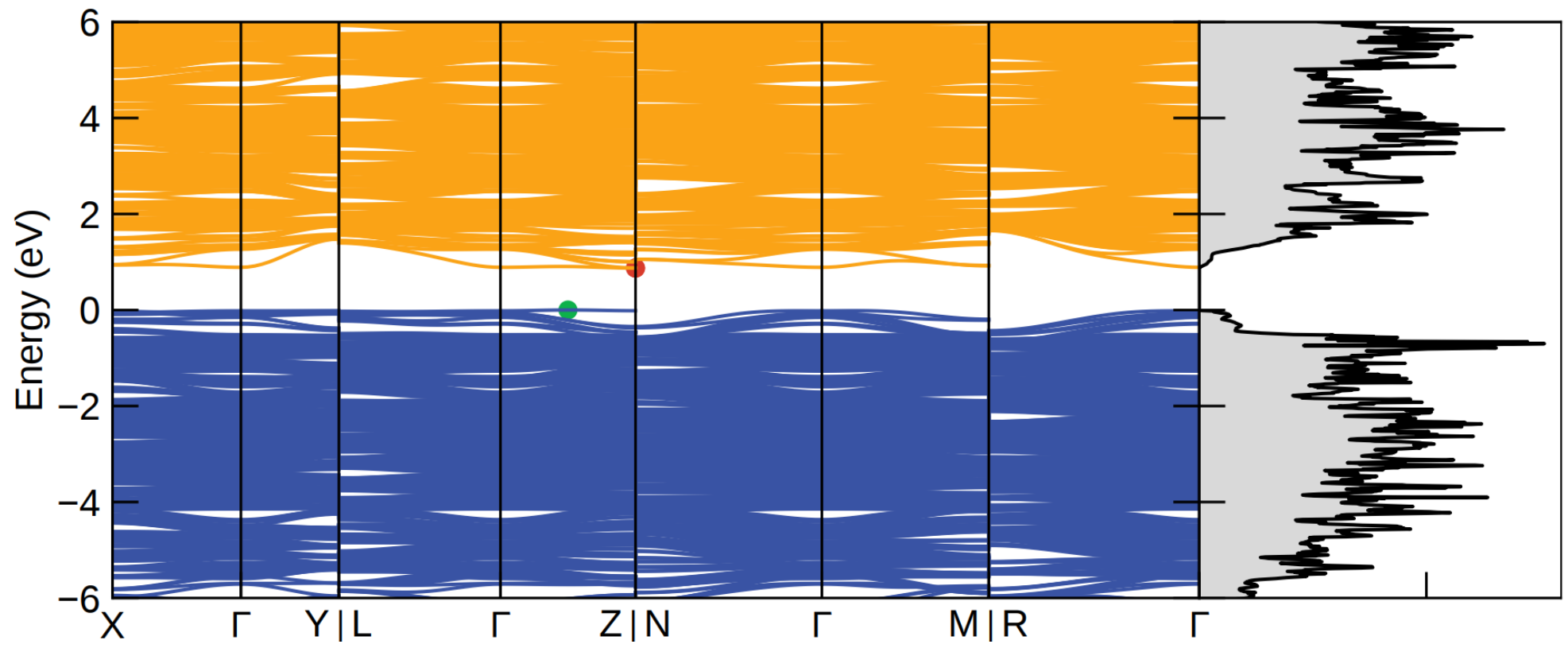

Fig. S10 Electronic band structure and density of states (DoS) of the 256-atom special quasi-random structure (SQS) alloy model of $Sn(S_{0.2}Se_{0.8})$ obtained using non-self-consistent HSE06. The valence and conduction bands are shown in blue and orange, respectively, the valence-band minimum and conduction-band maximum (VBM/CBM) are marked with green and red solid circles, and the VBM is set to 0 eV.

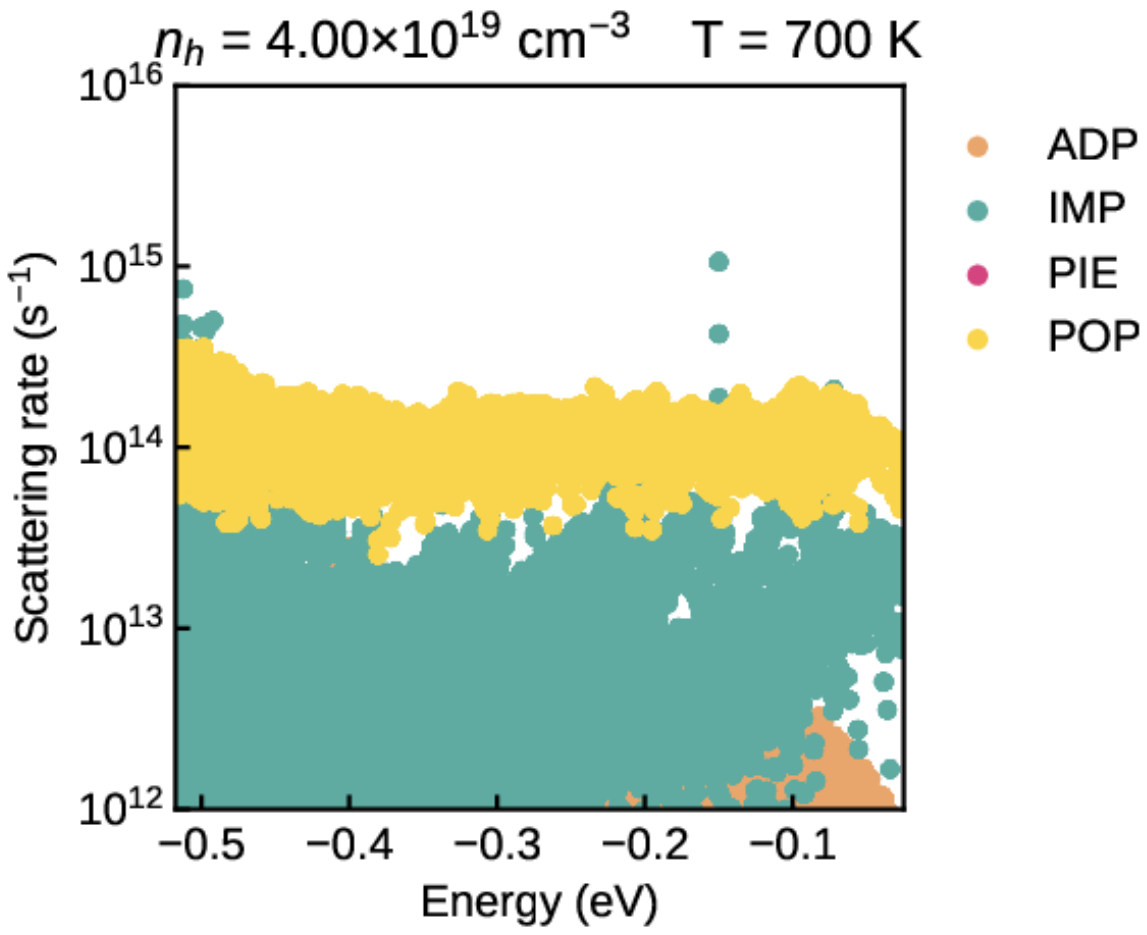

Fig. S11 Calculated electron scattering rates Γ_{kj} (inverse lifetimes τ_{kj}) as a function of energy for the p- and n-type 32-atom special quasi-random structure (SQS) model of $Sn(S_{0.2}Se_{0.8})$ (left/right) at an extrinsic hole/electron carrier concentration ("doping level") $n = 4 \times 10^{19}$ cm^{-3} and a temperature $T = 700$ K. The valence-band maximum (VBM) is set to 0 eV.

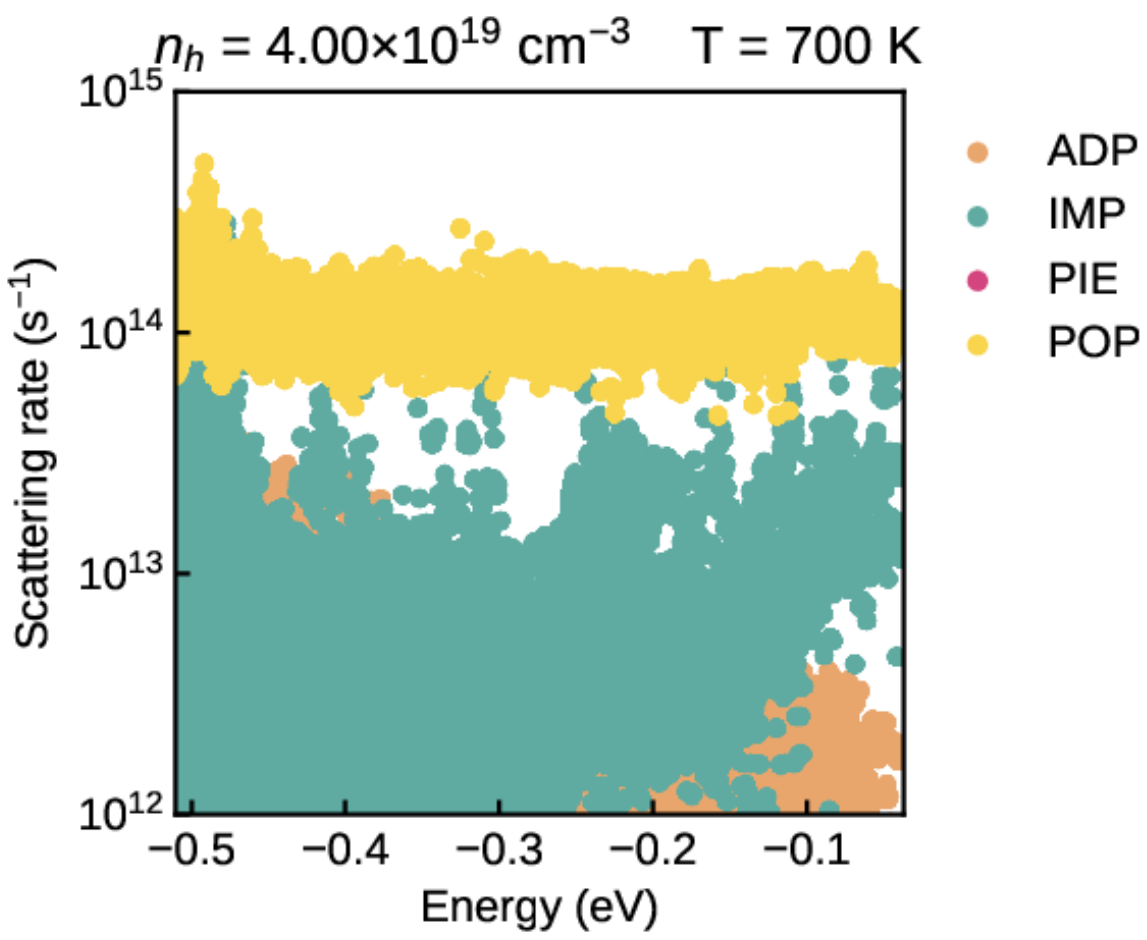

Fig. S12 Calculated electron scattering rates Γ_{kj} (inverse lifetimes τ_{kj}) as a function of energy for the p- and n-type 72-atom special quasi-random structure (SQS) model of $Sn(S_{0.2}Se_{0.8})$ (left/right) at an extrinsic hole/electron carrier concentration ("doping level") $n = 4 \times 10^{19}$ cm^{-3} and a temperature $T = 700$ K. The valence-band maximum (VBM) is set to 0 eV.

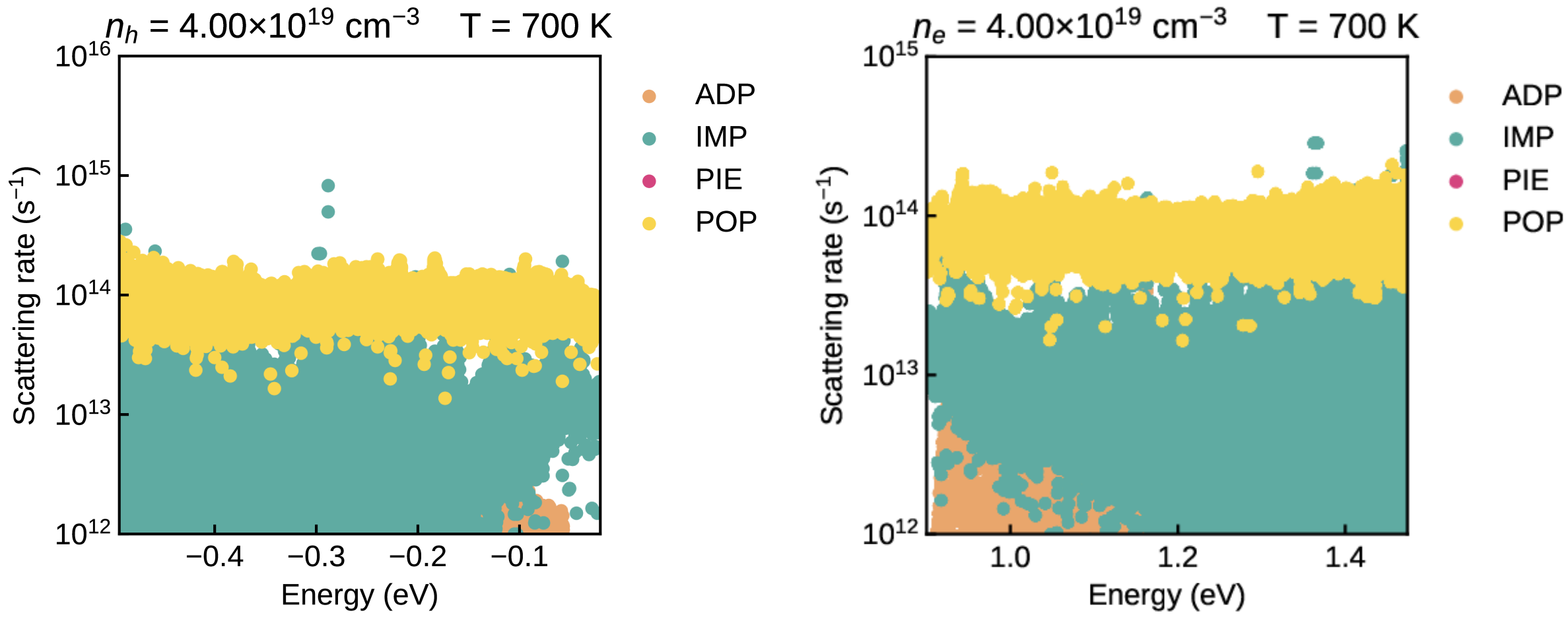

Fig. S13 Calculated electron scattering rates $\Gamma_{\boldsymbol{k}j}$ (inverse lifetimes $\tau_{\boldsymbol{k}j}$) as a function of energy for the p- and n-type 128-atom special quasi-random structure (SQS) model of Sn($S_{0.2}Se_{0.8}$) (left/right) at an extrinsic hole/electron carrier concentration ("doping level") $n = 4 \times 10^{19}$ cm^{-3} and a temperature $T = 700$ K. The valence-band maximum (VBM) is set to 0 eV.

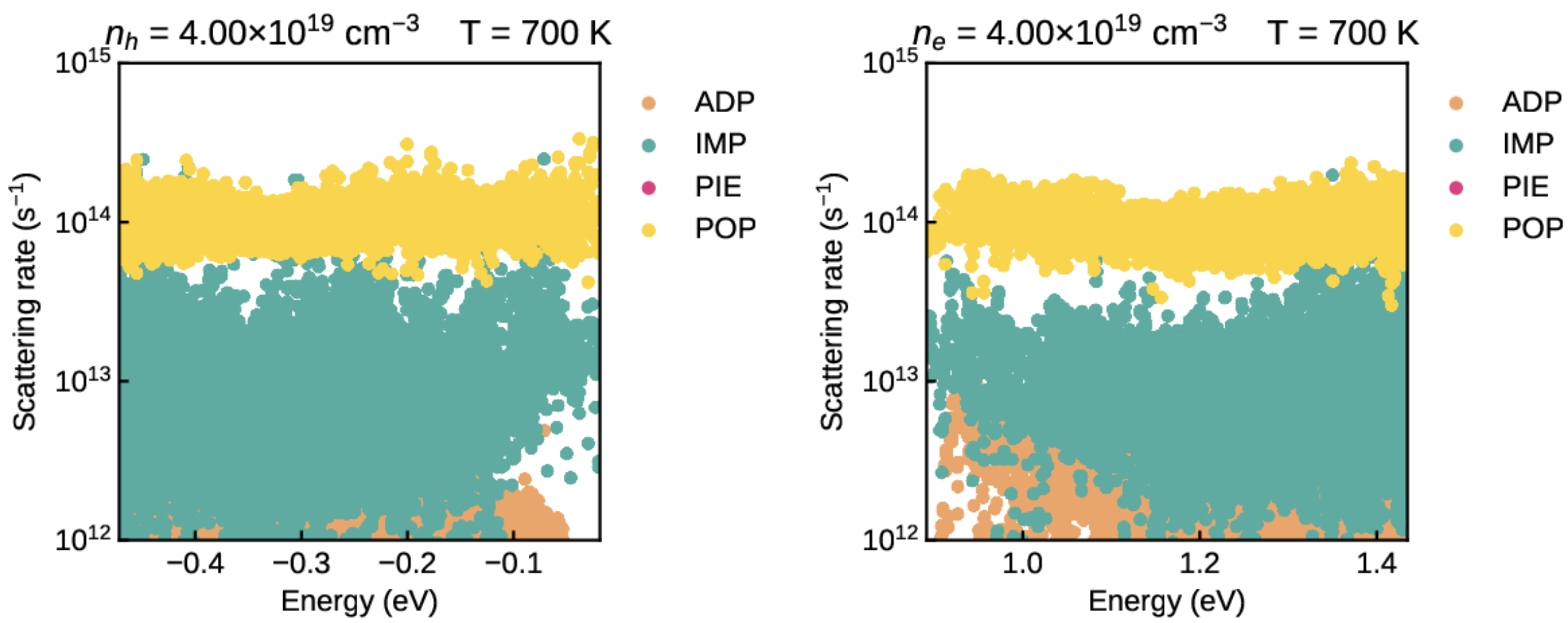

Fig. S14 Calculated electron scattering rates $\Gamma_{\boldsymbol{k}j}$ (inverse lifetimes $\tau_{\boldsymbol{k}j}$) as a function of energy for the p- and n-type 256-atom special quasi-random structure (SQS) model of Sn($S_{0.2}Se_{0.8}$) (left/right) at an extrinsic hole/electron carrier concentration ("doping level") $n = 4 \times 10^{19}$ cm^{-3} and a temperature $T = 700$ K. The valence-band maximum (VBM) is set to 0 eV.

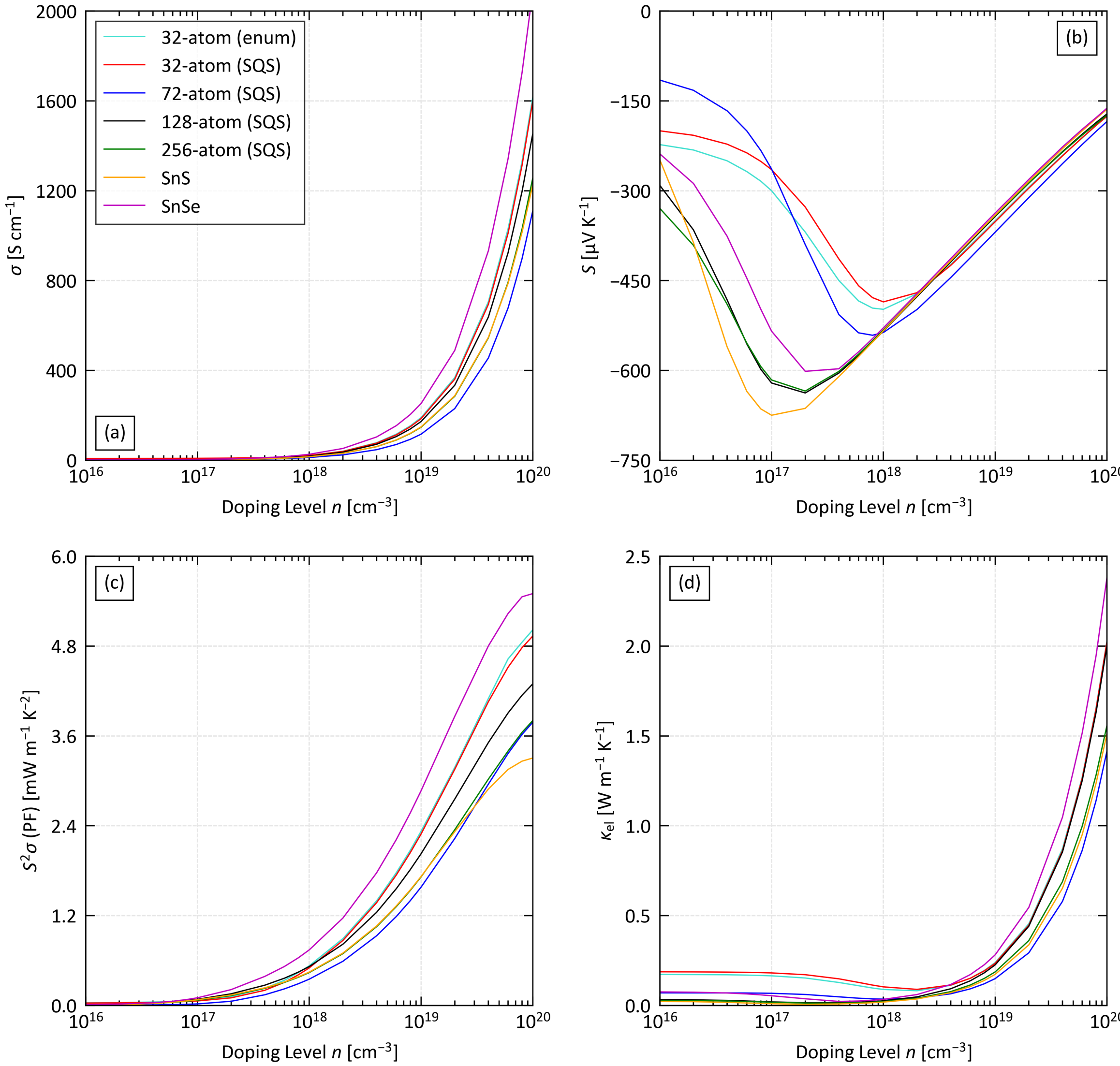

Fig. S15 Predicted scalar-average electrical transport properties of the five $Sn(S_{0.2}Se_{0.8})$ models examined in this work as a function of extrinsic electron carrier concentration n ("doping level") at a fixed T = 700 K: (a) electrical conductivity σ, (b) Seebeck coefficient S, (c) power factor $S^2\sigma$ (PF) and (d) electronic thermal conductivity κ_{el}. Data for the SnS and SnSe endpoints taken from our previous studies are shown for comparison.[2,6]

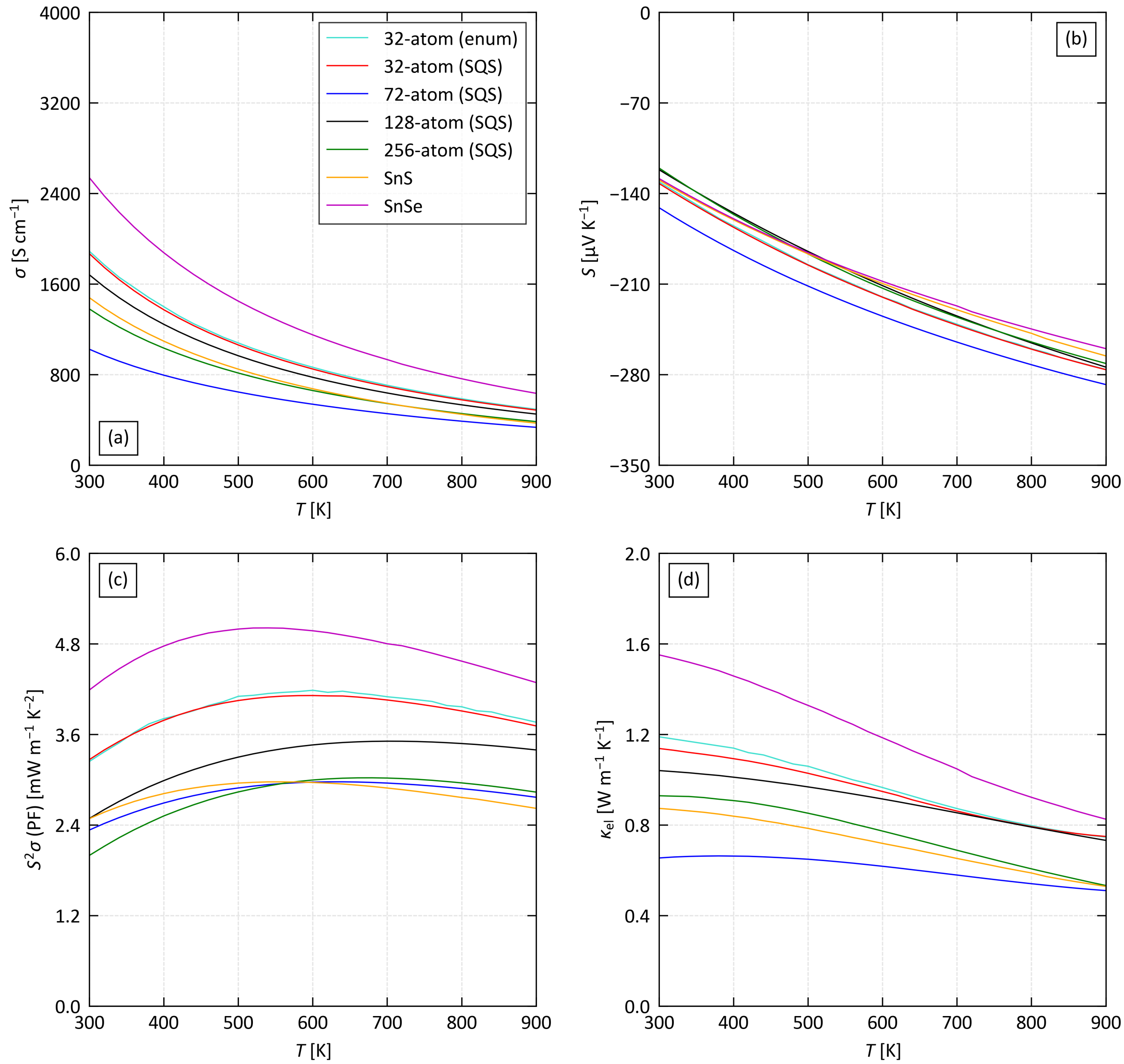

Fig. S16 Predicted scalar-average electrical properties of the five $Sn(S_{0.2}Se_{0.8})$ models examined in this work as a function of temperature at a fixed extrinsic electron carrier concentration ("doping level") $n = 4 \times 10^{19}$ cm^{-3}: (a) electrical conductivity σ, (b) Seebeck coefficient S, (c) power factor $S^2\sigma$ (PF) and (d) electronic thermal conductivity κ_{el}. Data for the SnS and SnSe endpoints taken from our previous study are shown for comparison.[2,6]

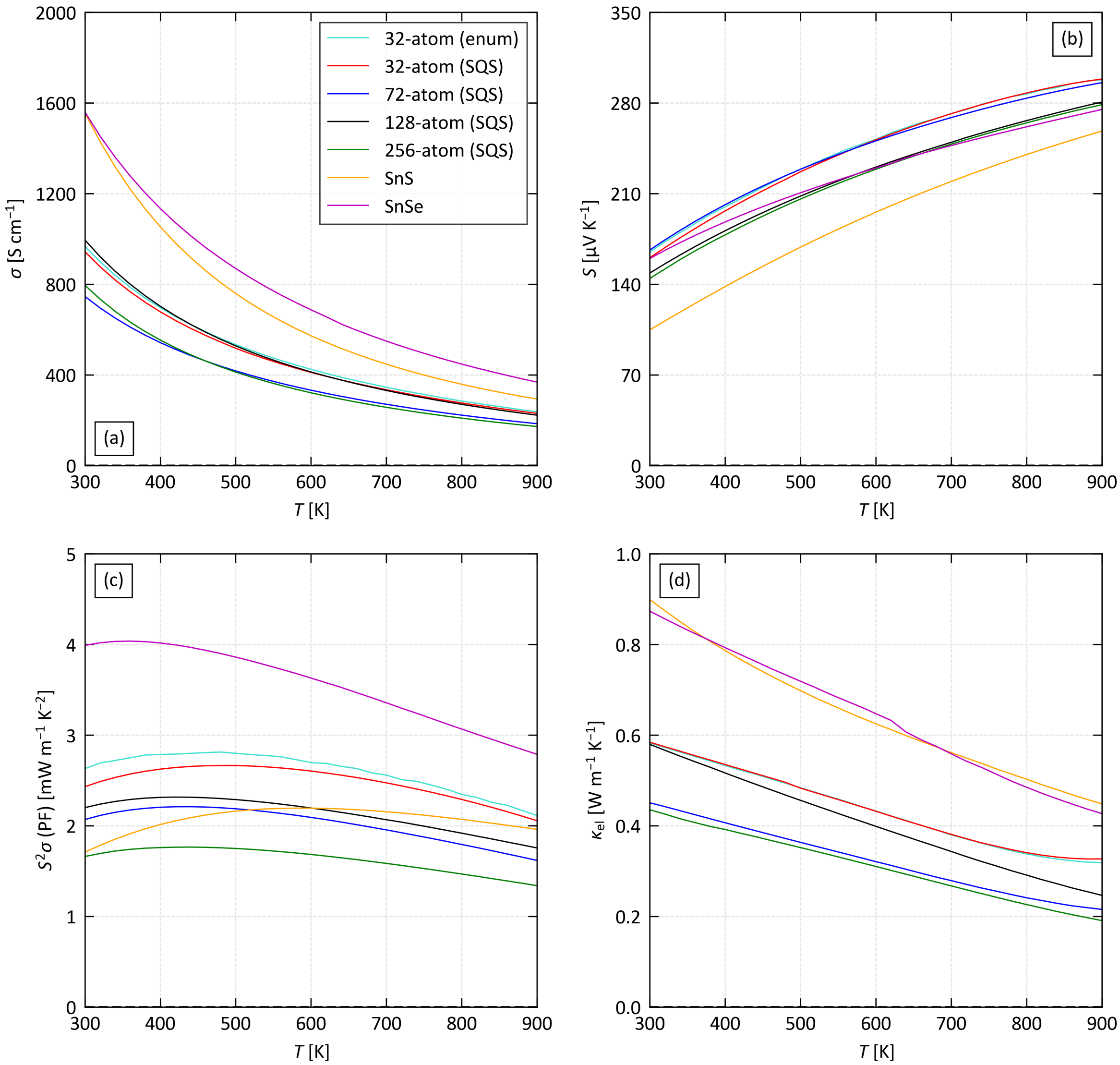

Fig. S17 Predicted scalar-average electrical properties of the five $Sn(S_{0.2}Se_{0.8})$ models examined in this work as a function of temperature at a fixed extrinsic hole carrier concentration ("doping level") $n = 4 \times 10^{19}$ cm^{-3}: (a) electrical conductivity σ, (b) Seebeck coefficient S, (c) power factor $S^2\sigma$ (PF) and (d) electronic thermal conductivity κ_{el}. Data for the SnS and SnSe endpoints taken from our previous study are shown for comparison.[2]

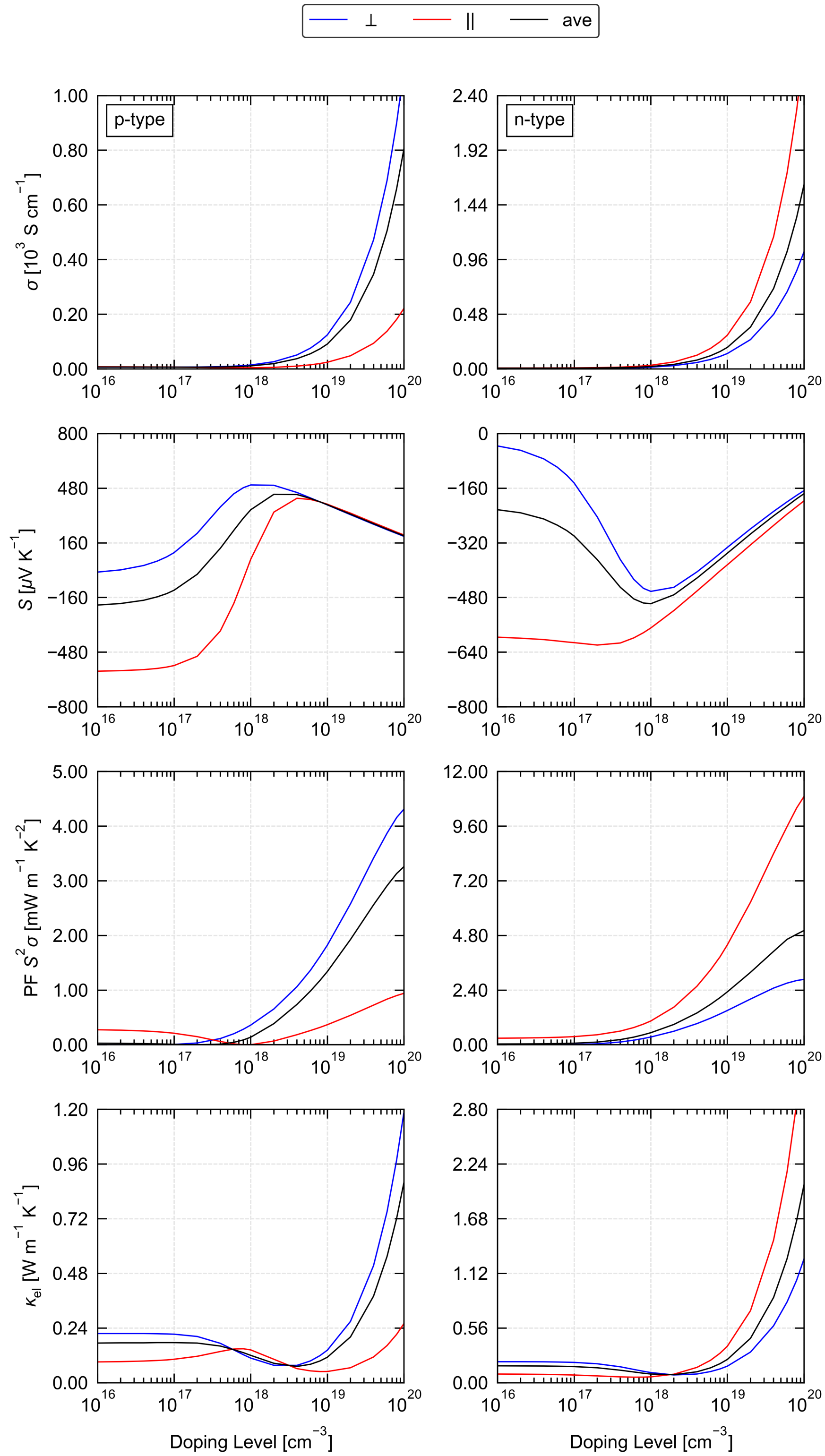

Fig. S18 Comparison of the predicted scalar-average, in-plane ($\perp$) and out-of-plane ($\parallel$) electrical conductivity σ, Seebeck coefficient S, power factor $S^2\sigma$ (PF) and electronic thermal conductivity κ_{el} of the p-type (left column) and n-type (right column) 32-atom supercell enumeration model of $Sn(S_{0.2}Se_{0.8})$ as a function of extrinsic carrier concentration ("doping level") n at a fixed temperature $T = 700$ K.

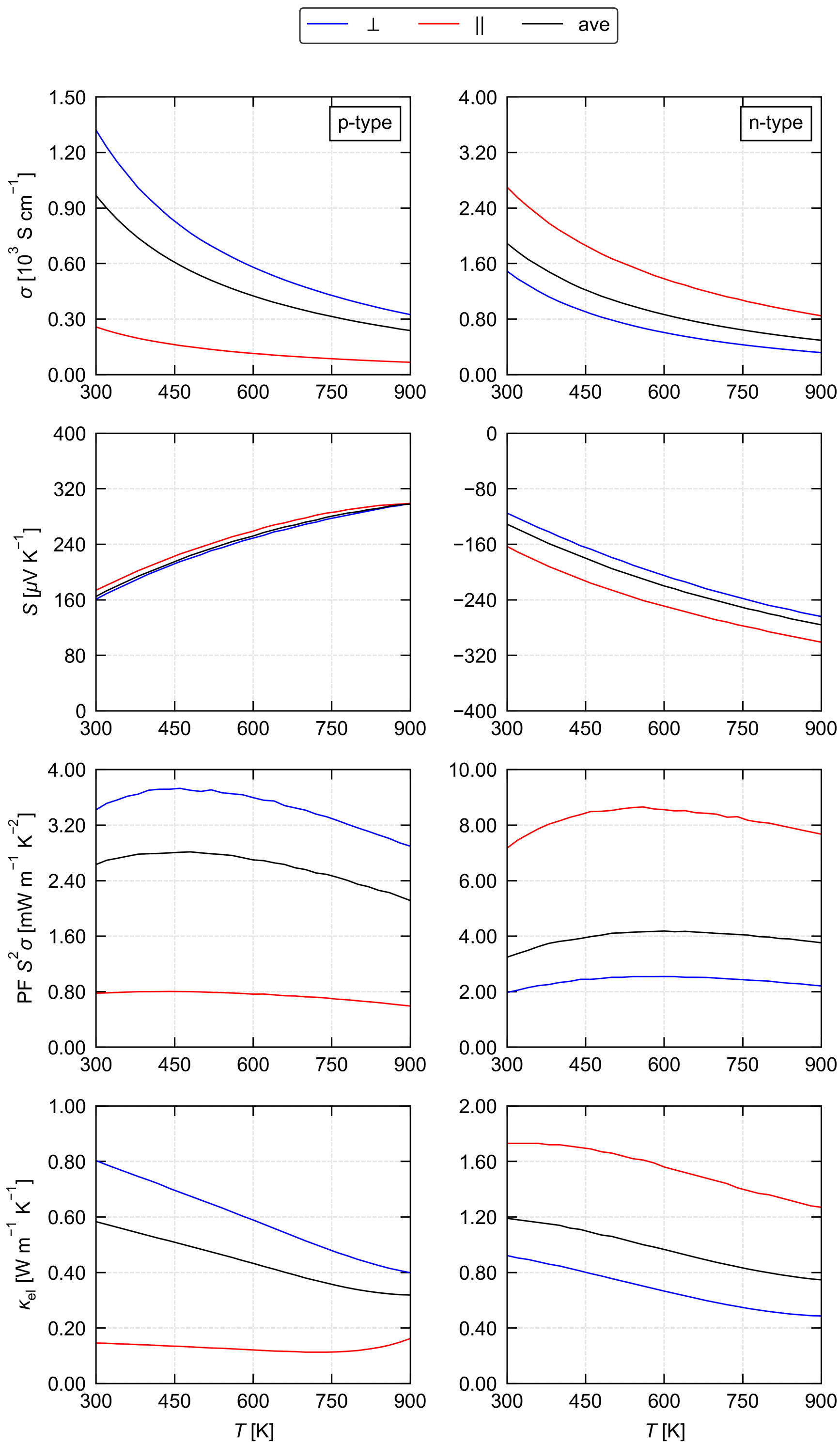

Fig. S19 Comparison of the predicted scalar-average, in-plane (⊥) and out-of-plane (∥) electrical conductivity σ, Seebeck coefficient S, power factor $S^2\sigma$ (PF) and electronic thermal conductivity κ_{el} of the p-type (left column) and n-type (right column) 32-atom supercell enumeration model of $Sn(S_{0.2}Se_{0.8})$ as a function of temperature at a fixed extrinsic carrier concentration ("doping level") $n = 4 \times 10^{19}$ cm^{-3}.

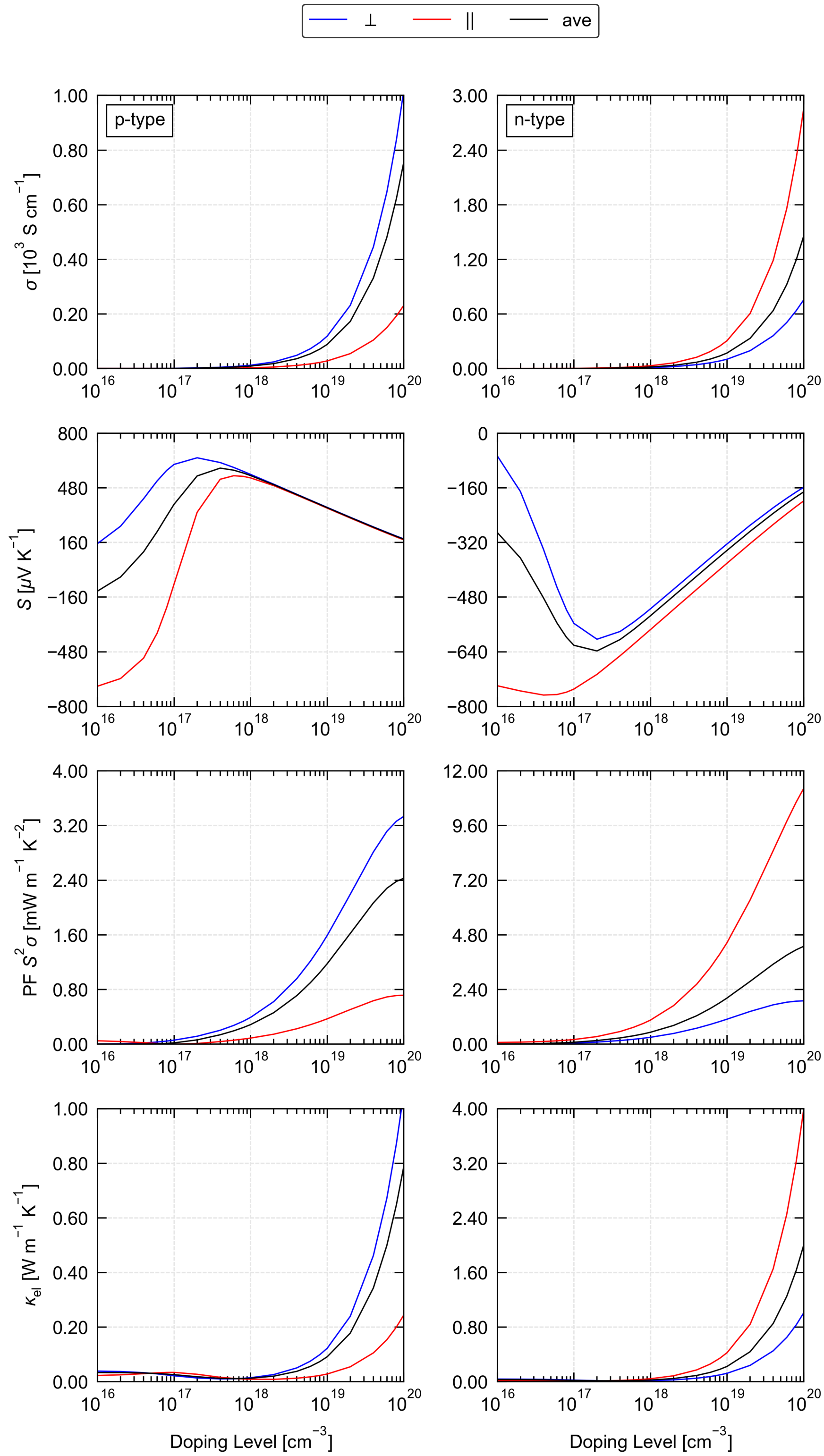

Fig. S20 Comparison of the predicted scalar-average, in-plane (⊥) and out-of-plane (∥) electrical conductivity σ, Seebeck coefficient S, power factor $S^2\sigma$ (PF) and electronic thermal conductivity κ_{el} of the p-type (left column) and n-type (right column) 128-atom special quasi-random structure (SQS) model of $Sn(S_{0.2}Se_{0.8})$ as a function of extrinsic carrier concentration ("doping level") n at a fixed temperature $T = 700$ K.

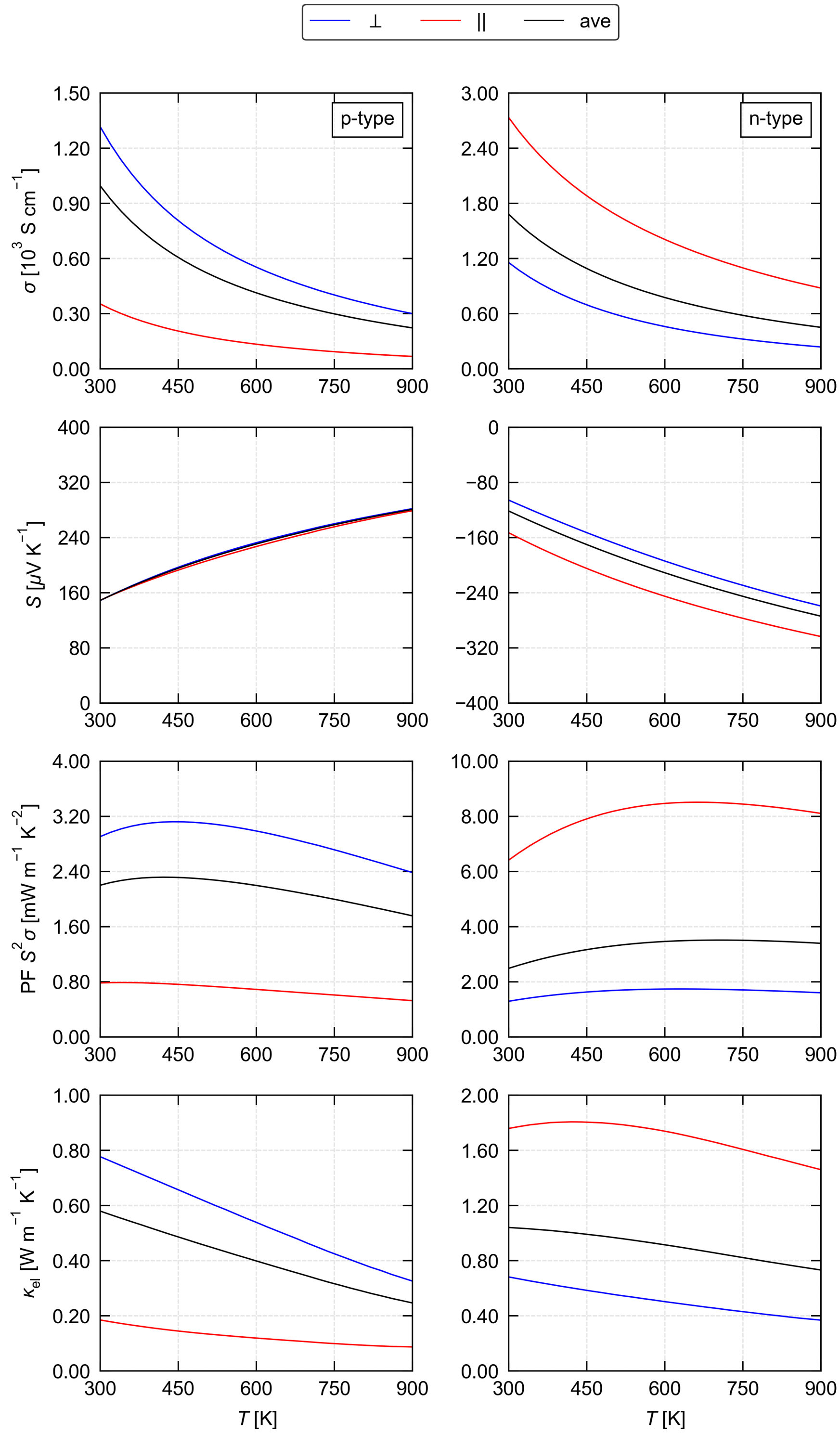

Fig. S21 Comparison of the predicted scalar-average, in-plane (⊥) and out-of-plane (∥) electrical conductivity σ, Seebeck coefficient S, power factor $S^2\sigma$ (PF) and electronic thermal conductivity κ_{el} of the p-type (left column) and n-type (right column) 128-atom special quasi-random structure (SQS) model of $Sn(S_{0.2}Se_{0.8})$ as a function of temperature at a fixed extrinsic carrier concentration ("doping level") $n = 4 \times 10^{19}$ cm^{-3}.

4 Figure of merit zT

Table S6 Predicted maximum thermoelectric figure of merit zT_{max} of the 23 individual structures in the 32-atom supercell enumeration model of $Sn(S_{0.2}Se_{0.8})$ with p-type doping. For each model we list the extrinsic carrier concentration ("doping level") n and temperature at which the zT_{max} is attained, together with the associated electrical conductivity σ, Seebeck coefficient S, power factor $S^2\sigma$ (PF), and electronic, lattice and total thermal conductivity κ_{el}, κ_{latt} and κ. Predictions for the 32-atom special quasi-random structure (SQS) model are also shown for comparison. The κ_{latt} of all the models, including the SQS model, was computed using averaged three-phonon interaction strength $\tilde{P}$ estimated from the endpoints.

				σ	S	PF $S^2\sigma$	κ [W m^{-1} K^{-1}]		
	n [cm^{-3}]	T [K]	zT	[S cm^{-1}]	[μV K^{-1}]	[mW m^{-1} K^{-2}]	κ_{el}	κ_{latt}	κ
1	4×10^{19}	800	2.20	295	286	2.41	0.36	0.52	0.88
2	4×10^{19}	800	2.23	300	286	2.45	0.36	0.52	0.88
3	4×10^{19}	800	2.18	282	288	2.33	0.34	0.51	0.85
4	4×10^{19}	800	2.25	286	288	2.36	0.33	0.51	0.84
5	4×10^{19}	800	2.19	297	283	2.37	0.35	0.51	0.86
6	4×10^{19}	800	2.17	286	287	2.36	0.34	0.53	0.87
7	4×10^{19}	800	2.24	276	290	2.32	0.32	0.51	0.83
8	4×10^{19}	800	2.17	290	284	2.34	0.34	0.52	0.86
9	4×10^{19}	800	2.21	293	285	2.37	0.35	0.51	0.86
10	4×10^{19}	800	2.19	296	283	2.37	0.35	0.51	0.86
11	4×10^{19}	800	2.21	286	287	2.36	0.34	0.52	0.86
12	4×10^{19}	800	2.17	272	291	2.30	0.31	0.54	0.85
13	4×10^{19}	800	2.26	299	285	2.42	0.35	0.51	0.86
14	4×10^{19}	800	2.23	286	287	2.35	0.34	0.51	0.85
15	4×10^{19}	800	2.22	287	284	2.32	0.33	0.51	0.84
16	4×10^{19}	800	2.18	280	288	2.32	0.33	0.52	0.85
17	4×10^{19}	800	2.24	271	293	2.32	0.33	0.50	0.83
18	4×10^{19}	800	2.10	273	287	2.25	0.33	0.53	0.86
19	4×10^{19}	800	2.23	270	291	2.28	0.32	0.50	0.82
20	4×10^{19}	800	2.27	281	292	2.39	0.34	0.50	0.84
21	4×10^{19}	800	2.27	282	292	2.41	0.35	0.50	0.85
22	4×10^{19}	800	2.18	271	294	2.34	0.33	0.53	0.86
23	4×10^{19}	800	2.20	261	297	2.30	0.31	0.52	0.83
SQS	4×10^{19}	800	2.15	277	288	2.29	0.34	0.51	0.85

Table S7 Predicted maximum thermoelectric figure of merit zT_{max} of the 23 individual structures in the 32-atom supercell enumeration model of $Sn(S_{0.2}Se_{0.8})$ with n-type doping. For each model we list the extrinsic carrier concentration ("doping level") n and temperature at which the zT_{max} is attained, together with the associated electrical conductivity σ, Seebeck coefficient S, power factor $S^2\sigma$ (PF), and electronic, lattice and total thermal conductivity κ_{el}, κ_{latt} and κ. Predictions for the 32-atom special quasi-random structure (SQS) model are also shown for comparison. The κ_{latt} of all the models, including the SQS model, was computed using averaged three-phonon interaction strength $\tilde{P}$ estimated from the endpoints.

	n [cm^{-3}]	T [K]	zT	σ [S cm^{-1}]	S [μV K^{-1}]	$S^2\sigma$ (PF) [mW m^{-1} K^{-2}]	κ [W m^{-1} K^{-1}]		
							κ_{el}	κ_{latt}	κ
1	2×10^{19}	800	2.58	304	-317	3.06	0.43	0.52	0.95
2	2×10^{19}	800	2.60	315	-315	3.12	0.44	0.52	0.96
3	2×10^{19}	800	2.57	308	-314	3.03	0.43	0.51	0.94
4	2×10^{19}	800	2.57	313	-311	3.03	0.44	0.51	0.95
5	2×10^{19}	800	2.55	304	-315	3.01	0.43	0.51	0.94
6	2×10^{19}	800	2.42	296	-312	2.87	0.42	0.53	0.95
7	2×10^{19}	800	2.60	293	-318	2.96	0.41	0.51	0.92
8	2×10^{19}	800	2.57	300	-318	3.03	0.42	0.52	0.94
9	2×10^{19}	800	2.56	290	-319	2.95	0.41	0.51	0.92
10	2×10^{19}	800	2.59	312	-314	3.08	0.44	0.51	0.95
11	2×10^{19}	800	2.55	299	-316	2.98	0.42	0.52	0.94
12	2×10^{19}	800	2.41	299	-309	2.86	0.41	0.54	0.95
13	2×10^{19}	800	2.63	323	-312	3.15	0.45	0.51	0.96
14	2×10^{19}	800	2.66	329	-310	3.17	0.44	0.51	0.95
15	2×10^{19}	800	2.56	296	-314	2.91	0.40	0.51	0.91
16	2×10^{19}	800	2.58	302	-316	3.03	0.42	0.52	0.94
17	2×10^{19}	800	2.66	305	-316	3.05	0.41	0.50	0.91
18	2×10^{19}	800	2.47	307	-308	2.92	0.42	0.53	0.95
19	2×10^{19}	800	2.60	305	-313	2.98	0.41	0.50	0.91
20	2×10^{19}	800	2.57	298	-314	2.94	0.41	0.50	0.91
21	2×10^{19}	800	2.67	330	-310	3.16	0.45	0.50	0.95
22	2×10^{19}	800	2.49	292	-315	2.90	0.40	0.53	0.93
23	2×10^{19}	800	2.49	301	-310	2.90	0.41	0.52	0.93
SQS	2×10^{19}	800	2.53	299	-315	2.96	0.42	0.51	0.93

Table S8 Predicted maximum thermoelectric figure of merit, zT_{max}, of the five $Sn(S_{0.2}Se_{0.8})$ models examined in this work with both p- and n-type doping. For each model we list the extrinsic carrier concentration ("doping level") n and temperature at which the zT_{max} is attained, together with the associated electrical conductivity σ, Seebeck coefficient S, power factor $S^2\sigma$ (PF), and electronic, lattice and total thermal conductivity κ_{el}, κ_{latt} and κ. For each entry we also compare the predicted scalar-average, in-plane ($\perp$) and out-of-plane ($\parallel$) zT_{max} and constituent properties. Data for the SnS and SnSe endpoints taken from our previous work are shown for comparison,[2,6] and the κ_{latt} include the contribution from intraband tunnelling.[7] The κ_{latt} of all five alloy models was computed with estimated average three-phonon interaction strengths $\tilde{P}$.

						σ	S	$S^2\sigma$ (PF)	κ [W m^{-1} K^{-1}]		
		n [cm^{-3}]	T [K]		zT	[S cm^{-1}]	[μV K^{-1}]	[mW m^{-1} K^{-2}]	κ_{el}	κ_{latt}	κ_{tot}
p-type	32-atom (enum)	4×10^{19}	800	$\perp$	2.28	389	285	3.16	0.45	0.61	1.06
				$\parallel$	1.19	78	292	0.67	0.12	0.33	0.45
				ave	2.21	285	287	2.36	0.34	0.52	0.86
	32-atom (SQS)	4×10^{19}	800	$\perp$	2.27	376	286	3.10	0.45	0.60	1.05
				$\parallel$	1.10	77	291	0.65	0.13	0.35	0.48
				ave	2.15	277	288	2.29	0.34	0.51	0.85
	72-atom (SQS)	4×10^{19}	800	$\perp$	2.08	303	282	2.50	0.32	0.58	0.90
				$\parallel$	1.00	62	287	0.51	0.08	0.33	0.41
				ave	1.95	223	284	1.79	0.24	0.50	0.74
	128-atom (SQS)	4×10^{19}	800	$\perp$	2.18	369	268	2.90	0.40	0.56	0.96
				$\parallel$	1.10	85	264	0.59	0.10	0.34	0.44
				ave	1.99	274	266	1.95	0.30	0.49	0.79
	256-atom (SQS)	4×10^{19}	800	$\perp$	1.84	288	265	2.24	0.31	0.55	0.86
				$\parallel$	0.75	52	264	0.36	0.05	0.33	0.38
				ave	1.67	210	265	1.47	0.23	0.48	0.71
	SnS[2,7]	4×10^{19}	880	$\perp$	1.20	422	233	2.39	0.63	1.05	1.68
				$\parallel$	0.72	72	299	0.64	0.12	0.66	0.78
				ave	1.26	305	255	1.98	0.46	0.92	1.38
	SnSe[2,7]	4×10^{19}	800	$\perp$	2.09	605	256	3.99	0.66	0.84	1.50
				$\parallel$	1.26	134	272	0.99	0.15	0.48	0.63
				ave	2.03	448	262	3.07	0.49	0.72	1.21
n-type	32-atom (enum)	2×10^{19}	800	$\perp$	1.58	206	-300	1.78	0.29	0.61	0.90
				$\parallel$	4.61	502	-343	5.89	0.69	0.33	1.02
				ave	2.56	304	-314	3.00	0.42	0.52	0.94
	32-atom (SQS)	2×10^{19}	800	$\perp$	1.58	192	-304	1.73	0.27	0.60	0.87
				$\parallel$	4.32	513	-336	5.79	0.72	0.35	1.07
				ave	2.53	299	-315	2.96	0.42	0.51	0.93
	72-atom (SQS)	4×10^{19}	800	$\perp$	1.21	189	-261	1.28	0.25	0.58	0.83
				$\parallel$	3.74	788	-294	6.82	1.12	0.33	1.45
				ave	2.22	389	-272	2.88	0.54	0.50	1.04
	128-atom (SQS)	2×10^{19}	800	$\perp$	1.35	159	-293	1.32	0.22	0.56	0.78
				$\parallel$	4.35	527	-343	6.21	0.81	0.34	1.15
				ave	2.40	282	-310	2.70	0.41	0.49	0.90
	256-atom (SQS)	2×10^{19}	800	$\perp$	1.20	136	-290	1.11	0.19	0.55	0.74
				$\parallel$	4.58	439	-344	5.21	0.58	0.33	0.91
				ave	2.26	237	-308	2.25	0.32	0.48	0.80
	SnS[6,7]	4×10^{19}	880	$\perp$	1.10	281	-256	1.77	0.39	1.05	1.44
				$\parallel$	2.61	595	-274	4.47	0.85	0.66	1.51
				ave	1.60	386	-262	2.65	0.54	0.92	1.46
	SnSe[6,7]	2×10^{19}	800	$\perp$	1.50	278	-288	2.24	0.34	0.84	1.18
				$\parallel$	4.25	643	-321	6.62	0.76	0.48	1.24
				ave	2.38	400	-299	3.58	0.48	0.72	1.20

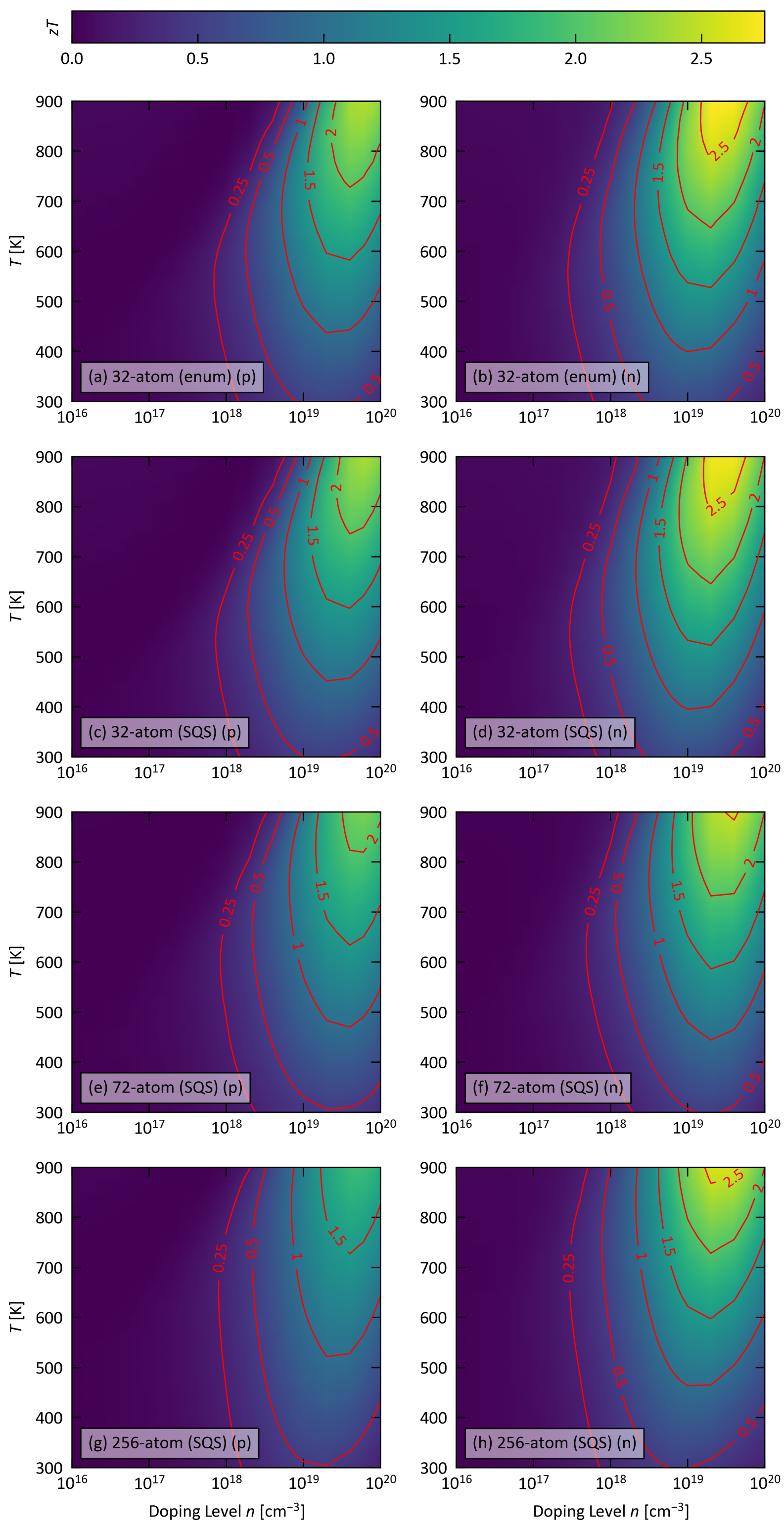

Fig. S22 Predicted thermoelectric figure of merit zT of the p- and n-type 32-atom supercell enumeration and 32-, 72- and 256-atom special quasi-random structure (SQS) models of $Sn(S_{0.2}Se_{0.8})$ as a function of extrinsic electron carrier concentration n ("doping level") and temperature T. The κ_{latt} of all the models was computed using averaged three-phonon interaction strengths $\tilde{P}$ estimated from the endpoints. The contour lines mark zT values of 0.25, 0.5, 1, 1.5, 2, and 2.5.

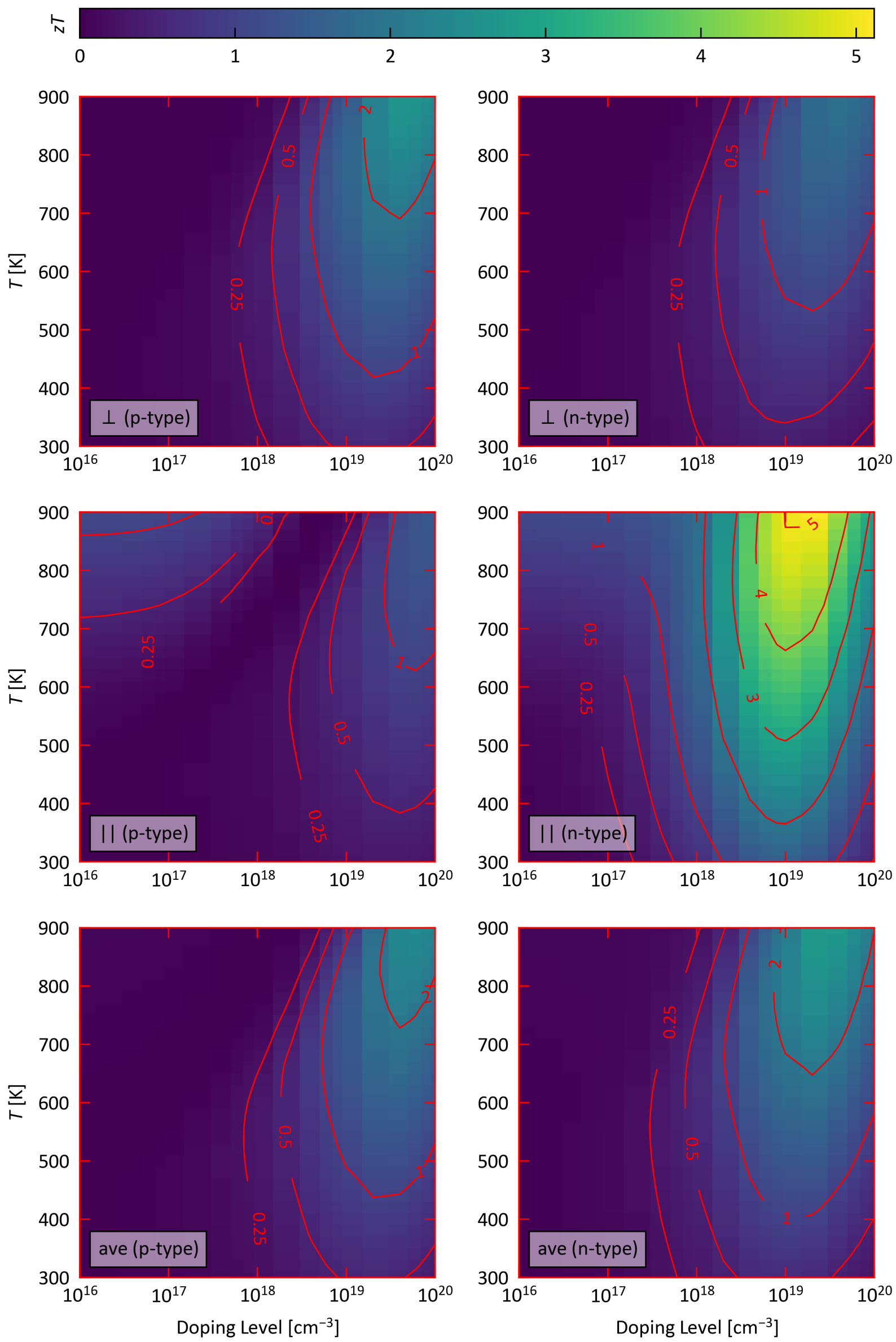

Fig. S23 Predicted scalar-average, in-plane (⊥), out-of-plane (∥) thermoelectric figure of merit zT of the 32-atom supercell enumeration model of $Sn(S_{0.2}Se_{0.8})$ with p- and n-type doping (left/right) as a function of extrinsic electron carrier concentration ("doping level") n and temperature T. The lattice thermal conductivity κ_{latt} was computed using averaged three-phonon interaction strengths $\tilde{P}$ estimated from the endpoints. Values of zT = 0.25, 0.5, 1, 2, 3, 4 and 5 are shown by contour lines.

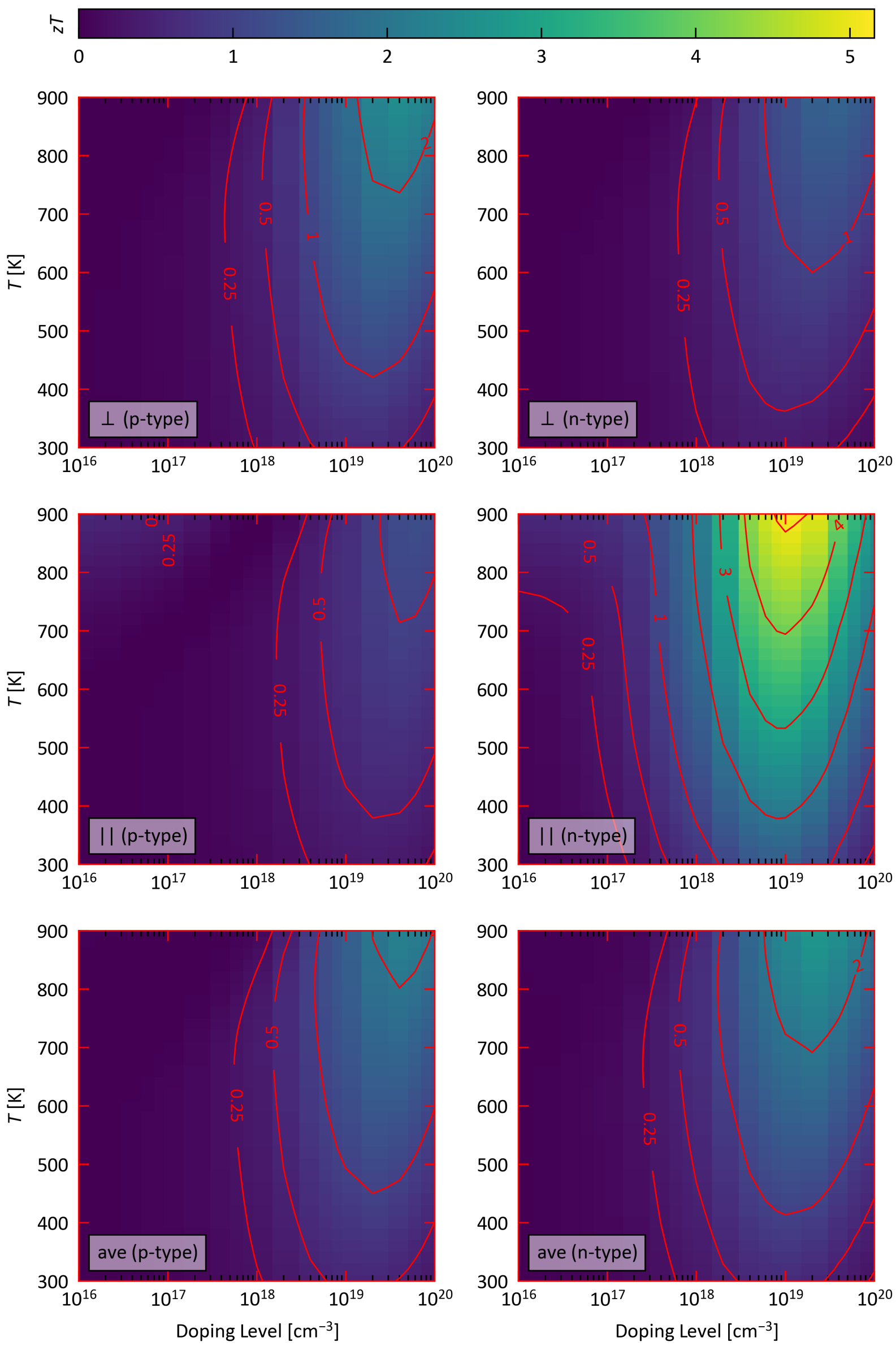

Fig. S24 Predicted scalar-average, in-plane (⊥), out-of-plane (∥) thermoelectric figure of merit zT of the 128-atom special quasi-random structure (SQS) model of $Sn(S_{0.2}Se_{0.8})$ with p- and n-type doping (left/right) as a function of extrinsic electron carrier concentration ("doping level") n and temperature T. The lattice thermal conductivity κ_{latt} was computed using averaged three-phonon interaction strengths $\tilde{P}$ estimated from the endpoints. Values of $zT = 0.25$, 0.5, 1, 2, 3, 4 and 5 are shown by contour lines.

5 References

1 K. Momma and F. Izumi, *Journal of Applied Crystallography*, 2011, **44**, 1272–1276.

2 J. M. Flitcroft, I. Pallikara and J. M. Skelton, *Solids*, 2022, **3**, 155–176.

3 J. M. Skelton, *Journal of Materials Chemistry C*, 2021, **9**, 11772–11787.

4 J. M. Skelton, D. S. Gunn, S. Metz and S. C. Parker, *Journal of Chemical Theory and Computation*, 2020, **16**, 3543–3557.

5 Z. M. Gibbs, F. Ricci, G. Li, H. Zhu, K. Persson, G. Ceder, G. Hautier, A. Jain and G. J. Snyder, *npj Computational Materials*, 2017, **3**, 8.

6 M. Zhang, J. M. Flitcroft, S. K. Guillemot and J. M. Skelton, *Journal of Materials Chemistry C*, 2023, **11**, 14833–14847.

7 M. Zhang, I. Pallikara, J. M. Flitcroft and J. M. Skelton, *Journal of Materials Chemistry A*, 2025, **13**, 5415–5426.
